# Protective effect of Cordyceps sinensis against diabetic kidney disease through promoting proliferation and inhibiting apoptosis of renal proximal tubular cells

**DOI:** 10.1186/s12906-023-03901-4

**Published:** 2023-04-06

**Authors:** Yuhan Zhang, Lusi Xu, Yiran Lu, Jing Zhang, Mengge Yang, Yutian Tian, Jianjun Dong, Lin Liao

**Affiliations:** 1 Department of Endocrinology and Metabology, Shandong Provincial Qianfoshan Hospital, Shandong University, Jinan, China; 2 Department of Endocrinology, Shandong Provincial Hospital Affiliated to Shandong First Medical University, Jinan, China; 3 Department of Endocrinology and Metabology, The First Affiliated Hospital of Shandong First Medical University & Shandong Provincial Qianfoshan Hospital, Jinan, China; 4 Department of Endocrinology, Qilu Hospital, Shandong University, Jinan, China; 5 Clinical Medicine College, Weifang Medical University, Weifang, China

**Keywords:** Cordyceps sinensis, Diabetic kidney disease, Network pharmacology, Molecular docking

## Abstract

**Background:**

Diabetic kidney disease (DKD) has mainly been considered as a glomerular disease. Our previous study showed that the progression of DKD was highly correlated with the dysfunction of renal proximal tubular cells. Fermented Cordyceps sinensis (CS), a substitute for natural CS, is a prominent herb widely used in China, and has exhibited excellent efficacy on DKD. However, the underlying mechanisms remain poorly understood.

**Methods:**

The database analysis was used to identify the main therapeutic targets and pathways of CS involved in DKD treatment. Next, the protective effects of fermented CS on high glucose (HG, 30 mM) induced HK-2 cell injury was validated through cell proliferation and apoptosis assay, including CCK-8, EdU and TUNEL. Finally, quantitative real‑time PCR (qRT-PCR) and western blotting were used to verify key target genes.

**Results:**

Our results revealed that 9 main targets (RELA, JNK1, PTEN, VEGFA, EGF, ERK2, CASP3, AKT1, MMP9) were recognized as key therapeutic targets with excellent binding affinity screened by database analysis and molecular docking. The biological processes were identified by Gene Ontology (GO) enrichment, which appeared mainly involved in the positive regulation of cell proliferation as well as the negative regulation of apoptosis. The verification experiments in vitro revealed that fermented CS significantly attenuated the HG-induced cytotoxicity and apoptosis, and promoted the proliferation of HK-2 cells. Moreover, fermented CS significantly downregulated the expressions of Bax, Caspase-3, VEGFA, P-AKT and P-ERK, and upregulated the expression of PTEN compared with that of HG group.

**Conclusion:**

Our results demonstrate that the fermented CS has nephroprotective effects significantly, which functions via promoting proliferation and inhibiting apoptosis of renal proximal tubular cells, likely by targeting Caspase-3, Bax, VEGFA and PTEN. Furthermore, AKT and ERK signaling pathway may be the critical mechanisms underlying the efficacy of fermented CS in DKD treatment.

**Supplementary Information:**

The online version contains supplementary material available at 10.1186/s12906-023-03901-4.

## Introduction

Diabetic kidney disease (DKD), is the main cause of end-stage renal failure worldwide. It is a chronic microvascular complication of diabetes mellitus (DM), occurring in 20%–40% of patients with diabetes mellitus [1]. However, the pathogenesis of DKD is still under much dispute. Recently, a growing body of evidences indicate that renal tubular cell injury and dysfunction also play a pivotal role in the pathogenesis of DKD [2–5]. Therefore, the renal proximal tubular cells may be a potential therapeutic target for treatments of DKD. However, current treatments of DKD primarily target the renin–angiotensin–aldosterone system (RAAS), with dissatisfied efficacy [6]. Limited therapeutic options are available to prevent DKD progression, and novel complementary medicines are urgently required.

Cordyceps sinensis (CS) is one of the well-known herbs in traditional Chinese medicines (TCM). It is a unique compound naturally formed from the CS (Berk.) Sacc. parasitic on moth larvae and the moth larval bodies. It has protective effects on cardiovascular, liver and endocrine system [7–9]. However, highly specific natural habitat requirements, a high degree of host specificity for moth larvae and long-standing large scale excessive harvesting, have collectively made the naturally grown CS sorely deficient near extinction. Fermented CS is produced through artificial fermenting and purifying of strains isolated from fresh Qinghai CS. Since fermented CS can be produced in much greater quantities, it has gradually become the substitutes for natural CS. Growing evidence shows that fermented CS possesses similar biological activities of natural CS [10, 11]. Some studies have also shown that fermented CS offers renoprotective effects in the diabetic mouse by improvements in hyperglycemia and dyslipidemia [6, 8, 12, 13]. However, the underlying molecular mechanism is not well understood.

In the present study, integrative database analysis and experimental verification were used to reveal the underlying renoprotective mechanism of fermented CS in DKD therapy. Firstly, the potential targets of CS and DKD were predicted by screening various databases. Then the candidate targets and potential mechanisms of fermented CS in the treatment of DKD were identified using functional network and enrichment analysis. Finally, molecular docking and cell experiments were employed to verify these findings.

## Materials and methods

### Screening for the candidate targets of CS in DKD treatment

The bioactive ingredients of CS were obtained from the TCMSP (http://tcmspw.com/tcmsp.php). The filtering thresholds of bioactive ingredients were oral bioavailability (OB) ≥ 30% and drug likeness (DL) ≥ 0.18. At the same time, the targets corresponding to active ingredients from CS were mainly gathered from five databases: Pubchem, pharmmaper, Swiss Target Prediction, STITCH and TCMSP.

The genes related to DKD were gathered from six existing databases: GeneCards database (https://www.genecards.org/), OMIM database (https://www.omim.org/) and DisGeNET (https://www.disgenet.org/), CTD, TTD, DRUGBANK. Then, target genes for DKD were obtained by merging the database search results and deleting duplicate targets. Noteworthy, all the targets names were selected by Homo Saipan species and put into the UniProt Knowledgebase (http://www.uniprot.org/) to normalize the gene information. Next, the potential targets of CS mapped to DKD-related genes to further reveal the therapeutic role and candidate targets of CS in DKD.

### Network construction, pathway analysis and molecular docking

The protein interaction information of candidate targets was obtained from the String database (http://string-db.org/). Among them, the data of intersection target protein was carried on for constructing the protein–protein interaction (PPI) network diagram using Cytoscape version 3.6.1. Also, the topological features of every node in the interaction network was analyzed to further extract hub genes in the network, which the targets with degree > twofold median were selected as the hub genes. Ultimately, a core subnetwork was built for the ensuing analysis.

In order to further reveal the underlying mechanism of CS in DKD therapy, the overlapping targets were imported into the functional annotation tool of Database for Annotation, Visualization and Integrated Discovery (DAVID) 6.8 (https://david.ncifcrf.gov/) to perform GO functional analysis. Notably, a P value less than 0.05 was set as the screening threshold. Then, the top 10 biological process meeting the criterion were displayed as the bubble chart.

Molecular docking was applied to explore the predicted binding modes of the screened candidate targets and compounds of CS. The crystal structures of target proteins were downloaded from RCSB Protein Data Bank (http://www.pdb.org/). The binding affinity of compound-target relationship were verified by Autodock Vina software, a freely available open-source packages. The binding models of the docking affinity in the top 5 pairs was visualized by PyMol2.3.0 software and Discovery Studio3.5 software.

### Cell culture

HK-2, a proximal tubular cell line derived from normal human kidney (American Type Cell Collection, Rockville, MD) was cultured in RPMI 1640 medium supplemented with 11.1 mM D-glucose, 10% fetal calf serum (Gibco, USA), 100 U/ml penicillin and 100 μ g/ ml streptomycin (Sigma, St. Louis, MO) at 37℃, 5% CO_2_ and 95% humidity. For most trials, the cells were cultured in medium containing 5.5 mM normal glucose + 24.5 mM mannitol (MG), 30 mM high glucose (HG) or 30 mM high glucose added to 50 μg/mL fermented Cordyceps sinensis (HG + CS) for 48 h.

### Antibodies, reagents and Cordyceps sinensis

Antibodies used in this study include: antibodies against Bax (1:1000, Affinity), Caspase-3 (1:1000, CST), VEGFA (1:1000, Affinity), total ERK (1:10,000, abcam), phosphorylated ERK (1:1000, abcam), total AKT (1:1000, PTG), phosphorylated AKT (1:1000, CST), total JNK (1:1000, Affinity), phosphorylated JNK (1:1000, Affinity), and GAPDH (1:10,000, PTG). All secondary antibodies (ployclonal, goat anti-rabbit IgG) were from Jackson ImmunoResearch Laboratories Inc. (West Grove, PA). Unless indicated, other reagents were from Sigma (St. Louis, MO).

The fermented Cordyceps sinensis (Cs‑C‑Q80) is produced by a medicinal strain isolated and extracted from natural CS with artificial fermentation processing. It has similar chemical composition and pharmacological activities to the wild Cordyceps sinensis. It contains nucleosides, amino acids and mannitol, as listed in the standard of the pharmacopoeia of the people’s Republic of China (2015 Edition) [14]. The fermented CS capsule were supplied by Hangzhou Zhongmei Huadong Pharmaceutical Co. Ltd. (Hangzhou, China), and the concentration of it is 1 g/ml.

### Cell viability and proliferation analysis

CCK-8 assay was performed to evaluate cell viability. HK-2 cells were cultivated in 96-well plates at a density of 3.0 × 10^4^ cells/cm^2^. Cells were treated with MG, HG or HG supplemented with fermented CS at various concentrations (10, 50, 100, 200 μg/mL). At 24, 48, 72 h after the treatment, 10 µL of Cell Counting Kit-8 solution (Dojindo Molecular Technologies, Inc., Kumamoto, Japan) was added to each well, and the cells were incubated in a humidified CO_2_ incubator at 37℃ for additional 3 h. Optical density values (OD) at wavelength 450 nm were measured with a microplate reader (Multiskan™ FC, Thermo Fisher Scientific, Inc., Waltham, MA). The cell survival rate was calculated according to the manufacturers’ instructions.

The proliferation of HK-2 cells was determined by a 5-ethynyl-2-deoxyuridine (EdU) labeling/detection kit (RiboBio, China) according to the manufacturer's recommendations. HK-2 cells were seeded into 6 well plates containing sterile glass coverslips and stimulated with MG, HG or HG + fermented CS (50 μg/mL) for 48 h. Nuclei were counterstained with DAPI. Immunofluorescence images were viewed and captured using a Leica microscope (at a magnification of 20 or 40), and the ratio of EdU-positive cells (with red fluorescence) to DAPI-stained cells (with blue fluorescence) were used to assess the proliferation activity of HK-2 cells.

### TUNEL staining

TUNEL assay was used to analyse the effects of fermented CS on HG induced apoptosis of HK-2 cells. Cells were cultured overnight in 6-well plates and were then exposed to MG, HG or HG + fermented CS (50 μg/mL), respectively. After 48 h, the apoptotic cells were observed under the fluorescence microscope. The apoptosis rate of HK-2 cells was calculated according to the manufacturer’s instructions. Analysis was performed following at least three independent experiments.

### Quantitative real‑time PCR (qRT-PCR)

The mRNA expression of target genes was measured by quantitative real‑time PCR (qRT-PCR). Total RNA was extracted from cultured HK-2 cells with TRNzol-A + RNA isolation reagent (TIANGEN) according to the manufacturer’s instructions. Reverse transcription was conducted with 1 μ g of total RNA and RevertAid First Strand cDNA Synthesis Kit (Fermentas). To detect the mRNAs of target genes, oligonucleotide primers were used as shown in Table 1. qRT-PCR was optimized and performed in a cycler (MyiQ2, Bio-Rad) using SYBR green (Roche). The amount of qRT-PCR products was normalized with GAPDH mRNA to analyze the relative expression ratio for target gene mRNA using the 2^‑∆∆Ct^ method. Each experiment was repeated three times to ensure amplification integrity.

**Table 1:** Primer sequences

mRNA/primer direction	Sequence
VEGFA-F	GCAGAATCATCACGAAGTGGT
VEGFA-R	CCAGGGTCTCGATTGGATGG
MMP9-F	TCTGCCCGGACCAAGGATA
MMP9-R	ACATAGGGTACATGAGCGCC
PTEN-F	CTCAGCCGTTACCTGTGTGT
PTEN-R	AGGTTTCCTCTGGTCCTGGT
Caspase-3-F	GACTGCGGTATTGAGACAGA
Caspase-3-R	CGAGTGAGGATGTGCATGAA

### Western blot

Protein levels were measured by Western blotting. Treated HK-2 cells were harvested and collected as cell pellets, and lysed in ice-cold lysis buffer to extract the protein. Their total protein content was measured by bicinchoninic acid assay (Sigma) according to the manufacturer’s instructions. An equal amount of protein from each cell group was processed for SDS-PAGE and electrotransferred to PVDF membranes (Bio-Rad Inc.). Then the PVDF membranes were incubated in blocking buffer with 5% non-fat milk in TBST buffer (0.1% Tween 20, 0.2 mM Tris, and 137 mM NaCl) at room temperature for 1 h, and then probed overnight at 4 °C with corresponding primary antibodies. It’s worth noting that all the blots were cut prior to hybridisation with primary antibodies to simultaneously incubate different antibodies of similar molecular weight. The membranes were then rinsed with TBST for 3 times, and then incubated with conjugated secondary antibody (1:10,000, goat anti-rabbit IgG) at room temperature for 1 h. Antibody-antigen complexes were visualized with Alpha chemiluminescent gel imaging system FluorChem E (ProteinSimple, San Jose, CA), and were analyzed quantitatively by densitometry with Image J software (National Institutes of Health, Bethesda, MD). The relative density of immunoreactive bands was normalized against GAPDH.

### Statistical analysis

Experiments were carried out at least in triplicate and data were expressed as mean ± SEM. All data were subjected to statistical analysis using SPSS (Statistical Product and Service Solutions) 19.0 software (from IBM). GraphPad Prism 8.0.1 software was used to draw statistical charts. Student's t-tests was used to compare differences between groups while multiple statistic comparisons were analyzed by one-way analysis of variance (ANOVA) followed by post hoc analysis. A difference with *p* < 0.05 was considered statistically significant.

## Results

### Identified the putative therapeutic targets of CS against DKD

**Fig. 1:**
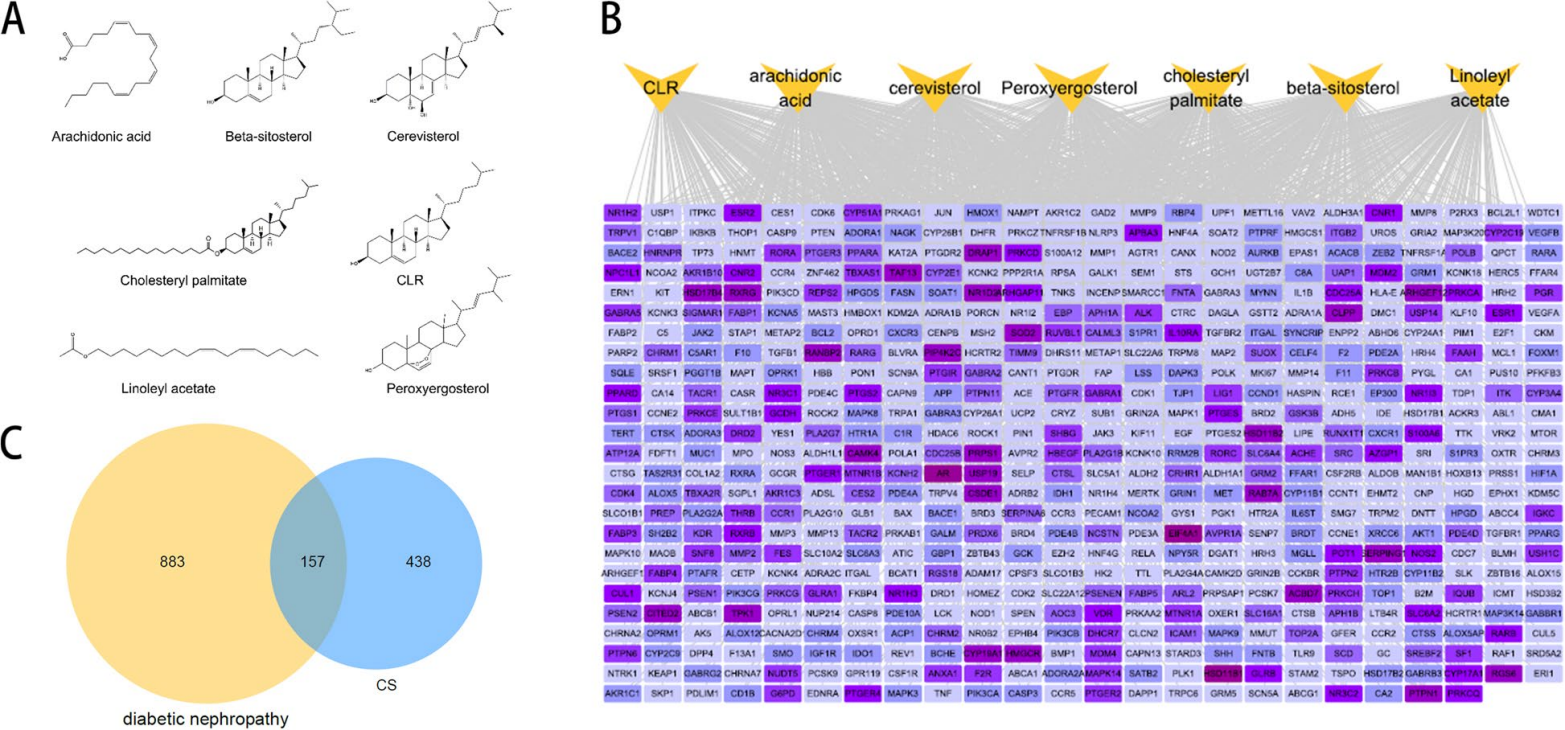
Identification of common targets of CS and DKD. **A** Chemical structures of seven 7 active compounds of CS. **B** Compound-target network. Yellow triangle nodes represent active compounds of CS. Purple rectangle nodes represent corresponding targets which the darker the colour, the higher the degree value. **C** Venn diagrams demonstrate the number of intersection targets of CS and DKD

Firstly, using standard conditions with the thresholds of OB ≥ 30% and DL ≥ 0.18 properties, we found a totally of 7 bioactive components of CS via the TCMSP databases, which were Cholesteryl palmitate, Linoleyl acetate, Arachidonic acid, Beta sitosterol, Cerevisterol, Peroxyergosterol and CLR (Fig. 1A). We also identified 595 targets corresponding to active ingredients from CS through five databases (Fig. 1B). Secondly, the DKD-related targets were retrieved and a total of 1040 targets were obtained after removing the duplicate values. Finally, the potential targets of CS were matched to the DKD-related targets. A total of 157 overlapping targets were selected as putative therapeutic targets for CS in DKD treatment (Fig. 1C).

### Protein–Protein Interaction (PPI) network construction, Gene Ontology (GO) enrichment analysis

**Fig. 2:**
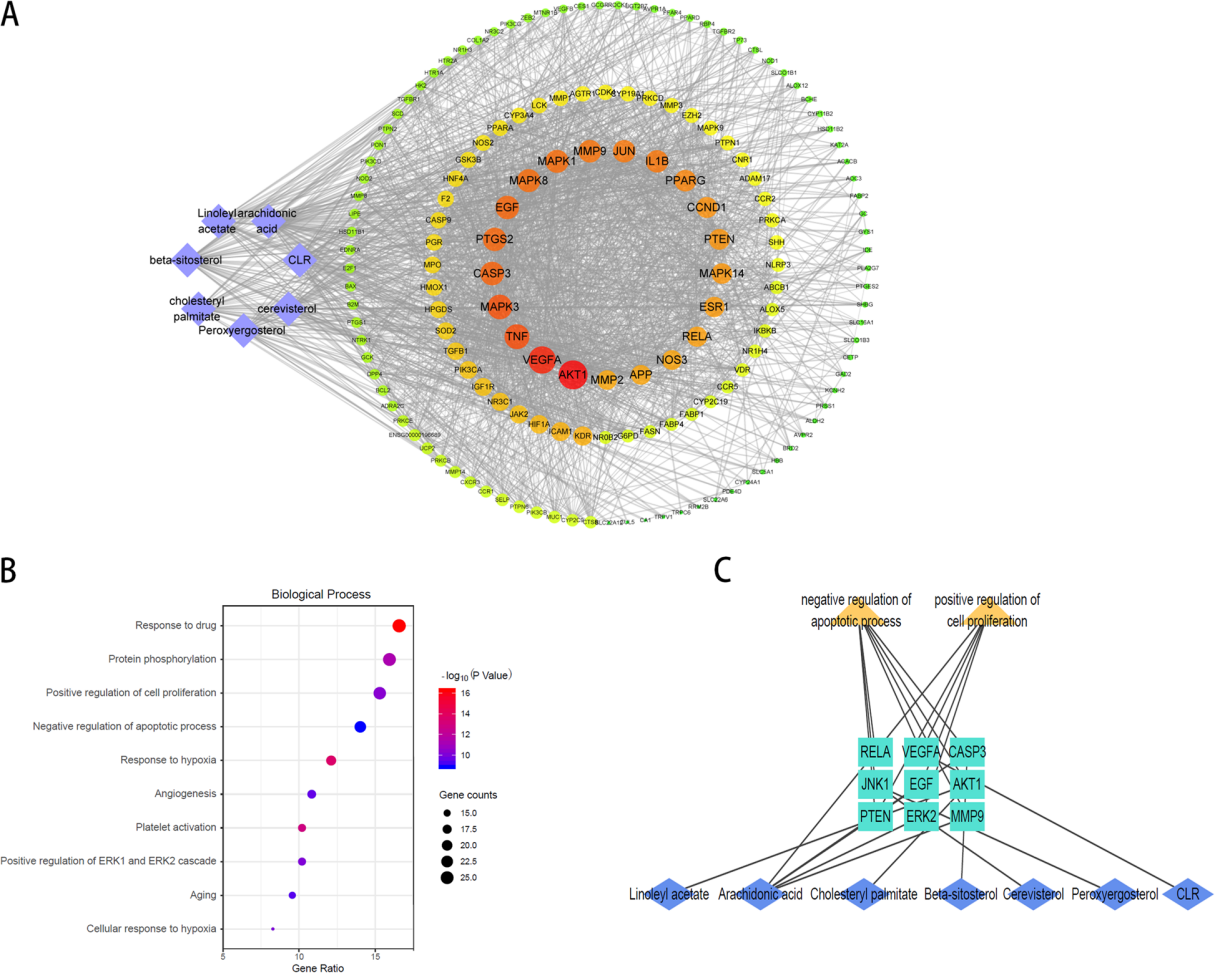
The PPI network and GO enrichment analysis. **A** Purple diamond nods represent the active compounds of CS and circle nodes represent the 157 targets; the innermost circle of the concentric circle shows the 21 hub genes; Edges represent protein (node) interactions and the sizes and colours of the nodes are illustrated from big to small and yellow to red in descending order of degree value. **B** Biological process in enrichment analysis of GO pathway. **C** Sub-network was constructed of compounds, key biological processes and corresponding targets

To investigate the key targets of CS in DKD treatment, 157 overlapping targets were used to product PPI network. Additionally, 21 hub genes were screened by the “degree” value of the topological analysis. A compound-target network was built to better demonstrated the relationship between the putative therapeutic targets of CS and the active compounds (Fig. 2A). Then, GO functional annotation and enrichment analyses of 157 potential targets were used to identify the underlying mechanism of CS in DKD treating. The top 10 GO items were selected based on counts of hit genes and *P* values (Fig. 2B). For biological processes, it can be found that the targets were mainly enriched in response to drugs, protein phosphorylation, positive regulation of cell proliferation and negative regulation of the apoptotic process. Subsequently, as is shown in the Fig. 2C, a sub-network was extracted to demonstrate the interaction between key biological process (positive regulation of cell proliferation and negative regulation of the apoptotic process) and corresponding targets. A total of 9 key targets associated with proliferation and apoptosis process, which were RELA, JNK1, PTEN, VEGFA, EGF, ERK2, CASP3, AKT1, MMP9. Additionally, all compounds of CS could regulate cell proliferation an apoptosis, suggesting the possible benefit effects of CS on DKD.

### CS bioactive compounds showed high affinity to key targets in DKD

**Fig. 3:**
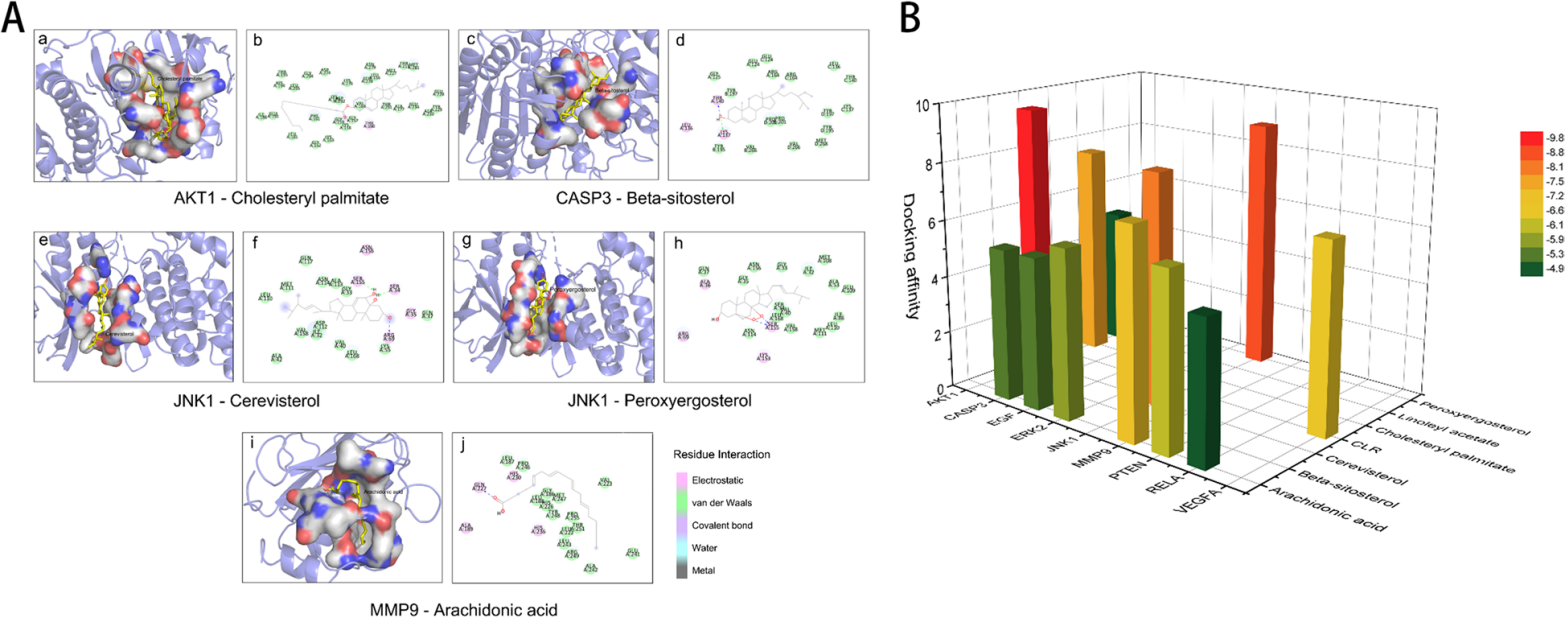
Molecular docking models of compounds binding to key targets. **A** The top 5 pairs of molecular docking models. Figure (a) is the 3D presentation of interaction between active compounds and related-targets and figure (b) shows the corresponding 2D structure. **B** 3D column diagram shows the docking affinity of the top 5 pairs models. X-axis: protein names, Y-axis: active compounds, Z-axis: the absolute value of the docking affinity

In the present studies, the possible interaction activity between 9 key genes and their corresponding compounds of CS was investigated with molecular docking verification. The greater the absolute value of the docking affinity, the stronger binding ability between the compounds and the active site of the targets. As shown in Table 2, most of the docking affinity are less than − 5.1 kcal/mol except for docking pairs of Linoleyl acetate- AKT1 and Arachidonic acid-RELA, indicating that these bioactive compounds of CS possessed high binding affinity with the corresponding key targets. The modes of top 5 binding complexes are displayed in Fig. 3.

**Table 2:** Molecular docking results of 9 proliferation-apoptosis related genes and corresponding compounds of CS

Number	Hub genes	PDB ID	Compound	Docking affinity(Kcal/mol)
1	AKT1	4EKL	Cholesteryl palmitate	-7.5
			Linoleyl acetate	-4.9
2	CSAP3	3GJQ	Arachidonic acid	-5.3
			Beta sitosterol	-9.8
3	EGF	2KV4	Arachidonic acid	-5.3
4	JNK1	3PZE	Cerevisterol	-8.1
			Peroxyergosterol	-8.8
5	MAPK1	6RQ4	Arachidonic acid	-5.9
6	MMP9	6ESM	Arachidonic acid	-7.2
7	PTEN	1D5R	Arachidonic acid	-6.1
8	RELA	1NFI	Arachidonic acid	-4.9
9	VEGFA	1VPF	CLR	-6.6

### *Fermented CS attenuated HG-induced apoptosis and promote proliferation in HK-2 cells*.

**Fig. 4:**
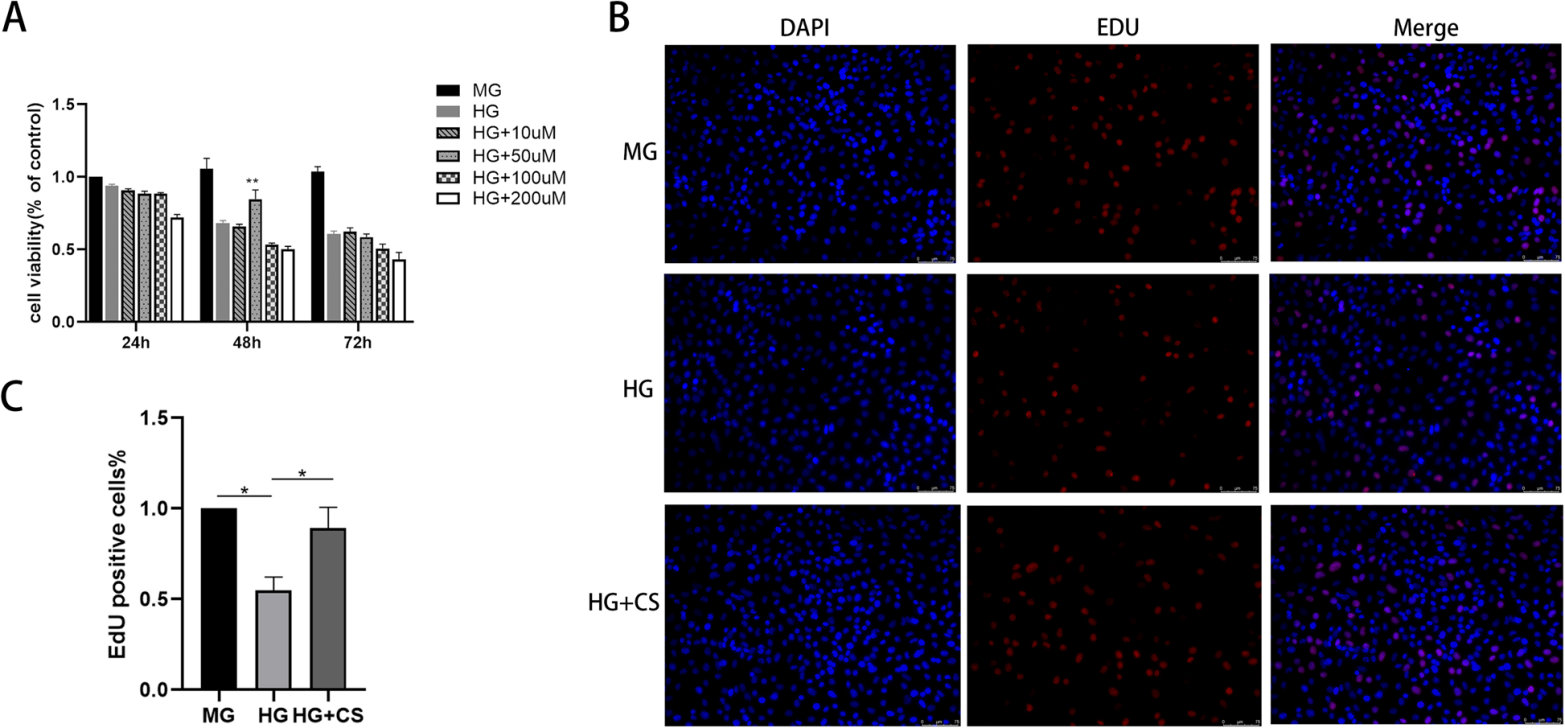
Fermented CS improved the cell viability and proliferation of HK-2 cells induced by HG. **A** Cell viability induced by different concentrations of CS. **p < 0.01, versus the HG group. **B**-**C** The cell proliferation was evaluated by EdU staining assay. Results were presented by mean ± SEM. *p < 0.05, compared with the HG group. Proliferating cells were labelled with EdU (red fluorescence). The nuclei were labbled with DAPI (blue)

The CCK8 assay was performed to examine the cytotoxic effects of HG and possible protective effects of fermented CS on HK-2 cells. The HK-2 cells were treated with different concentrations of fermented CS (10, 50, 100, 200 μg/mL) in a HG environment. Additionally, the MG group and HG group were used as control groups. As shown in Fig. 4A, the cell proliferation in HG group was decreased compared to that in MG group (*p* < 0.05). The proliferation of cells treated with fermented CS restored gradually. This protective effect was significant in the HG + fermented CS group at the concentration of 50 μg/mL at 48 h (*p* < 0.01), which was selected as the optimal intervention concentration and intervention time of fermented CS for subsequent experimentations.

The EdU assay was also used to assess HK-2 cells proliferation. The results showed that the EdU-positive cells were dramatically reduced in HG-treated cells compared with MG group, while fermented CS treatment increased EdU-positive cells (*p* < 0.05, Fig. 4B-C). These data demonstrated that fermented CS could significantly protect HK-2 cells from damages caused by high glucose.

**Fig. 5:**
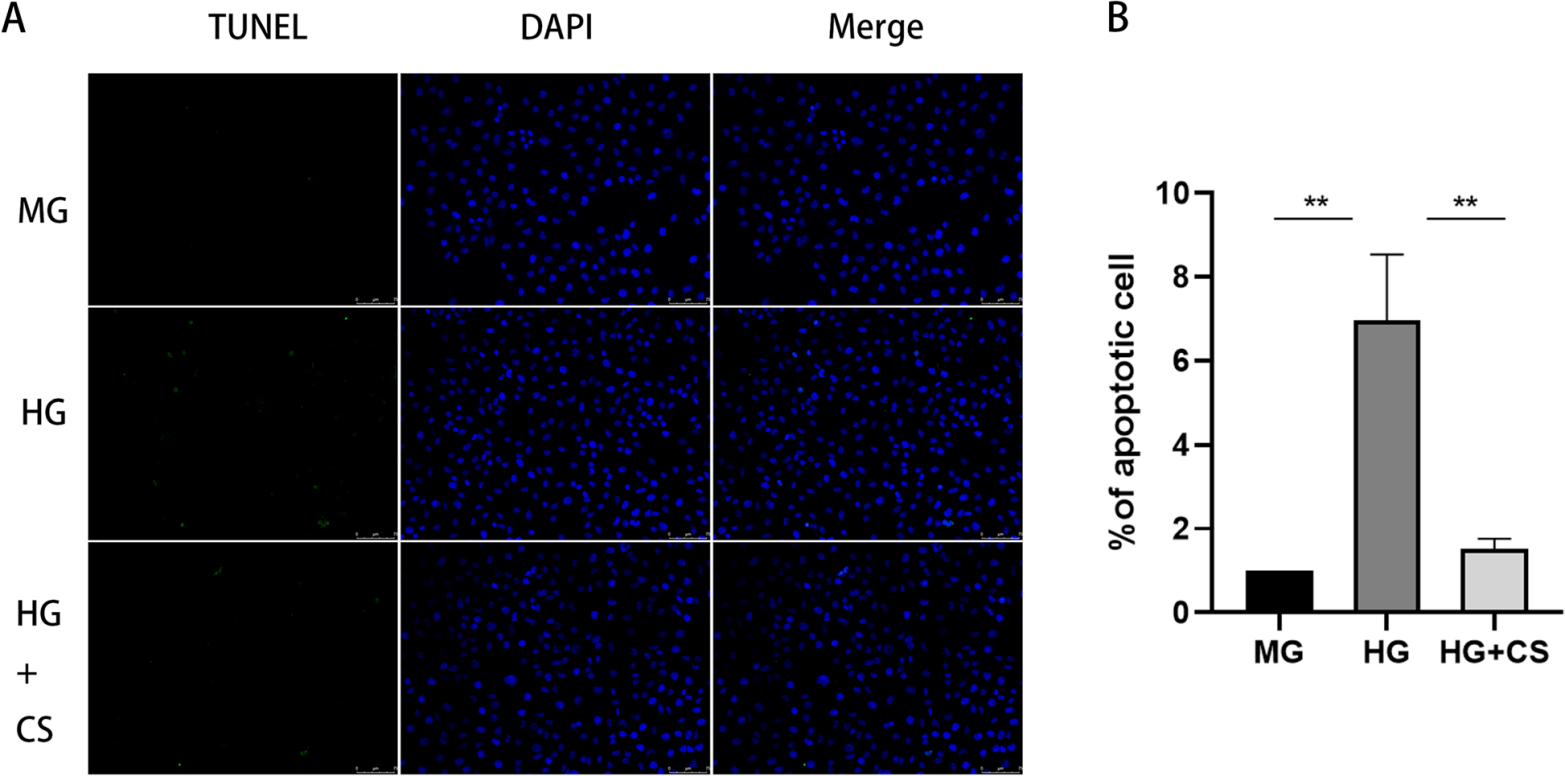
Fermented CS attenuated the apoptosis of HK-2 cells under HG stimulation. **A** TUNEL staining. Apoptosis cells were labelled with green fluorescence. The nuclei were labelled with DAPI (blue). Scale bar, 75 μm. **B** Quantification of the apoptosis rate. Values were mean ± SEM. **p < 0.01 vs the HG group

**Fig. 6:**
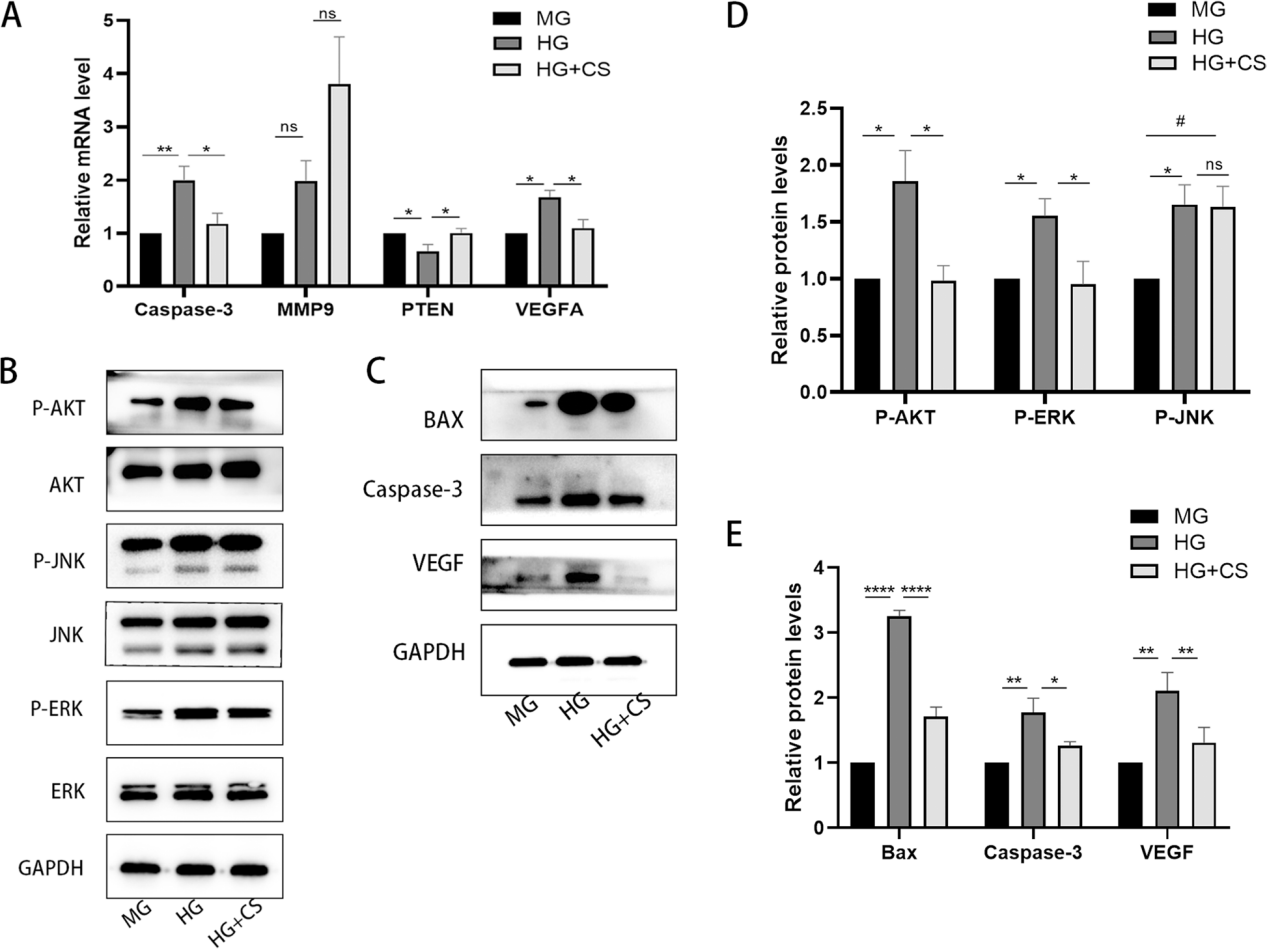
qRT-PCR and Western blotting results. **A** The mRNA level expressions of Caspase-3, MMP9, PTEN and VEGFA in the HK-2 cells were evaluated by qRT-PCR. **B**-**C**. Western blotting analysis of P-AKT/AKT, P-JNK/JNK, P-ERK/ERK, BAX, Caspase-3 and VEGF. GAPDH acts as an internal reference. (P: phosphorylated.) **D**-**E**. Quantification of the western blotting results. Data was presented as the means ± SEM. *p < 0.05, **p < 0.01, ***p < 0.001, ****p < 0.0001, ns *p* > 0.05, compared with the HG group; #*p* < 0.01, compared with MG group

We next investigated the protection effect of fermented CS on the apoptosis of HK-2 cells using TUNEL assay. After 48 h of treatment, the apoptosis of cells exposed to HG significantly increased compared with that in the MG group. However, fermented CS effectively reduced the apoptosis rate of cells compared to HG group (*p* < 0.001, Fig. 5A-B). In addition, the expression of apoptosis-related proteins was evaluated by western blotting and qRT-PCR. The results indicated that the protein and mRNA expression level of Bax and Caspase-3 were obviously increased in the HG group compared with that in the MG group, whereas fermented CS reduced the expression of these apoptosis-related proteins compared with to HG group (*P* < 0.5, Fig. 6). These results indicated that fermented CS alleviates HK-2 cells apoptosis induced by HG.

Moreover, we evaluated three other key targets, VEGF, MMP9 and PTEN. The results showed that the protein and mRNA expression levels of VEGF were significantly increased in the HG group as compared with MG group (*P *< 0.05). On the contrary, fermented CS treatment significantly reduced both the expression and the translation levels of VEGF (*P* < 0.001, Fig. 6). Furthermore, we found that high glucose also elevated the mRNA expression level of MMP9, while it was not affected when co-incubated with fermented CS (Fig. 6A). Fermented CS elevated the mRNA expression level of PTEN compared with that in HG group (*P* < 0.05, Fig. 6A).

Taken together, our in vitro experiment findings indicated that fermented CS may mitigate damage of HK-2 cells induced by high glucose through inhibiting Bax, caspase-3 and VEGF expression, and elevating PTEN expression, which was consistent with our results of the network analysis shown above.

### Fermented CS facilitates proliferation and attenuates apoptosis through the AKT and ERK signaling pathways in HG treated HK-2 cells

Based on the results of network analysis shown above, we selected the indexes closely related to these pathways in our verification experiments, including the AKT, ERK and JNK signaling pathway. To investigate the response of HK-2 cells to HG stimulation, we also examined the phosphorylation of AKT, ERK and JNK. After incubation with HG for 48 h, as shown in Fig. 6B, the expression levels of P-AKT, P-ERK, and P-JNK were significantly increased after HG stimulation. The phosphorylation of AKT and ERK were markedly decreased compared to that of the HG group (*P* < 0.05, Fig. 6D). Nevertheless, the HG-induced cells treated with fermented CS did not produce obvious changes in phosphorylated JNK expressions (Fig. 6B). Combining these findings with the results of previous network analyses, we suggest that fermented CS may promote proliferation and alleviate apoptosis of HK-2 through the AKT and ERK pathways.

## Discussion

As a common and serious complication of DM, DKD has become one of the leading causes of end-stage renal disease (ESRD), resulting in a tremendous clinical and economic burden [15, 16]. It is estimated that the total number of DM patients will increase to 693 million by 2045 [17]. Therefore, there is an urgent need for finding effective treatments for DKD. At present, studies have shown that the apoptosis of renal proximal tubular cells contributes to the hyperglycemia-induced kidney impairment [18]. Similarly, our previous studies demonstrated that the apoptosis of renal proximal tubular cells in diabetic kidneys may occur early, and may be seen both in vitro or *vivo* [19, 20]. Given these findings, we believe targeting apoptosis of renal proximal tubular cells could be a therapeutic option for DKD.

Traditional Chinese medicine (TCM) has been recognized to possess satisfactory efficacy in treating DKD as a complementary and alternative medicine [21]. CS has been widely used in TCM for thousands of years. It has been reported to have a variety of biological activities, including immune-regulation, lipid metabolism modulation, and acting as an antioxidant [10, 16, 22]. In addition, the ability of CS in inhibiting apoptosis has been investigated extensively, and may offer great promise [23–25]. Owing to its similar chemical composition, fermented CS, is now commonly used as a substitute for the wild CS [26]. In addition, studies have shown that oral administration of fermented CS capsules can achieve satisfactory effectiveness in kidney diseases, and reduce kidney damage to improve renal function [27, 28, 29]. Nonetheless, the underlying mechanism of the protective effects of fermented CS in DKD remains elusive. Thus, in this investigation, we sought to elucidate the molecular mechanisms of the protective effect of fermented CS against HG-induced cytotoxicity and apoptosis in HK-2 cells. We found that the proliferation of HK-2 cells was restored gradually after fermented CS treatments and fermented CS significantly inhibited cell apoptosis.

Considering the multicomponent and multitarget characteristics of CS, we used a network pharmacology approach, to investigate the underlying mechanism of CS in DKD therapy. It was predicted that CS could regulate cell proliferation or apoptosis via 9 targets (RELA, JNK1, PTEN, VEGFA, EGF, ERK2, CASP3, AKT1 and MMP9). Based on the results of network pharmacology analysis, cell experiments were used to validate the predicted mechanisms. In the present study, we examined HG-induced toxicity in proximal renal tubular cells in vitro. TUNEL and EdU experiments revealed that fermented CS produced a reversal of apoptosis and proliferation-promoting effect on HK-2 cells induced by HG, suggesting that fermented CS may be effective in the treatment of DKD.

Many evidences suggested that the activation of caspase triggers the apoptotic process in various cells [30, 31]. In addition, the activated BAX protein could damage the outer mitochondrial membrane, and promote mitochondrial membrane permeability. Consequently, the release of cytochrome c from the mitochondria triggers the activation of the initiator caspase-3, setting off a chain of events leading to the eventual destruction of the cell [32]. Based on the cell experiment, our results illustrated that the increased apoptosis of HK-2 cells in HG stimuli was accompanied by significant upregulations of the Bax and Caspase-3 levels. However, after administration with fermented CS, the apoptosis of HK-2 cells induced by HG was alleviated, accompanied by down-regulated Bax and Caspase-3 expression, confirming the above-mentioned effect of fermented CS in reducing apoptosis of renal tubular epithelial cells.

PTEN is a negative regulator of PI3K/AKT signal pathway, which could specifically block the phosphorylation of PI3K on AKT, and inhibit the activity of AKT, exerting the anti-fibrosis effects [33]. Upregulating the expression of PTEN can inhibit the expression of α-SMA and other target genes mediated by PI3K/AKT signal pathway, and reduce renal interstitial fibrosis [34]. In addition, the AKT signaling pathways had been shown to play important roles in DKD. It is reported that the inhibition of the PI3K-AKT signaling pathway activated the autophagy of podocyte, alleviating the progression of DKD [35]. We found that fermented CS could reduce the expression of P-AKT, and improved the expression of PTEN in HK-2 cells, illustrating that fermented CS exhibits a mitigating effect on proximal tubular cells damage under HG environment in DKD.

It is reported that under the hyperglycaemic state of diabetes, renal tissues are hypoxic, leading to high expression of VEGF and HIF-1 [36]. Recent studies suggested that the expression of VEGF was upregulated in diabetic Sprague–Dawley rats [37]. Consequently, the elevation of VEGF was used as an effective biomarker for the early stage of vascular damages in diabetes. In our present study, the expression of VEGF was elevated under HG stimulation in HK-2 cells, and it was significantly decreased when treated by fermented CS, further demonstrating the protective effect of fermented CS in DKD.

MAPK family activation is a key modulator of various kinds of cells in the progression of kidney, including tubular epithelial cells. Wei et al. [38]. demonstrated that the p38 MAPK and the ERK signaling pathways were activated in HK-2 cells after HG stimulation. Similarly, our results confirmed that the ERK and JNK signaling pathways were activated in HG-induced HK-2 cells. However, our results showed that the fermented CS treatment significantly suppressed the phosphorylation ERK, while the JNK phosphorylation was not affected in vitro. Therefore, fermented CS might exert a beneficial effect on attenuating DKD progression by suppressing AKT and ERK pathways.

There are serval limitations in our research. Firstly, the lack of animal model makes it difficult to completely elucidate the protective efficacy of fermented CS on DKD progression, especially renal proximal tubule cells injury. Secondly, further investigations are needed to reveal the upstream mechanism of the anti-apoptosis effects of fermented CS in DKD. Thirdly, though network pharmacological analysis showed that CS might alleviate DKD through MMP9 and JNK pathways, there was no significant difference between the HG group and the HG + CS group. Therefore, further validation is required with other types of kidney cells, like podocyte. Despite these limitations, no previous studies have investigated the anti-apoptotic effects of fermented CS on renal proximal tubular cells in DKD. Fortunately, the database analysis has become an assessment tool to provide comprehensive insights into the underlying mechanisms of drugs, and further point out directions for our further research.

In the current study, we investigated that fermented CS ameliorated cytotoxicity and apoptosis of renal proximal tubular cells induced by HG, and database analysis uncovered the multi-component, multi-target, and multi-pathway potential mechanism underlying the action of fermented CS in DKD. Further, our results illustrated that fermented CS treatment might inhibit the cell apoptosis and improve proliferation through AKT and ERK signaling pathway. Thus, fermented CS as a promising and candidate agent in treatments of DKD, our study provides a new idea for DKD therapy and proposes a robust theoretical basis for the clinical application. However, further study is required to confirm the beneficial effects of fermented CS in vivo as well as more in-depth study of molecular mechanism.

## Supplementary Information

**Additional file 1: Table S1. **The putative targets of CS.

**Additional file 2: Table S2.** DKD related targets.

**Additional file 3: Table S3.** Information for overlapped targets after PPI analysis.

## Data Availability

All data generated or analyzed during this study are available from public databases, published articles and supplementary materials. The TCMSP database (http://lsp.nwu.edu.cn/tcmsp.php); the PubChem database (http://pubchem.ncbi.nlm.nih.gov); the Swiss Target Prediction database (http://www.swisstargetprediction.ch/); the STITCH database (http://stitch.embl.de/); Uniprot sites (http://www.uniprot.org/); the Therapeutic Target Database (TTD, https://db.idrblab.org/ttd/); the Online Mendelian Inheritance in Man database (OMIM, http://omim.org/); the Comparative Toxicogenomics Database (CTD, http://ctdbase.org/); the DrugBank database (https://www.drugbank.ca/); the GeneCards database (https://www.genecards.org/); STRING tools (https://string-db.org/); the Functional Annotation tool of Database for Annotation, Visualization and Integrated Discovery (DAVID) 6.8 (https://david.ncifcrf.gov/); the Cytoscape3.6.1 (http://cytoscape.org/); the AutoDock Tools1.5.6(http://mgltools.scripps.edu/documentation/links/autodock); RCSB Protein Data Bank (http://www.pdb.org/); Detailed information is described in Table S1, S2 and Table S3.

## Abbreviations

CS: Cordyceps sinensis
DKD: Diabetic kidney diease
qRT-PCR: Quantitative real‑time PCR
GO: Gene Ontology
TCM: Traditional Chinese medicine
PPI: Protein–protein interaction
OB: Oral bioavailability
DL: Drug likeness
OD: Optional density values
