# Supplementary material for: Protective effect of Cordyceps sinensis against diabetic kidney disease through promoting proliferation and inhibiting apoptosis of renal proximal tubular cells
Source: BMC Complement Med Ther. 2023 Apr 6;23:109. doi: 10.1186/s12906-023-03901-4 (PMC10077712; doi:10.1186/s12906-023-03901-4)
Supplement: Supplementary file 1 — Additional file 1: Table S1. The putative targets of CS. [file 12906_2023_3901_MOESM1_ESM.docx]

**Table S1** The putative targets of CS.

| Nnumber | Gene name | Uniprot ID | Protein name | Database |
| --- | --- | --- | --- | --- |
| 1 | ABAT | P80404 | 4-aminobutyrate Aminotransferase | SymMap |
| 2 | ABCA1 | O95477 | ATP Binding Cassette Subfamily A Member 1 | SymMap/TCMSP |
| 3 | FNTA | P49354 | Protein farnesyltransferase/geranylgeranyltransferase type-1 subunit alpha | SwissTargetPrediction |
| 4 | GRIN1 | Q05586 | Glutamate receptor ionotropic, NMDA 1 | SwissTargetPrediction |
| 5 | GRIN2A | Q12879 | Glutamate receptor ionotropic, NMDA 2A | SwissTargetPrediction |
| 6 | PGGT1B | P53609 | Geranylgeranyl transferase type-1 subunit beta | SwissTargetPrediction |
| 7 | ABCB1 | P08183 | P-glycoprotein 1 | SwissTargetPrediction |
| 8 | ABCC4 | [O15439](https://www.uniprot.org/uniprot/O15439) | Multidrug resistance-associated protein 4 | TCMSP/SymMap |
| 9 | ABCG1 | [P45844](https://www.uniprot.org/uniprot/P45844) | ATP-binding cassette sub-family G member 1 | TCMSP/SymMap |
| 10 | ABHD6 | Q9BV23 | Monoacylglycerol lipase ABHD6 (by homology) | SwissTargetPrediction |
| 11 | ABL1 | P00519 | Tyrosine-protein kinase ABL | SwissTargetPrediction |
| 12 | ACACB | O00763 | Acetyl-CoA carboxylase 2 | SwissTargetPrediction |
| 13 | ACBD7 | Q8N6N7 | Acyl-CoA-binding domain-containing protein 7 | PharmMapper |
| 14 | ACE | P12821 | Angiotensin-converting enzyme | SwissTargetPrediction |
| 15 | ACHE | P22303 | acetylcholinesterase (Yt blood group) | SymMap/SwissTargetPrediction |
| 16 | ACKR3 | P25106 | C-X-C chemokine receptor type 7 | SwissTargetPrediction |
| 17 | ACP1 | P24666 | Low molecular weight phosphotyrosine protein phosphatase | SwissTargetPrediction |
| 18 | ACPP | P15309 | acid phosphatase, prostate | SymMap |
| 19 | ADAM17 | P78536 | Disintegrin and metalloproteinase domain-containing protein 17 | PharmMapper |
| 20 | ADH5 | P11766 | Alcohol dehydrogenase class-3 | PharmMapper |
| 21 | ADIPOQ | Q15848 | Adiponectin, C1Q And Collagen Domain Containing | SymMap |
| 22 | ADORA1 | P30542 | Adenosine A1 receptor | SwissTargetPrediction |
| 23 | ADORA2A | P29274 | adenosine A2a receptor | SymMap/SwissTargetPrediction |
| 24 | ADORA3 | P0DMS8 | adenosine A3 receptor | SymMap/SwissTargetPrediction |
| 25 | ADRA1A | P35348 | Alpha-1A adrenergic receptor | TCMSP/SymMap |
| 26 | ADRA1B | P35368 | Alpha-1B adrenergic receptor | TCMSP |
| 27 | ADRA2A | P08913 | adrenergic, alpha-2A-, receptor | SymMap |
| 28 | ADRA2C | P18825 | Adrenergic receptor alpha-2 | SwissTargetPrediction |
| 29 | ADRB1 | P08588 | adrenergic, beta-1-, receptor | SymMap |
| 30 | ADRB2 | P07550 | Beta-2 adrenergic receptor | TCMSP/SymMap |
| 31 | ADSL | P30566 | Adenylosuccinate lyase | PharmMapper |
| 32 | AGTR1 | P30556 | Type-1 angiotensin II receptor (by homology) | SwissTargetPrediction |
| 33 | AHCYL1 | O43865 | Adenosylhomocysteinase Like 1 | SymMap |
| 34 | AHSA1 | O95433 | Activator Of HSP90 ATPase Activity 1 | SymMap |
| 35 | AK5 | Q9Y6K8 | Adenylate kinase isoenzyme 5 | PharmMapper |
| 36 | AKR1B10 | O60218 | Aldo-keto reductase family 1 member B10 | SwissTargetPrediction |
| 37 | AKR1C1 | Q04828 | Aldo-keto reductase family 1 member C1 | SwissTargetPrediction |
| 38 | AKR1C2 | P52895 | Aldo-keto reductase family 1 member C2 | SwissTargetPrediction |
| 39 | AKR1C3 | P42330 | Aldo-keto-reductase family 1 member C3 | SwissTargetPrediction |
| 40 | AKT1 | P31749 | Serine/threonine-protein kinase AKT | SwissTargetPrediction |
| 41 | ALDH1A1 | P00352 | Retinal dehydrogenase 1 | PubChem |
| 42 | ALDH1L1 | O75891 | 10-formyltetrahydrofolate dehydrogenase | PharmMapper |
| 43 | ALDH2 | [P05091](https://www.uniprot.org/uniprot/P05091) | Aldehyde dehydrogenase, mitochondrial | TCMSP/SymMap |
| 44 | ALDH3A1 | [P30838](https://www.uniprot.org/uniprot/P30838) | Aldehyde dehydrogenase, dimeric NADP-preferring | TCMSP/SymMap |
| 45 | ALDOB | P05062 | Fructose-bisphosphate aldolase B | PharmMapper |
| 46 | ALK | Q9UM73 | ALK tyrosine kinase receptor | SwissTargetPrediction |
| 47 | ALOX12 | P18054 | Arachidonate 12-lipoxygenase | SwissTargetPrediction/PharmMapper |
| 48 | ALOX15 | P16050 | Arachidonate 15-lipoxygenase | SwissTargetPrediction/PubChem |
| 49 | ALOX5 | [P09917](https://www.uniprot.org/uniprot/P09917) | Arachidonate 5-lipoxygenase | TCMSP/SymMap/SwissTargetPrediction/STITCH |
| 50 | ALOX5AP | P20292 | 5-lipoxygenase activating protein | SwissTargetPrediction |
| 51 | AMY2A | P04746 | amylase, alpha 2A(pancreatic) | SymMap |
| 52 | ANTXR2 | P58335 | Anthrax Toxin Receptor 2 | SymMap |
| 53 | ANXA1 | P04083 | Annexin A1 | PharmMapper |
| 54 | AOC3 | Q16853 | Membrane primary amine oxidase | PharmMapper |
| 55 | APBA3 | O96018 | Amyloid beta A4 precursor protein-binding family A member 3 | PharmMapper |
| 56 | APH1A | Q96BI3 | Gamma-secretase subunit APH-1A | SwissTargetPrediction |
| 57 | APH1B | Q8WW43 | Gamma-secretase subunit APH-1B | SwissTargetPrediction |
| 58 | APP | P05067 | Beta amyloid A4 protein | SwissTargetPrediction |
| 59 | AR | P10275 | Androgen Receptor | SymMap/SwissTargetPrediction/PharmMapper |
| 60 | ARC | Q7LC44 | Activity Regulated Cytoskeleton Associated Protein | SymMap |
| 61 | ARHGAP11A | Q6P4F7 | Rho GTPase-activating protein 11A | PharmMapper |
| 62 | ARHGEF1 | Q92888 | Rho guanine nucleotide exchange factor 1 | PharmMapper |
| 63 | ARHGEF12 | Q9NZN5 | Rho guanine nucleotide exchange factor 12 | PharmMapper |
| 64 | ARL2 | P36404 | ADP-ribosylation factor-like protein 2 | PharmMapper |
| 65 | ATIC | P31939 | Bifunctional purine biosynthesis protein PURH | PharmMapper |
| 66 | ATM | Q13315 | ATM Serine/threonine Kinase | SymMap |
| 67 | ATP12A | P54707 | Potassium-transporting ATPase alpha chain 2 | SwissTargetPrediction |
| 68 | ATP5B | P06576 | ATP synthase F1 subunit beta | SymMap |
| 69 | AURKB | Q96GD4 | Serine/threonine-protein kinase Aurora-B | SwissTargetPrediction |
| 70 | AVPR1A | P37288 | Vasopressin V1a receptor | SwissTargetPrediction |
| 71 | AVPR2 | P30518 | Vasopressin V2 receptor | SwissTargetPrediction |
| 72 | AZGP1 | P25311 | Zinc-alpha-2-glycoprotein | PharmMapper |
| 73 | B2M | P61769 | Beta-2-microglobulin | PharmMapper |
| 74 | BACE1 | P56817 | Beta-secretase 1 | SwissTargetPrediction |
| 75 | BACE2 | Q9Y5Z0 | Beta secretase 2 | SwissTargetPrediction |
| 76 | BAX | Q07812 | BCL2 Associated X, Apoptosis Regulator | TCMSP/SymMap |
| 77 | BCAT1 | P54687 | Branched-chain-amino-acid aminotransferase, cytosolic | PharmMapper |
| 78 | BCHE | P06276 | butyrylcholinesterase | SymMap/SwissTargetPrediction |
| 79 | BCL2 | P10415 | Apoptosis regulator Bcl-2 | TCMSP/SymMap/SwissTargetPrediction |
| 80 | BCL2L1 | Q07817 | Apoptosis regulator Bcl-X | SwissTargetPrediction |
| 81 | BDNF | P23560 | Brain Derived Neurotrophic Factor | SymMap |
| 82 | BLMH | Q13867 | Bleomycin hydrolase | PharmMapper |
| 83 | BLVRA | P53004 | Biliverdin reductase A | PharmMapper |
| 84 | BMP1 | P13497 | Bone morphogenetic protein 1 | SwissTargetPrediction |
| 85 | BRD2 | P25440 | Bromodomain-containing protein 2 | SwissTargetPrediction |
| 86 | BRD3 | Q15059 | Bromodomain-containing protein 3 | SwissTargetPrediction |
| 87 | BRD4 | O60885 | Bromodomain-containing protein 4 | SwissTargetPrediction |
| 88 | BRDT | Q58F21 | Bromodomain testis-specific protein | PharmMapper |
| 89 | C1QBP | Q07021 | Complement component 1 Q subcomponent-binding protein, mitochondrial | PharmMapper |
| 90 | C1R | [P00736](https://www.uniprot.org/uniprot/P00736) | Complement C1r subcomponent | TCMSP/SymMap/PharmMapper |
| 91 | C5 | P01031 | Complement C5 | PharmMapper |
| 92 | C5AR1 | P21730 | Complement C5a Receptor 1 | SwissTargetPrediction/SymMap |
| 93 | C8A | P07357 | Complement component C8 alpha chain | PharmMapper |
| 94 | CA1 | P00915 | Carbonic anhydrase I | SwissTargetPrediction |
| 95 | CA14 | Q9ULX7 | Carbonic anhydrase XIV | SwissTargetPrediction |
| 96 | CA2 | P00918 | Carbonic anhydrase II | SwissTargetPrediction |
| 97 | CACNA2D1 | P54289 | Voltage-gated calcium channel alpha2/delta subunit 1 | SwissTargetPrediction |
| 98 | CALM3 | P0DP25 | Calmodulin 3 | SymMap |
| 99 | CALML3 | P27482 | Calmodulin-like protein 3 | PharmMapper |
| 100 | CAMK2D | Q13557 | Calcium/calmodulin-dependent protein kinase type II delta chain | PharmMapper |
| 101 | CAMK4 | Q16566 | Calcium/calmodulin-dependent protein kinase type IV | PharmMapper |
| 102 | CANT1 | Q8WVQ1 | Soluble calcium-activated nucleotidase 1 | PharmMapper |
| 103 | CANX | P27824 | Calnexin | PharmMapper |
| 104 | CAPN13 | Q6MZZ7 | Calpain-13 | PharmMapper |
| 105 | CAPN9 | O14815 | Calpain-9 | PharmMapper |
| 106 | CASP3 | [P42574](https://www.uniprot.org/uniprot/P42574) | Caspase-3 | TCMSP/SymMap |
| 107 | CASP8 | [Q14790](https://www.uniprot.org/uniprot/Q14790) | Caspase-8 | TCMSP |
| 108 | CASP9 | P55211 | Caspase 9 | SymMap/TCMSP |
| 109 | CASR | P41180 | Calcium sensing receptor | SwissTargetPrediction |
| 110 | CAT | P04040 | Catalase | SymMap |
| 111 | CCK | P06307 | Cholecystokinin | SymMap |
| 112 | CCKBR | P32239 | Cholecystokinin B receptor | SwissTargetPrediction |
| 113 | CCL2 | P13500 | C-C motif chemokine ligand 2 | SymMap |
| 114 | CCND1 | [P24385](https://www.uniprot.org/uniprot/P24385) | G1/S-specific cyclin-D1 | TCMSP/SymMap |
| 115 | CCND1 | P24385 | Cyclin-dependent kinase 4/cyclin D1 | SwissTargetPrediction |
| 116 | CCNE1 | P24864 | G1/S-specific cyclin-E1 | SwissTargetPrediction |
| 117 | CCNE2 | O96020 | G1/S-specific cyclin-E2 | SwissTargetPrediction |
| 118 | CCNT1 | O60563 | Cyclin T1 | SwissTargetPrediction |
| 119 | CCR1 | P32246 | C-C chemokine receptor type 1 | SwissTargetPrediction |
| 120 | CCR2 | P41597 | C-C chemokine receptor type 2 | SwissTargetPrediction |
| 121 | CCR3 | P51677 | C-C chemokine receptor type 3 | SwissTargetPrediction |
| 122 | CCR4 | P51679 | C-C chemokine receptor type 4 | SwissTargetPrediction |
| 123 | CCR5 | P51681 | C-C chemokine receptor type 5 | SwissTargetPrediction |
| 124 | CD1B | P29016 | T-cell surface glycoprotein CD1b | PharmMapper |
| 125 | CD40LG | P29965 | CD40 Ligand | SymMap |
| 126 | CDC25A | P30304 | Dual specificity phosphatase Cdc25A | SwissTargetPrediction |
| 127 | CDC25B | P30305 | Dual specificity phosphatase Cdc25B | SwissTargetPrediction |
| 128 | CDC7 | O00311 | CDC7/DBF4 (Cell division cycle 7-related protein kinase/Activator of S phase kinase) | SwissTargetPrediction |
| 129 | CDK1 | P06493 | Cyclin-dependent kinase 1 | SwissTargetPrediction/SymMap |
| 130 | CDK2 | P24941 | cyclin dependent kinase 2 | SymMap/SwissTargetPrediction |
| 131 | CDK4 | [P11802](https://www.uniprot.org/uniprot/P11802) | Cell division protein kinase 4 | TCMSP/SymMap/SwissTargetPrediction |
| 132 | CDK6 | Q00534 | Cyclin-dependent kinase 6 | SwissTargetPrediction |
| 133 | CDKN1A | P38936 | Cyclin Dependent Kinase Inhibitor 1A | SymMap |
| 134 | CELF4 | Q9BZC1 | CUG-BP- and ETR-3-like factor 4 | PharmMapper |
| 135 | CENPB | P07199 | Major centromere autoantigen B | PharmMapper |
| 136 | CES1 | P23141 | Acyl coenzyme A:cholesterol acyltransferase | SwissTargetPrediction |
| 137 | CES2 | O00748 | Carboxylesterase 2 | SwissTargetPrediction |
| 138 | CETP | [P11597](https://www.uniprot.org/uniprot/P11597) | Cholesteryl ester transfer protein | TCMSP/SymMap |
| 139 | CHRM1 | [P11229](https://www.uniprot.org/uniprot/P11229) | Muscarinic acetylcholine receptor M1 | TCMSP/SymMapSwissTarget/Prediction |
| 140 | CHRM2 | P08172 | Muscarinic acetylcholine receptor M2 | TCMSP/SwissTargetPrediction |
| 141 | CHRM3 | [P20309](https://www.uniprot.org/uniprot/P20309) | Muscarinic acetylcholine receptor M3 | TCMSP |
| 142 | CHRM4 | [P08173](https://www.uniprot.org/uniprot/P08173) | Muscarinic acetylcholine receptor M4 | TCMSP/SwissTargetPrediction |
| 143 | CHRNA2 | Q15822 | Neuronal acetylcholine receptor subunit alpha-2 | TCMSP |
| 144 | CHRNA4 | P43681 | Cholinergic Receptor Nicotinic Alpha 4 Subunit | SymMap |
| 145 | CHRNA7 | P36544 | Neuronal acetylcholine receptor protein, alpha-7 chain | TCMSP |
| 146 | CITED1 | Q99966 | Cbp/p300 Interacting Transactivator With Glu/Asp Rich Carboxy-terminal Domain 1 | SymMap |
| 147 | CITED2 | Q99967 | Cbp/p300-interacting transactivator 2 | PharmMapper |
| 148 | CKM | P06732 | Creatine kinase M-type | PharmMapper |
| 149 | CLCN2 | P51788 | Chloride channel protein 2 | PubChem |
| 150 | CLPP | Q16740 | Putative ATP-dependent Clp protease proteolytic subunit, mitochondrial | PharmMapper |
| 151 | CMA1 | P23946 | Chymase | SwissTargetPrediction |
| 152 | CNP | P09543 | 2,3-cyclic-nucleotide 3-phosphodiesterase | PharmMapper |
| 153 | CNR1 | P21554 | Cannabinoid receptor 1 | SwissTargetPrediction |
| 154 | CNR2 | P34972 | Cannabinoid receptor 2 | SwissTargetPrediction |
| 155 | COL1A1 | P02452 | Collagen Type I Alpha 1 Chain | SymMap |
| 156 | COL1A2 | [P08123](https://www.uniprot.org/uniprot/P08123) | Collagen alpha-2(I) chain | TCMSP/SymMap |
| 157 | COL4A2 | P08572 | Collagen Type IV Alpha 2 Chain | SymMap |
| 158 | COL7A1 | Q02388 | Collagen Type VII Alpha 1 Chain | SymMap |
| 159 | CPSF3 | Q9UKF6 | Cleavage and polyadenylation specificity factor subunit 3 | PharmMapper |
| 160 | CRHR1 | P34998 | Corticotropin releasing factor receptor 1 | SwissTargetPrediction |
| 161 | CRLF1 | O75462 | Cytokine Receptor Like Factor 1 | SymMap |
| 162 | crn-7 | P34387 | Cell-death-related Nuclease 7 | SymMap |
| 163 | CRP | P02741 | C-reactive protein | SymMap |
| 164 | CRYZ | Q08257 | Quinone oxidoreductase | PharmMapper |
| 165 | CSDE1 | O75534 | Cold shock domain-containing protein E1 | PharmMapper |
| 166 | CSF1R | P07333 | Macrophage colony stimulating factor receptor | SwissTargetPrediction |
| 167 | CSF2RB | P32927 | Cytokine receptor common subunit beta | PharmMapper |
| 168 | CTGF | P29279 | Connective Tissue Growth Factor | SymMap |
| 169 | CTNNB1 | P35222 | Catenin Beta 1 | SymMap |
| 170 | CTRC | Q99895 | Chymotrypsin C | SwissTargetPrediction |
| 171 | CTSB | P07858 | Cathepsin (B and K) | SwissTargetPrediction |
| 172 | CTSD | P07339 | Cathepsin D | SymMap |
| 173 | CTSG | P08311 | Cathepsin G | SwissTargetPrediction |
| 174 | CTSK | P43235 | Cathepsin K | SwissTargetPrediction |
| 175 | CTSL | P07711 | Cathepsin L | SwissTargetPrediction |
| 176 | CTSS | P25774 | Cathepsin S | SwissTargetPrediction |
| 177 | CUL1 | Q13616 | Cullin-1 | PharmMapper |
| 178 | CUL5 | Q93034 | Cullin-5 | PharmMapper |
| 179 | CUTA | O60888 | CutA Divalent Cation Tolerance Homolog | SymMap |
| 180 | CXCR1 | P25024 | Interleukin-8 receptor A | SwissTargetPrediction |
| 181 | CXCR3 | P49682 | C-X-C chemokine receptor type 3 | SwissTargetPrediction |
| 182 | CYP11B1 | P15538 | Cytochrome P450 11B1 | SwissTargetPrediction |
| 183 | CYP11B2 | P19099 | Cytochrome P450 11B2 | SwissTargetPrediction |
| 184 | CYP17A1 | P05093 | Cytochrome P450 17A1 | SwissTargetPrediction |
| 185 | CYP19A1 | P11511 | Cytochrome P450 19A1 | SwissTargetPrediction/PubChem |
| 186 | CYP1A1 | P04798 | cytochrome P450 family 1 subfamily A member 1 | SymMap |
| 187 | CYP1A2 | P05177 | cytochrome P450 family 1 subfamily A member 2 | SymMap |
| 188 | CYP24A1 | Q07973 | Cytochrome P450 24A1 | SwissTargetPrediction |
| 189 | CYP26A1 | O43174 | Cytochrome P450 26A1 | SwissTargetPrediction |
| 190 | CYP26B1 | Q9NR63 | Cytochrome P450 26B1 | SwissTargetPrediction |
| 191 | CYP2C19 | P33261 | Cytochrome P450 2C19 | SwissTargetPrediction |
| 192 | CYP2C9 | P11712 | Cytochrome P450 2C9 | SwissTargetPrediction |
| 193 | CYP2E1 | P05181 | cytochrome P450 family 2 subfamily E member 1 | SymMap/PharmMapper |
| 194 | CYP3A4 | P08684 | Cytochrome P450 3A4 | SwissTargetPrediction |
| 195 | CYP51A1 | Q16850 | Cytochrome P450 51 (by homology) | SwissTargetPrediction |
| 196 | DAGLA | Q9Y4D2 | Sn1-specific diacylglycerol lipase alpha | SwissTargetPrediction |
| 197 | DAPK3 | O43293 | Death-associated protein kinase 3 | PharmMapper |
| 198 | DAPP1 | Q9UN19 | Dual adapter for phosphotyrosine and 3-phosphotyrosine and 3- phosphoinositide | PharmMapper |
| 199 | DBT | P11182 | Dihydrolipoamide Branched Chain Transacylase E2 | SymMap |
| 200 | DDIT3 | P35638 | DNA Damage Inducible Transcript 3 | SymMap |
| 201 | DGAT1 | O75907 | Diacylglycerol O-acyltransferase 1 | SwissTargetPrediction |
| 202 | DHCR7 | Q9UBM7 | 7-dehydrocholesterol Reductase | SymMap/SwissTargetPrediction |
| 203 | DHFR | P00374 | Dihydrofolate reductase | SwissTargetPrediction |
| 204 | DHRS11 | Q6UWP2 | Dehydrogenase/reductase SDR family member 11 | PharmMapper |
| 205 | DMC1 | Q14565 | Meiotic recombination protein DMC1/LIM15 homolog | PharmMapper |
| 206 | DNPEP | Q9ULA0 | aspartyl aminopeptidase | SymMap |
| 207 | DNTT | P04053 | Terminal deoxynucleotidyltransferase | SwissTargetPrediction |
| 208 | DPP4 | P27487 | Dipeptidyl peptidase 4 | PubChem |
| 209 | DRAP1 | Q14919 | Dr1-associated corepressor | PharmMapper |
| 210 | DRD1 | [P21728](https://www.uniprot.org/uniprot/P21728) | Dopamine D1 receptor | TCMSP |
| 211 | DRD2 | P14416 | Dopamine Receptor D2 | SymMap/SwissTargetPrediction |
| 212 | E2F1 | Q01094 | E2F Transcription Factor 1 | SymMap/PharmMapper |
| 213 | EBP | Q15125 | Anti-estrogen binding site (AEBS) | SwissTargetPrediction |
| 214 | EDN1 | P05305 | Endothelin 1 | SymMap |
| 215 | EDNRA | P25101 | Endothelin receptor ET-A (by homology) | SwissTargetPrediction |
| 216 | EGF | [P01133](https://www.uniprot.org/uniprot/P01133) | Pro-epidermal growth factor | TCMSP/SymMap |
| 217 | EGR1 | P18146 | Early Growth Response 1 | SymMap |
| 218 | EIF4A1 | P60842 | Eukaryotic initiation factor 4A-I | PharmMapper |
| 219 | ENPEP | Q07075 | glutamyl aminopeptidase | SymMap |
| 220 | ENPP2 | Q13822 | Autotaxin | SwissTargetPrediction |
| 221 | ENPP7 | Q6UWV6 | Ectonucleotide Pyrophosphatase/phosphodiesterase 7 | SymMap |
| 222 | EP300 | Q09472 | Histone acetyltransferase p300 | PharmMapper |
| 223 | EPAS1 | Q99814 | Endothelial PAS domain-containing protein 1 | SwissTargetPrediction |
| 224 | EPHB4 | P54760 | Ephrin receptor | SwissTargetPrediction |
| 225 | EPHX1 | P07099 | Epoxide hydrolase 1 | SwissTargetPrediction |
| 226 | ERBB2 | P04626 | Erb-b2 Receptor Tyrosine Kinase 2 | SymMap |
| 227 | ERI1 | Q8IV48 | 3-5 exoribonuclease 1 | PharmMapper |
| 228 | ERN1 | O75460 | Serine/threonine-protein kinase/endoribonuclease IRE1 | SwissTargetPrediction |
| 229 | ESR1 | P03372 | Estrogen Receptor 1 | SymMap/SwissTargetPrediction/PharmMapper |
| 230 | ESR2 | Q92731 | Estrogen receptor beta | SwissTargetPrediction |
| 231 | EZH2 | Q15910 | EZH2/SUZ12/EED/RBBP7/RBBP4 | SwissTargetPrediction |
| 232 | F10 | P00742 | Thrombin and coagulation factor X | SwissTargetPrediction |
| 233 | F11 | P03951 | Coagulation factor XI | SwissTargetPrediction |
| 234 | F13A1 | P00488 | Coagulation factor XIII A chain | PharmMapper |
| 235 | F2 | P00734 | Thrombin | SwissTargetPrediction |
| 236 | F2R | P25116 | Proteinase-activated receptor 1 | SwissTargetPrediction |
| 237 | FAAH | O00519 | Anandamide amidohydrolase | SwissTargetPrediction |
| 238 | FABP1 | P07148 | Fatty Acid Binding Protein 1 | SymMap/SwissTargetPrediction |
| 239 | FABP2 | P12104 | Fatty acid binding protein intestinal | SwissTargetPrediction |
| 240 | FABP3 | P05413 | Fatty acid binding protein muscle | SwissTargetPrediction |
| 241 | FABP4 | P15090 | Fatty acid binding protein adipocyte | SwissTargetPrediction/PubChem |
| 242 | FABP5 | Q01469 | Fatty acid binding protein epidermal | SwissTargetPrediction |
| 243 | FAP | Q12884 | Prolyl endopeptidase FAP | PharmMapper |
| 244 | FASN | P49327 | Fatty acid synthase | SwissTargetPrediction |
| 245 | FDFT1 | P37268 | Squalene synthetase (by homology) | SwissTargetPrediction |
| 246 | FES | P07332 | Proto-oncogene tyrosine-protein kinase Fes/Fps | PharmMapper |
| 247 | FFAR1 | O14842 | Free fatty acid receptor 1 | SwissTargetPrediction |
| 248 | FFAR4 | Q5NUL3 | G-protein coupled receptor 120 | SwissTargetPrediction |
| 249 | FKBP4 | Q02790 | FK506-binding protein 4 | PharmMapper |
| 250 | FNTA | P49354 | Protein farnesyltransferase | SwissTargetPrediction |
| 251 | FNTB | P49356 | Protein farnesyltransferase subunit beta | SwissTargetPrediction |
| 252 | FOS | P01100 | Fos Proto-oncogene, AP-1 Transcription Factor Subunit | SymMap |
| 253 | FOXM1 | Q08050 | Forkhead box protein M1 | PharmMapper |
| 254 | FSHB | P01225 | Subunitfollicle stimulating hormone subunit beta | SymMap |
| 255 | G6PD | [P11413](https://www.uniprot.org/uniprot/P11413) | Glucose-6-phosphate 1-dehydrogenase | TCMSPSymMapSwissTargetPrediction |
| 256 | GABBR1 | Q9UBS5 | GABA-B receptor (by homology) | SwissTargetPrediction |
| 257 | GABRA1 | P14867 | Gamma-aminobutyric acid receptor subunit alpha-1 | TCMSP/SwissTargetPrediction |
| 258 | GABRA2 | P47869 | Gamma-aminobutyric-acid receptor alpha-2 subunit | TCMSP/SymMap/SwissTargetPrediction |
| 259 | GABRA3 | P34903 | Gamma-aminobutyric-acid receptor alpha-3 subunit | TCMSP/SwissTargetPrediction |
| 260 | GABRA5 | P31644 | Gamma-aminobutyric-acid receptor alpha-5 subunit | TCMSP/SwissTargetPrediction |
| 261 | GABRB3 | P28472 | GABA-A receptor beta-3 | SwissTargetPrediction |
| 262 | GABRG2 | P18507 | GABA-A receptor gamma-2 | SwissTargetPrediction |
| 263 | GAD2 | Q05329 | Glutamate decarboxylase 2 | PharmMapper |
| 264 | GALK1 | P51570 | Galactokinase | PharmMapper |
| 265 | GALM | Q96C23 | Aldose 1-epimerase | PharmMapper |
| 266 | GAP43 | P17677 | Growth Associated Protein 43 | SymMap |
| 267 | GBP1 | P32455 | Interferon-induced guanylate-binding protein 1 | PharmMapper |
| 268 | GC | P02774 | Vitamin D-binding protein | SwissTargetPrediction |
| 269 | GCDH | Q92947 | Glutaryl-CoA dehydrogenase, mitochondrial | PharmMapper |
| 270 | GCG | P01275 | Glucagon | SymMap |
| 271 | GCGR | P47871 | Glucagon receptor | SwissTargetPrediction |
| 272 | GCH1 | P30793 | GTP cyclohydrolase I | PharmMapper |
| 273 | GCK | P35557 | Hexokinase type IV | SwissTargetPrediction |
| 274 | GFER | P55789 | FAD-linked sulfhydryl oxidase ALR | PubChem |
| 275 | GLB1 | [P16278](https://www.uniprot.org/uniprot/P16278) | Beta-galactosidase | TCMSP/SymMap |
| 276 | GLRA1 | P23415 | Glycine receptor subunit alpha-1 | SwissTargetPrediction |
| 277 | GLRB | P48167 | Glycine receptor subunit beta | PharmMapper |
| 278 | GPR119 | Q8TDV5 | Glucose-dependent insulinotropic receptor | SwissTargetPrediction |
| 279 | GPT | P24298 | glutamic--pyruvic transaminase | SymMap |
| 280 | GPT2 | Q8TD30 | Glutamic--pyruvic Transaminase 2 | SymMap |
| 281 | GRIA2 | P42262 | glutamate ionotropic receptor AMPA type subunit 2 | SymMap/SwissTargetPrediction |
| 282 | GRIN1 | Q05586 | Glutamate NMDA receptor; GRIN1/GRIN2B | SwissTargetPrediction |
| 283 | GRIN2B | Q13224 | Glutamate [NMDA] receptor subunit epsilon 2 | SwissTargetPrediction |
| 284 | GRM1 | Q13255 | Metabotropic glutamate receptor 1 | SwissTargetPrediction |
| 285 | GRM2 | Q14416 | Metabotropic glutamate receptor 2 | SwissTargetPrediction |
| 286 | GRM5 | P41594 | Metabotropic glutamate receptor 5 (by homology) | SwissTargetPrediction |
| 287 | GSK3B | P49841 | Glycogen synthase kinase-3 beta | SwissTargetPrediction/PubChem |
| 288 | GSTT2 | P0CG29 | Glutathione S-transferase theta-2 | PharmMapper |
| 289 | GUCY2F | P51841 | Guanylate Cyclase 2F, Retinal | SymMap |
| 290 | GUSBP1 | Q15486 | Glucuronidase, Beta Pseudogene 1 | SymMap |
| 291 | GYS1 | P13807 | Muscle glycogen synthase | SwissTargetPrediction |
| 292 | HASPIN | Q8TF76 | Serine/threonine-protein kinase haspin | PharmMapper |
| 293 | HBB | P68871 | Hemoglobin subunit beta | PharmMapper |
| 294 | HBEGF | Q99075 | Proheparin-binding EGF-like growth factor | PharmMapper |
| 295 | HCRTR1 | O43613 | Orexin receptor 1 | SwissTargetPrediction |
| 296 | HCRTR2 | O43614 | Orexin receptor 2 | SwissTargetPrediction |
| 297 | HDAC6 | Q9UBN7 | Histone deacetylase 6 | SwissTargetPrediction |
| 298 | HERC5 | [Q9UII4](https://www.uniprot.org/uniprot/Q9UII4) | Probable E3 ubiquitin-protein ligase HERC5 | TCMSP/SymMap |
| 299 | HGD | Q93099 | Homogentisate 1,2-dioxygenase | PharmMapper |
| 300 | HIF1A | Q16665 | Hypoxia-inducible factor 1 alpha | SwissTargetPrediction |
| 301 | HK2 | P52789 | Hexokinase-2 | PharmMapper |
| 302 | HLA-DPB1 | P04440 | HLA class II histocompatibility antigen, DRB1-1 beta chain | PharmMapper |
| 303 | HLA-E | P13747 | HLA class I histocompatibility antigen, alpha chain E | PharmMapper |
| 304 | HMBOX1 | Q6NT76 | Homeobox-containing protein 1 | PharmMapper |
| 305 | HMGCR | P04035 | 3-hydroxy-3-methylglutaryl-CoA reductase | SymMap/SwissTargetPrediction |
| 306 | HMGCS1 | Q01581 | Hydroxymethylglutaryl-CoA synthase, cytoplasmic | PharmMapper |
| 307 | HMOX1 | P09601 | Heme Oxygenase 1 | SymMap/PharmMapper |
| 308 | HNF4A | P41235 | Hepatocyte nuclear factor 4-alpha | SwissTargetPrediction |
| 309 | HNF4G | Q14541 | Hepatocyte nuclear factor 4-gamma | PharmMapper |
| 310 | HNMT | P50135 | Histamine N-methyltransferase | PharmMapper |
| 311 | HNRNPR | O43390 | Heterogeneous nuclear ribonucleoprotein R | PharmMapper |
| 312 | HOMEZ | Q8IX15 | Homeobox and leucine zipper protein Homez | PharmMapper |
| 313 | HOXB13 | Q92826 | Homeobox protein Hox-B13 | PharmMapper |
| 314 | HPGD | P15428 | 15-hydroxyprostaglandin dehydrogenase [NAD+] | SwissTargetPrediction/PubChem |
| 315 | HPGDS | O60760 | Hematopoietic prostaglandin D synthase | SwissTargetPrediction |
| 316 | HRH2 | P25021 | Histamine H2 receptor | SwissTargetPrediction |
| 317 | HRH3 | Q9Y5N1 | Histamine H3 receptor | SwissTargetPrediction |
| 318 | HRH4 | Q9H3N8 | Histamine H4 receptor | SwissTargetPrediction |
| 319 | HSD11B1 | P28845 | 11-beta-hydroxysteroid dehydrogenase 1 | SwissTargetPrediction/PharmMapper |
| 320 | HSD11B2 | P80365 | 11-beta-hydroxysteroid dehydrogenase 2 | SwissTargetPrediction |
| 321 | HSD17B1 | P14061 | Estradiol 17-beta-dehydrogenase 1 | PharmMapper |
| 322 | HSD17B2 | P37059 | Estradiol 17-beta-dehydrogenase 2 | SwissTargetPrediction |
| 323 | HSD17B4 | P51659 | Peroxisomal multifunctional enzyme type 2 | PharmMapper |
| 324 | HSD3B2 | P26439 | 3-beta-hydroxysteroid dehydrogenase/delta 5-->4-isomerase type II | SwissTargetPrediction |
| 325 | HTR1A | P08908 | Serotonin 1a (5-HT1a) receptor | SwissTargetPrediction |
| 326 | HTR2A | [P28223](https://www.uniprot.org/uniprot/P28223) | 5-hydroxytryptamine 2A receptor | TCMSP |
| 327 | HTR2B | P41595 | Serotonin 2b (5-HT2b) receptor | SwissTargetPrediction |
| 328 | ICAM1 | P05362 | intercellular adhesion molecule 1 | SymMap/SwissTargetPrediction |
| 329 | ICMT | O60725 | Isoprenylcysteine carboxyl methyltransferase | SwissTargetPrediction |
| 330 | IDE | P14735 | Insulin-degrading enzyme | PharmMapper |
| 331 | IDH1 | O75874 | Isocitrate dehydrogenase [NADP] cytoplasmic | SwissTargetPrediction |
| 332 | IDO1 | P14902 | Indoleamine 2,3-dioxygenase | SwissTargetPrediction |
| 333 | IER5 | Q5VY09 | Immediate Early Response 5 | SymMap |
| 334 | IFNAR2 | P48551 | interferon alpha and beta receptor subunit 2 | SymMap |
| 335 | IFNB1 | P01574 | interferon beta 1 | SymMap |
| 336 | IGF1R | P08069 | Insulin-like growth factor I receptor | SwissTargetPrediction |
| 337 | IGHG1 | P01857 | immunoglobulin heavy constant gamma 1 | SymMap |
| 338 | IGKC | P01834 | Ig kappa chain C region | PharmMapper |
| 339 | IKBKB | O14920 | Inhibitor of nuclear factor kappa B kinase beta subunit | SwissTargetPrediction |
| 340 | IL10 | P22301 | Interleukin 10 | SymMap |
| 341 | IL10RA | Q13651 | Interleukin-10 receptor alpha chain | PharmMapper |
| 342 | IL1A | P01583 | interleukin 1 alpha | SymMap |
| 343 | IL1B | P01584 | interleukin 1 beta | SymMap/SwissTargetPrediction |
| 344 | IL6 | P05231 | Interleukin 6 | SymMap |
| 345 | IL6ST | P40189 | Interleukin-6 receptor subunit beta | SwissTargetPrediction |
| 346 | INCENP | Q9NQS7 | Inner centromere protein | SwissTargetPrediction |
| 347 | INS | P01308 | Insulin | SymMap |
| 348 | IQUB | Q8NA54 | IQ and ubiquitin-like domain-containing protein | PharmMapper |
| 349 | IRF3 | Q14653 | Interferon Regulatory Factor 3 | SymMap |
| 350 | ITGAL | P20701 | Intercellular adhesion molecule (ICAM-1), Integrin alpha-L/beta-2 | SwissTargetPrediction |
| 351 | ITGB2 | P05107 | Integrin beta-2 | SwissTargetPrediction |
| 352 | ITGB3 | P05106 | Integrin Subunit Beta 3 | SymMap |
| 353 | ITK | Q08881 | Tyrosine-protein kinase ITK/TSK | SwissTargetPrediction |
| 354 | ITPKC | Q96DU7 | Inositol-trisphosphate 3-kinase C | PharmMapper |
| 355 | JAK2 | O60674 | Tyrosine-protein kinase JAK2 | SwissTargetPrediction |
| 356 | JAK3 | P52333 | Tyrosine-protein kinase JAK3 | SwissTargetPrediction |
| 357 | JUN | P05412 | Transcription factor AP-1 | TCMSP/SymMap |
| 358 | KAT2A | Q92830 | Histone acetyltransferase KAT2A | PubChem |
| 359 | KCNA5 | P22460 | Voltage-gated potassium channel subunit Kv1.5 | SwissTargetPrediction |
| 360 | KCNH2 | [Q12809](https://www.uniprot.org/uniprot/Q12809) | Potassium voltage-gated channel subfamily H member 2 | TCMSP/SymMapSwissTargetPrediction |
| 361 | KCNJ11 | Q14654 | Potassium Voltage-gated Channel Subfamily J Member 11 | SymMap |
| 362 | KCNJ4 | P48050 | Inward rectifier potassium channel 4 | PubChem |
| 363 | KCNK10 | [P57789](https://www.uniprot.org/uniprot/P57789) | Potassium channel subfamily K member 10 | TCMSP/SymMap/PubChem |
| 364 | KCNK18 | Q7Z418 | Potassium channel subfamily K member 18 | PubChem |
| 365 | KCNK2 | [O95069](https://www.uniprot.org/uniprot/O95069) | Potassium channel subfamily K member 2 | TCMSP/SymMap/PubChem |
| 366 | KCNK3 | O14649 | Potassium channel subfamily K member 3 | PubChem |
| 367 | KCNK4 | Q9NYG8 | Potassium channel subfamily K member 4 | PubChem |
| 368 | KDM2A | Q9Y2K7 | Lysine-specific demethylase 2A | SwissTargetPrediction |
| 369 | KDM5C | P41229 | Lysine-specific demethylase 5C | SwissTargetPrediction |
| 370 | KDR | P35968 | Kinase Insert Domain Receptor | SymMap/SwissTargetPrediction |
| 371 | KEAP1 | Q14145 | Kelch-like ECH-associated protein 1 | SwissTargetPrediction |
| 372 | KIF11 | P52732 | Kinesin-like protein 1 | SwissTargetPrediction |
| 373 | KIT | P10721 | Stem cell growth factor receptor | SwissTargetPrediction |
| 374 | KLF10 | Q13118 | Krueppel-like factor 10 | PharmMapper |
| 375 | LCK | P06239 | Tyrosine-protein kinase LCK | SwissTargetPrediction |
| 376 | LCT | P09848 | Lactase | SymMap |
| 377 | LHB | P01229 | Luteinizing Hormone Beta Polypeptide | SymMap |
| 378 | LIG1 | P18858 | DNA ligase 1 | PharmMapper |
| 379 | LIPE | Q05469 | Hormone sensitive lipase | SwissTargetPrediction |
| 380 | LPL | P06858 | Lipoprotein Lipase | SymMap |
| 381 | LSS | P48449 | Lanosterol synthase | PharmMapper |
| 382 | LTB4R | Q15722 | Leukotriene B4 receptor 1 | SwissTargetPrediction |
| 383 | MAN1B1 | Q9UKM7 | Endoplasmic reticulum mannosyl-oligosaccharide 1,2-alpha-mannosidase | PharmMapper |
| 384 | MAOB | P27338 | monoamine oxidase B | SymMap/PharmMapper |
| 385 | MAP2 | P11137 | Microtubule-associated protein 2 | TCMSP/SymMap |
| 386 | MAP3K14 | Q99558 | Mitogen-activated protein kinase kinase kinase 14 | SwissTargetPrediction |
| 387 | MAP3K20 | Q9NYL2 | Mixed lineage kinase 7 | SwissTargetPrediction |
| 388 | MAPK1 | [P28482](https://www.uniprot.org/uniprot/P28482) | Mitogen-activated protein kinase 1 | TCMSP/SymMap |
| 389 | MAPK10 | P53779 | c-Jun N-terminal kinase 3 | SwissTargetPrediction |
| 390 | MAPK14 | Q16539 | MAP kinase p38 alpha | SwissTargetPrediction |
| 391 | MAPK3 | P27361 | Mitogen-activated Protein Kinase 3 | SymMap/SwissTargetPrediction |
| 392 | MAPK8 | P45983 | c-Jun N-terminal kinase 1 | SwissTargetPrediction |
| 393 | MAPK9 | P45984 | c-Jun N-terminal kinase 2 | SwissTargetPrediction/PharmMapper |
| 394 | MAPT | P10636 | Microtubule-associated protein tau | PubChem |
| 395 | MAST3 | O60307 | Microtubule-associated serine/threonine-protein kinase 3 | SwissTargetPrediction |
| 396 | MCL1 | Q07820 | Induced myeloid leukemia cell differentiation protein Mcl-1 | SwissTargetPrediction |
| 397 | MDM2 | Q00987 | p53-binding protein Mdm-2 | SwissTargetPrediction |
| 398 | MDM4 | O15151 | Protein Mdm4 | SwissTargetPrediction |
| 399 | MEF2A | Q02078 | Myocyte Enhancer Factor 2A | SymMap |
| 400 | MEF2D | Q14814 | Myocyte Enhancer Factor 2D | SymMap |
| 401 | MERTK | Q12866 | Proto-oncogene tyrosine-protein kinase MER | SwissTargetPrediction |
| 402 | MET | P08581 | Hepatocyte growth factor receptor | SwissTargetPrediction |
| 403 | METAP1 | P53582 | Methionine aminopeptidase 1 | SwissTargetPrediction |
| 404 | METAP2 | P50579 | Methionine aminopeptidase 2 | SwissTargetPrediction |
| 405 | METTL16 | Q86W50 | Putative methyltransferase METT10D | PharmMapper |
| 406 | MGLL | Q99685 | Monoglyceride lipase | SwissTargetPrediction |
| 407 | MKI67 | P46013 | Antigen KI-67 | PharmMapper |
| 408 | MME | P08473 | membrane metalloendopeptidase | SymMap |
| 409 | MMP1 | P03956 | Matrix metalloproteinase 1 | SwissTargetPrediction |
| 410 | MMP13 | P45452 | Matrix metalloproteinase 13 | SwissTargetPrediction |
| 411 | MMP14 | P50281 | Matrix metalloproteinase 14 | SwissTargetPrediction |
| 412 | MMP2 | P08253 | Matrix metalloproteinase 2 | SwissTargetPrediction/PharmMapper |
| 413 | MMP3 | P08254 | Matrix metalloproteinase 3 | SwissTargetPrediction |
| 414 | MMP8 | P22894 | Matrix metalloproteinase 8 | SwissTargetPrediction |
| 415 | MMP9 | P14780 | Matrix metalloproteinase 9 | SwissTargetPrediction |
| 416 | MMUT | P22033 | Methylmalonyl-CoA mutase, mitochondrial | PharmMapper |
| 417 | MPO | P05164 | Myeloperoxidase | SymMap/PharmMapper |
| 418 | MS4A1 | P11836 | Membrane Spanning 4-domains A1 | SymMap |
| 419 | MSH2 | P43246 | DNA mismatch repair protein Msh2 | PharmMapper |
| 420 | MTNR1A | P48039 | Melatonin receptor 1A | SwissTargetPrediction |
| 421 | MTNR1B | P49286 | Melatonin receptor 1B | SwissTargetPrediction |
| 422 | MTOR | P42345 | Serine/threonine-protein kinase mTOR | SwissTargetPrediction |
| 423 | MUC1 | P15941 | Mucin-1 | PharmMapper |
| 424 | MYC | P01106 | MYC Proto-oncogene, BHLH Transcription Factor | SymMap |
| 425 | MYNN | Q9NPC7 | Myoneurin | PharmMapper |
| 426 | NAGK | Q9UJ70 | N-acetyl-D-glucosamine kinase | PharmMapper |
| 427 | NAMPT | P43490 | Nicotinamide phosphoribosyltransferase | SwissTargetPrediction |
| 428 | NCOA1 | Q15788 | nuclear receptor coactivator 1 | SymMap |
| 429 | NCOA2 | Q15596 | Nuclear receptor coactivator 2 | TCMSP |
| 430 | NCSTN | Q92542 | Nicastrin | SwissTargetPrediction |
| 431 | NEK11 | Q8NG66 | NIMA related kinase 11 | SymMap |
| 432 | NFE2L2 | Q16236 | Nuclear Factor, Erythroid 2 Like 2 | SymMap |
| 433 | NFKBIA | P25963 | NFKB Inhibitor Alpha | SymMap |
| 434 | NLRP3 | Q96P20 | NACHT, LRR and PYD domains-containing protein 3 | SwissTargetPrediction |
| 435 | NOD1 | Q9Y239 | Nucleotide-binding oligomerization domain-containing protein 1 | SwissTargetPrediction |
| 436 | NOD2 | Q9HC29 | Nucleotide-binding oligomerization domain-containing protein 2 | SwissTargetPrediction |
| 437 | NOS2 | P35228 | nitric oxide synthase 2 | SymMap/SwissTargetPrediction |
| 438 | NOS3 | [P29474](https://www.uniprot.org/uniprot/P29474) | Nitric oxide synthase, endothelial | TCMSP/SymMap |
| 439 | NPC1L1 | Q9UHC9 | Niemann-Pick C1-like protein 1 | SwissTargetPrediction |
| 440 | NPS | P0C0P6 | Neuropeptide S | SymMap |
| 441 | NPSR1 | Q6W5P4 | neuropeptide S receptor 1 | SymMap |
| 442 | NPY5R | Q15761 | Neuropeptide Y receptor type 5 | SwissTargetPrediction |
| 443 | NR0B2 | Q15466 | Nuclear receptor subfamily 0 group B member 2 | SwissTargetPrediction |
| 444 | NR1D2 | Q14995 | Nuclear receptor subfamily 1 group D member 2 | PharmMapper |
| 445 | NR1H2 | P55055 | LXR-beta | SwissTargetPrediction |
| 446 | NR1H3 | Q13133 | LXR-alpha | SwissTargetPrediction |
| 447 | NR1H4 | Q96RI1 | Bile acid receptor FXR | SwissTargetPrediction/PubChem |
| 448 | NR1I2 | O75469 | Pregnane X receptor | SwissTargetPrediction |
| 449 | NR1I3 | Q14994 | Nuclear receptor subfamily 1 group I member 3 | SwissTargetPrediction/PharmMapper |
| 450 | NR3C1 | P04150 | Nuclear Receptor Subfamily 3 Group C Member 1 | SymMap/SwissTargetPrediction |
| 451 | NR3C2 | P08235 | Nuclear Receptor Subfamily 3 Group C Member 2 | SymMap/TCMSP/SwissTargetPrediction/PharmMapper |
| 452 | NRF1 | Q16656 | Nuclear Respiratory Factor 1 | SymMap |
| 453 | NTRK1 | P04629 | Nerve growth factor receptor Trk-A | SwissTargetPrediction |
| 454 | NTRK2 | Q16620 | Neurotrophic Receptor Tyrosine Kinase 2 | SymMap |
| 455 | NUDT5 | Q9UKK9 | ADP-sugar pyrophosphatase | PharmMapper |
| 456 | NUP214 | P35658 | Nuclear pore complex protein Nup214 | PharmMapper |
| 457 | OGG1 | O15527 | 8-oxoguanine DNA Glycosylase | SymMap |
| 458 | OPRD1 | P41143 | Delta opioid receptor | SwissTargetPrediction |
| 459 | OPRK1 | P41145 | Kappa Opioid receptor | SwissTargetPrediction |
| 460 | OPRL1 | P41146 | Nociceptin receptor | SwissTargetPrediction |
| 461 | OPRM1 | P35372 | opioid receptor mu 1 | SymMap/SwissTargetPrediction/TCMSP |
| 462 | OXER1 | Q8TDS5 | Oxoeicosanoid receptor 1 | SwissTargetPrediction |
| 463 | OXSR1 | O95747 | Serine/threonine-protein kinase OSR1 | PharmMapper |
| 464 | OXTR | P30559 | Oxytocin receptor (OT-R) | PubChem |
| 465 | P2RX3 | P56373 | P2X purinoceptor 3 | SwissTargetPrediction |
| 466 | PAM | P19021 | peptidylglycine alpha-amidating monooxygenase | SymMap |
| 467 | PARP2 | Q9UGN5 | Poly [ADP-ribose] polymerase 2 | SwissTargetPrediction |
| 468 | PCSK7 | Q16549 | Subtilisin/kexin type 7 | SwissTargetPrediction |
| 469 | PCSK9 | Q8NBP7 | Proprotein convertase subtilisin/kexin type 9 | PharmMapper |
| 470 | PCYT1A | P49585 | Phosphate Cytidylyltransferase 1, Choline, Alpha | SymMap |
| 471 | PDE10A | Q9Y233 | Phosphodiesterase 10A | SymMap/SwissTargetPrediction |
| 472 | PDE2A | O00408 | Phosphodiesterase 2A | SwissTargetPrediction |
| 473 | PDE3A | Q14432 | CGMP-inhibited 3',5'-cyclic phosphodiesterase A | TCMSP |
| 474 | PDE4A | P27815 | Phosphodiesterase 4A | SwissTargetPrediction |
| 475 | PDE4B | Q07343 | Phosphodiesterase 4B | SwissTargetPrediction |
| 476 | PDE4C | Q08493 | Phosphodiesterase 4C | SwissTargetPrediction |
| 477 | PDE4D | Q08499 | Phosphodiesterase 4D | SwissTargetPrediction |
| 478 | PDLIM1 | O00151 | PDZ and LIM domain protein 1 | PharmMapper |
| 479 | PDX1 | P52945 | Pancreatic And Duodenal Homeobox 1 | SymMap |
| 480 | PECAM1 | [P16284](https://www.uniprot.org/uniprot/P16284) | Platelet endothelial cell adhesion molecule | TCMSP/SymMap |
| 481 | PFKFB3 | Q16875 | 6-phosphofructo-2-kinase/fructose-2,6-bisphosphatase 3 | SwissTargetPrediction |
| 482 | PGD | P52209 | phosphogluconate dehydrogenase | SymMap |
| 483 | PGK1 | P00558 | Phosphoglycerate kinase 1 | PharmMapper |
| 484 | PGR | [P06401](https://www.uniprot.org/uniprot/P06401) | Progesterone receptor | TCMSP/SymMap/SwissTargetPrediction |
| 485 | PIK3CA | P42336 | PI3-kinase p110-alpha subunit | SwissTargetPrediction |
| 486 | PIK3CB | P42338 | PI3-kinase p110-beta subunit | SwissTargetPrediction |
| 487 | PIK3CD | O00329 | PI3-kinase p110-delta subunit | SwissTargetPrediction |
| 488 | PIK3CG | [P48736](https://www.uniprot.org/uniprot/P48736) | Phosphatidylinositol-4,5-bisphosphate 3-kinase catalytic subunit, gamma isoform | TCMSP/SwissTargetPrediction |
| 489 | PIM1 | P11309 | Serine/threonine-protein kinase PIM1 | SwissTargetPrediction |
| 490 | PIN1 | Q13526 | Peptidyl-prolyl cis-trans isomerase NIMA-interacting 1 | SwissTargetPrediction |
| 491 | PIP4K2C | Q8TBX8 | Phosphatidylinositol-5-phosphate 4-kinase type-2 gamma | PharmMapper |
| 492 | PLA2G10 | O15496 | Group X secretory phospholipase A2 | SwissTargetPrediction |
| 493 | PLA2G1B | P04054 | Phospholipase A2 group 1B | SwissTargetPrediction/STITCH |
| 494 | PLA2G2A | P14555 | phospholipase A2 group IIA | SymMap/SwissTargetPrediction/PharmMapper |
| 495 | PLA2G4A | [P47712](https://www.uniprot.org/uniprot/P47712) | Cytosolic phospholipase A2 | TCMSP/SymMap/SwissTargetPrediction |
| 496 | PLA2G7 | Q13093 | LDL-associated phospholipase A2 | SwissTargetPrediction/PubChem |
| 497 | PLAU | P00749 | Plasminogen Activator, Urokinase | SymMap |
| 498 | PLG | P00747 | Plasminogen | SymMap |
| 499 | PLK1 | P53350 | Serine/threonine-protein kinase PLK1 | SwissTargetPrediction |
| 500 | PNP | P00491 | Purine Nucleoside Phosphorylase | SymMap |
| 501 | POLA1 | P09884 | DNA polymerase alpha subunit | SwissTargetPrediction |
| 502 | POLB | P06746 | DNA polymerase beta | PubChem/SwissTargetPrediction |
| 503 | POLK | Q9UBT6 | DNA polymerase kappa | PubChem |
| 504 | PON1 | P27169 | Serum paraoxonase/arylesterase 1 | TCMSP/SymMap |
| 505 | PORCN | Q9H237 | Probable protein-cysteine N-palmitoyltransferase porcupine (by homology) | SwissTargetPrediction |
| 506 | POT1 | Q9NUX5 | Protection of telomeres protein 1 | PharmMapper |
| 507 | PPARA | Q07869 | peroxisome proliferator activated receptor alpha | SymMap/PubChem/SwissTargetPrediction |
| 508 | PPARD | Q03181 | peroxisome proliferator activated receptor delta | SymMap/SwissTargetPrediction/PubChem |
| 509 | PPARG | [P37231](https://www.uniprot.org/uniprot/P37231) | Peroxisome proliferator-activated receptor gamma | TCMSP/SymMap/SwissTargetPrediction/PubChem |
| 510 | PPARGC1B | Q86YN6 | PPARG Coactivator 1 Beta | SymMap |
| 511 | PPP2R1A | P30153 | Serine/threonine-protein phosphatase 2A 65 kDa regulatory subunit A alpha isoform | PharmMapper |
| 512 | PPP5C | P53041 | protein phosphatase 5 catalytic subunit | SymMap |
| 513 | PRDX6 | P30041 | Peroxiredoxin-6 | PharmMapper |
| 514 | PREP | P48147 | Prolyl endopeptidase | SwissTargetPrediction |
| 515 | PRKAA2 | P54646 | AMPK alpha2 | SwissTargetPrediction |
| 516 | PRKAB1 | Q9Y478 | AMPK beta1 | SwissTargetPrediction |
| 517 | PRKAG1 | P54619 | AMPK gamma1 | SwissTargetPrediction |
| 518 | PRKCA | P17252 | Protein kinase C alpha type | TCMSP/SwissTargetPrediction/STITCH |
| 519 | PRKCB | [P05771](https://www.uniprot.org/uniprot/P05771) | Protein kinase C beta type | TCMSP/SymMap/SwissTargetPrediction |
| 520 | PRKCD | Q05655 | Protein kinase C delta | SwissTargetPrediction |
| 521 | PRKCE | Q02156 | Protein kinase C epsilon | SwissTargetPrediction |
| 522 | PRKCG | P05129 | Protein kinase C gamma | SwissTargetPrediction |
| 523 | PRKCH | P24723 | Protein kinase C eta | SwissTargetPrediction |
| 524 | PRKCQ | Q04759 | Protein kinase C theta | SwissTargetPrediction |
| 525 | PRKCZ | Q05513 | Protein kinase C zeta type | PubChem |
| 526 | PRPS1 | P60891 | Ribose-phosphate pyrophosphokinase 1 | PharmMapper |
| 527 | PRPSAP1 | Q14558 | Phosphoribosyl pyrophosphate synthetase-associated protein 1 | PharmMapper |
| 528 | PRSS1 | P07477 | Serine Protease 1 | SymMap/SwissTargetPrediction |
| 529 | PSEN1 | P49768 | Presenilin 1 | SwissTargetPrediction |
| 530 | PSEN2 | P49810 | Gamma-secretase | SwissTargetPrediction |
| 531 | PSENEN | Q9NZ42 | Gamma-secretase subunit PEN-2 | SwissTargetPrediction |
| 532 | PTAFR | P25105 | Platelet activating factor receptor | SwissTargetPrediction |
| 533 | PTEN | [P60484](https://www.uniprot.org/uniprot/P60484) | Phosphatidylinositol-3,4,5-trisphosphate 3-phosphatase and dual-specificity protein phosphatase PTEN | TCMSP/SymMap |
| 534 | PTGDR | Q13258 | Prostanoid DP receptor | SwissTargetPrediction |
| 535 | PTGDR2 | Q9Y5Y4 | G protein-coupled receptor 44 | SwissTargetPrediction |
| 536 | PTGER1 | P34995 | Prostanoid EP1 receptor | SwissTargetPrediction |
| 537 | PTGER2 | P43116 | Prostanoid EP2 receptor | SwissTargetPrediction |
| 538 | PTGER3 | P43115 | Prostanoid EP3 receptor | SwissTargetPrediction |
| 539 | PTGER4 | P35408 | Prostanoid EP4 receptor | SwissTargetPrediction |
| 540 | PTGES | [O14684](https://www.uniprot.org/uniprot/O14684) | Prostaglandin E synthase | TCMSP/SymMap/SwissTargetPrediction |
| 541 | PTGES2 | [Q9H7Z7](https://www.uniprot.org/uniprot/Q9H7Z7) | Prostaglandin E synthase 2 | TCMSP/SymMap/SwissTargetPrediction |
| 542 | PTGFR | P43088 | Prostanoid FP receptor | SwissTargetPrediction |
| 543 | PTGIR | P43119 | Prostanoid IP receptor | SwissTargetPrediction |
| 544 | PTGS1 | P23219 | Prostaglandin G/H synthase 1 | TCMSP/SymMap/SwissTargetPrediction/PubChem |
| 545 | PTGS2 | P35354 | Prostaglandin G/H synthase 2 | TCMSP/SymMap/SwissTargetPrediction/PubChem/STITCH |
| 546 | PTPN1 | P18031 | Protein-tyrosine phosphatase 1B | SwissTargetPrediction |
| 547 | PTPN11 | Q06124 | Protein-tyrosine phosphatase 2C | SwissTargetPrediction |
| 548 | PTPN2 | P17706 | T-cell protein-tyrosine phosphatase | SwissTargetPrediction |
| 549 | PTPN6 | P29350 | Protein-tyrosine phosphatase 1C | SwissTargetPrediction |
| 550 | PTPRF | P10586 | Receptor-type tyrosine-protein phosphatase F (LAR) | SwissTargetPrediction |
| 551 | PUS10 | Q3MIT2 | Putative tRNA pseudouridine synthase Pus10 | PharmMapper |
| 552 | PYGL | P06737 | Glycogen Phosphorylase L | SymMap/SwissTargetPrediction |
| 553 | PYY | P10082 | Peptide YY | SymMap |
| 554 | QPCT | Q16769 | Glutaminyl-peptide cyclotransferase | SwissTargetPrediction |
| 555 | RAB7A | P51149 | Ras-related protein Rab-7a | PharmMapper |
| 556 | RAF1 | P04049 | Serine/threonine-protein kinase RAF | SwissTargetPrediction |
| 557 | RANBP2 | P49792 | E3 SUMO-protein ligase RanBP2 | PharmMapper |
| 558 | RARA | P10276 | Retinoic acid receptor alpha | SwissTargetPrediction |
| 559 | RARB | P10826 | Retinoic acid receptor beta | SwissTargetPrediction/PharmMapper |
| 560 | RARG | P13631 | Retinoic acid receptor gamma | SwissTargetPrediction/PharmMapper |
| 561 | RASGRF1 | Q13972 | Ras Protein Specific Guanine Nucleotide Releasing Factor 1 | SymMap |
| 562 | RBP2 | P50120 | Retinol Binding Protein 2 | SymMap |
| 563 | RBP4 | P02753 | Plasma retinol-binding protein | SwissTargetPrediction |
| 564 | RCE1 | Q9Y256 | Prenyl protein specific protease | SwissTargetPrediction |
| 565 | RELA | [Q04206](https://www.uniprot.org/uniprot/Q04206) | Transcription factor p65 | TCMSP/SymMap |
| 566 | RENBP | P51606 | renin binding protein | SymMap |
| 567 | REPS2 | Q8NFH8 | RalBP1-associated Eps domain-containing protein 2 | PharmMapper |
| 568 | RETN | Q9HD89 | Resistin | SymMap |
| 569 | REV1 | Q9UBZ9 | DNA repair protein REV1 | PharmMapper |
| 570 | RGS18 | Q9NS28 | Regulator of G-protein signaling 18 | PharmMapper |
| 571 | RGS6 | P49758 | Regulator of G-protein signaling 6 | PharmMapper |
| 572 | ROCK1 | Q13464 | Rho-associated protein kinase 1 | SwissTargetPrediction |
| 573 | ROCK2 | O75116 | Rho-associated protein kinase 2 | SwissTargetPrediction |
| 574 | RORA | P35398 | Nuclear receptor ROR-alpha | SwissTargetPrediction |
| 575 | RORC | P51449 | Nuclear receptor ROR-gamma | SwissTargetPrediction |
| 576 | RPSA | P08865 | 40S ribosomal protein SA | PharmMapper |
| 577 | RRM2B | Q7LG56 | Ribonucleoside-diphosphate reductase subunit M2 B | PharmMapper |
| 578 | RUNX1T1 | Q06455 | RUNX1 Translocation Partner 1 | SymMap/PharmMapper |
| 579 | RUVBL1 | Q9Y265 | RuvB-like 1 | PharmMapper |
| 580 | RXRA | [P19793](https://www.uniprot.org/uniprot/P19793) | Retinoic acid receptor RXR-alpha | TCMSP/SymMap/SwissTargetPrediction |
| 581 | RXRB | P28702 | Retinoid X receptor beta | SwissTargetPrediction/PharmMapper |
| 582 | RXRG | [P48443](https://www.uniprot.org/uniprot/P48443) | Retinoic acid receptor RXR-gamma | TCMSP/SwissTargetPrediction/PharmMapper |
| 583 | S100A12 | P80511 | Protein S100-A12 | PharmMapper |
| 584 | S100A6 | P06703 | Protein S100-A6 | PharmMapper |
| 585 | S1PR1 | P21453 | Sphingosine 1-phosphate receptor Edg-1 | SwissTargetPrediction |
| 586 | S1PR3 | Q99500 | Sphingosine 1-phosphate receptor Edg-3 | SwissTargetPrediction |
| 587 | SATB2 | Q9UPW6 | DNA-binding protein SATB2 | PharmMapper |
| 588 | SCD | O00767 | Stearoyl-CoA Desaturase | SymMap/SwissTargetPrediction |
| 589 | SCN5A | Q14524 | Sodium channel protein type 5 subunit alpha | TCMSP/SymMap |
| 590 | SCN9A | Q15858 | Sodium channel protein type IX alpha subunit | SwissTargetPrediction |
| 591 | SELP | [P16109](https://www.uniprot.org/uniprot/P16109) | P-selectin | TCMSP/SymMap |
| 592 | SEM1 | P60896 | 26S proteasome complex subunit DSS1 | PharmMapper |
| 593 | SENP7 | Q9BQF6 | Sentrin-specific protease 7 | PharmMapper |
| 594 | SERPINA6 | P08185 | Corticosteroid binding globulin | SwissTargetPrediction |
| 595 | SERPINE1 | P05121 | Serpin Family E Member 1 | SymMap |
| 596 | SERPING1 | P05155 | Plasma protease C1 inhibitor | PharmMapper |
| 597 | SF1 | Q15637 | Splicing factor 1 | PharmMapper |
| 598 | SGPL1 | O95470 | Sphingosine-1-phosphate lyase 1 | SwissTargetPrediction |
| 599 | SH2B2 | O14492 | SH2B adapter protein 2 | PharmMapper |
| 600 | SHBG | P04278 | Testis-specific androgen-binding protein | SwissTargetPrediction |
| 601 | SHH | Q15465 | Sonic hedgehog protein (by homology) | SwissTargetPrediction |
| 602 | SIGMAR1 | Q99720 | Sigma opioid receptor | SwissTargetPrediction |
| 603 | SKP1 | P63208 | S-phase kinase-associated protein 1 | PharmMapper |
| 604 | SLC10A2 | Q12908 | Ileal bile acid transporter | SwissTargetPrediction |
| 605 | SLC16A1 | P53985 | Monocarboxylate transporter 1 | SwissTargetPrediction |
| 606 | SLC22A12 | Q96S37 | Solute carrier family 22 member 12 | SwissTargetPrediction |
| 607 | SLC22A5 | O76082 | Solute Carrier Family 22 Member 5 | SymMap |
| 608 | SLC22A6 | Q4U2R8 | Solute carrier family 22 member 6 (by homology) | SwissTargetPrediction |
| 609 | SLC2A2 | P11168 | Solute Carrier Family 2 Member 2 | SymMap |
| 610 | SLC2A4 | P14672 | Solute Carrier Family 2 Member 4 | SymMap |
| 611 | SLC5A1 | P13866 | Sodium/glucose cotransporter 1 | SwissTargetPrediction |
| 612 | SLC6A2 | [P23975](https://www.uniprot.org/uniprot/P23975) | Sodium-dependent noradrenaline transporter | TCMSP/SwissTargetPrediction/SymMap |
| 613 | SLC6A3 | Q01959 | Solute Carrier Family 6 Member 3 | SymMap/SwissTargetPrediction |
| 614 | SLC6A4 | P31645 | Sodium-dependent serotonin transporter | TCMSP/SwissTargetPrediction/SymMap |
| 615 | SLC7A7 | Q9UM01 | Solute Carrier Family 7 Member 7 | SymMap |
| 616 | SLCO1B1 | Q9Y6L6 | Solute carrier organic anion transporter family member 1B1 | PubChem |
| 617 | SLCO1B3 | Q9NPD5 | Solute carrier organic anion transporter family member 1B3 | PubChem |
| 618 | SLK | Q9H2G2 | STE20-like serine/threonine-protein kinase | PharmMapper |
| 619 | SMARCC1 | Q92922 | SWI/SNF complex subunit SMARCC1 | PharmMapper |
| 620 | SMG7 | Q92540 | Protein SMG7 | PharmMapper |
| 621 | SMO | Q99835 | Smoothened homolog | SwissTargetPrediction |
| 622 | SNF8 | Q96H20 | Vacuolar-sorting protein SNF8 | PharmMapper |
| 623 | SOAT1 | P35610 | sterol O-acyltransferase 1 | SymMap/SwissTargetPrediction |
| 624 | SOAT2 | O75908 | Acyl coenzyme A:cholesterol acyltransferase 2 | SwissTargetPrediction |
| 625 | SOD1 | P00441 | Superoxide Dismutase 1 | SymMap |
| 626 | SOD2 | P04179 | Superoxide dismutase [Mn], mitochondrial | PharmMapper |
| 627 | SOD3 | P08294 | Superoxide Dismutase 3 | SymMap |
| 628 | SP1 | P08047 | Sp1 Transcription Factor | SymMap |
| 629 | SPEN | Q96T58 | Msx2-interacting protein | PharmMapper |
| 630 | SQLE | Q14534 | Squalene monooxygenase | SwissTargetPrediction |
| 631 | SRC | P12931 | Tyrosine-protein kinase SRC | SwissTargetPrediction |
| 632 | SRD5A2 | P31213 | Steroid 5-alpha-reductase 2 | SwissTargetPrediction |
| 633 | SREBF2 | Q12772 | Sterol regulatory element-binding protein 2 | SwissTargetPrediction |
| 634 | SRI | P30626 | Sorcin | PharmMapper |
| 635 | SRSF1 | Q07955 | Splicing factor, arginine/serine-rich 1 | PharmMapper |
| 636 | STAM2 | O75886 | Signal transducing adapter molecule 2 | PharmMapper |
| 637 | STAP1 | Q9ULZ2 | Signal-transducing adaptor protein 1 | PharmMapper |
| 638 | STARD3 | Q14849 | StAR-related lipid transfer protein 3 | PharmMapper |
| 639 | STS | P08842 | Steryl-sulfatase | SwissTargetPrediction |
| 640 | SUB1 | P53999 | Activated RNA polymerase II transcriptional coactivator p15 | PharmMapper |
| 641 | SULT1B1 | O43704 | Sulfotransferase family cytosolic 1B member 1 | PharmMapper |
| 642 | SUOX | P51687 | Sulfite oxidase, mitochondrial | PharmMapper |
| 643 | SYNCRIP | O60506 | Heterogeneous nuclear ribonucleoprotein Q | PharmMapper |
| 644 | TACR1 | P25103 | Neurokinin 1 receptor | SwissTargetPrediction |
| 645 | TACR2 | P21452 | Neurokinin 2 receptor | SwissTargetPrediction |
| 646 | TAF13 | Q15543 | Transcription initiation factor TFIID subunit 13 | PharmMapper |
| 647 | TAS2R31 | P59538 | Taste receptor type 2 member 31 | SwissTargetPrediction |
| 648 | TBXA2R | P21731 | Thromboxane A2 receptor | SwissTargetPrediction/STITCH |
| 649 | TBXAS1 | P24557 | Thromboxane-A synthase | SwissTargetPrediction |
| 650 | TDP1 | Q9NUW8 | Tyrosyl-DNA Phosphodiesterase 1 | SymMap/PubChem |
| 651 | TEP1 | Q99973 | Telomerase Associated Protein 1 | SymMap |
| 652 | TERT | O14746 | Telomerase reverse transcriptase | SwissTargetPrediction |
| 653 | TFAM | Q00059 | Transcription Factor A, Mitochondrial | SymMap |
| 654 | TGFB1 | P01137 | Transforming growth factor beta-1 | TCMSP |
| 655 | TGFBR1 | P36897 | TGF-beta receptor type I | SwissTargetPrediction |
| 656 | TGFBR2 | P37173 | TGF-beta receptor type II | SwissTargetPrediction |
| 657 | TH | P07101 | Tyrosine Hydroxylase | SymMap |
| 658 | THOP1 | P52888 | Thimet oligopeptidase | PharmMapper |
| 659 | THRB | P10828 | Prothrombin | PharmMapper |
| 660 | TIMM9 | Q9Y5J7 | Mitochondrial import inner membrane translocase subunit Tim9 | PharmMapper |
| 661 | TJP1 | Q07157 | Tight junction protein ZO-1 | PharmMapper |
| 662 | TLR4 | O00206 | Toll Like Receptor 4 | SymMap |
| 663 | TLR9 | Q9NR96 | Toll-like receptor (TLR7/TLR9) | SwissTargetPrediction |
| 664 | TNC | P24821 | Tenascin C | SymMap |
| 665 | TNF | P01375 | tumor necrosis factor | SymMap/SwissTargetPrediction |
| 666 | TNFRSF1A | [P19438](https://www.uniprot.org/uniprot/P19438) | Tumor necrosis factor receptor superfamily member 1A | TCMSP/SymMap |
| 667 | TNFRSF1B | [P20333](https://www.uniprot.org/uniprot/P20333) | Tumor necrosis factor receptor superfamily member 1B | TCMSP/SymMap |
| 668 | TNKS | O95271 | Tankyrase-1 | SwissTargetPrediction |
| 669 | TOP1 | P11387 | DNA topoisomerase I | SwissTargetPrediction |
| 670 | TOP2A | P11388 | DNA topoisomerase II alpha | SwissTargetPrediction |
| 671 | TP53 | P04637 | Tumor Protein P53 | SymMap |
| 672 | TP73 | O15350 | Tumor protein p73 | PharmMapper |
| 673 | TPK1 | Q9H3S4 | Thiamin pyrophosphokinase 1 | PharmMapper |
| 674 | TRPA1 | O75762 | Transient receptor potential cation channel subfamily A member 1 | SwissTargetPrediction |
| 675 | TRPC6 | Q9Y210 | Short transient receptor potential channel 6 | PubChem |
| 676 | TRPM2 | O94759 | Transient receptor potential cation channel subfamily M member 2 | PubChem |
| 677 | TRPM8 | Q7Z2W7 | Transient receptor potential cation channel subfamily M member 8 | SwissTargetPrediction |
| 678 | TRPV1 | Q8NER1 | transient receptor potential cation channel subfamily V member 1 | SymMap/SwissTargetPrediction/TCMSP |
| 679 | TRPV4 | Q9HBA0 | Transient Receptor Potential Cation Channel Subfamily V Member 4 | SymMap/STITCH |
| 680 | TSPO | P30536 | Translocator protein (by homology) | SwissTargetPrediction |
| 681 | TTK | P33981 | Dual specificity protein kinase TTK | SwissTargetPrediction |
| 682 | TTL | Q8NG68 | Tubulin--tyrosine ligase | SwissTargetPrediction |
| 683 | TXNRD1 | Q16881 | thioredoxin reductase 1 | SymMap |
| 684 | UAP1 | Q16222 | UDP-N-acetylhexosamine pyrophosphorylase | PharmMapper |
| 685 | UCP2 | P55851 | Mitochondrial uncoupling protein 2 | TCMSPSymMap |
| 686 | UCP3 | P55916 | Uncoupling Protein 3 | SymMap |
| 687 | UGT2B7 | P16662 | UDP-glucuronosyltransferase 2B7 | SwissTargetPrediction |
| 688 | UPF1 | Q92900 | Regulator of nonsense transcripts 1 | PharmMapper |
| 689 | UROS | P10746 | Uroporphyrinogen-III synthase | PharmMapper |
| 690 | USH1C | Q9Y6N9 | Harmonin | PharmMapper |
| 691 | USP1 | Q92995 | Ubiquitin carboxyl-terminal hydrolase 13 | PubChem |
| 692 | USP14 | P54578 | Ubiquitin carboxyl-terminal hydrolase 14 | PharmMapper |
| 693 | USP19 | O94966 | Ubiquitin carboxyl-terminal hydrolase 19 | PharmMapper |
| 694 | VAV2 | P52735 | Guanine nucleotide exchange factor VAV2 | PharmMapper |
| 695 | VCAM1 | P19320 | vascular cell adhesion molecule 1 | SymMap |
| 696 | VDR | P11473 | Vitamin D receptor | SwissTargetPrediction/PubChem |
| 697 | VEGFA | P15692 | vascular endothelial growth factor A | SymMap/PharmMapper |
| 698 | VEGFB | P49765 | Vascular endothelial growth factor B | PharmMapper |
| 699 | VRK2 | Q86Y07 | Serine/threonine-protein kinase VRK2 | PharmMapper |
| 700 | WDTC1 | Q8N5D0 | WD and tetratricopeptide repeats protein 1 | STITCH |
| 701 | XRCC6 | P12956 | ATP-dependent DNA helicase 2 subunit 1 | PharmMapper |
| 702 | YES1 | P07947 | Tyrosine-protein kinase YES | SwissTargetPrediction |
| 703 | ZBTB16 | Q05516 | Zinc finger and BTB domain-containing protein 16 | PharmMapper |
| 704 | ZBTB43 | O43298 | Zinc finger and BTB domain-containing protein 43 | PharmMapper |
| 705 | ZEB2 | O60315 | Zinc finger E-box-binding homeobox 2 | PharmMapper |
| 706 | ZFHX3 | Q15911 | Zinc Finger Homeobox 3 | SymMap |
| 707 | ZNF462 | Q96JM2 | Zinc finger protein 462 | PharmMapper |
