# Supplementary material for: Protective effect of Cordyceps sinensis against diabetic kidney disease through promoting proliferation and inhibiting apoptosis of renal proximal tubular cells
Source: BMC Complement Med Ther. 2023 Apr 6;23:109. doi: 10.1186/s12906-023-03901-4 (PMC10077712; doi:10.1186/s12906-023-03901-4)
Supplement: Supplementary file 2 — Additional file 2: Table S2. DKD related targets. [file 12906_2023_3901_MOESM2_ESM.docx]

**Table S2** DKD related targets

| **Number** | **Gene** | **UniProt ID** | **Gene_Full_Name** | **source** |
| --- | --- | --- | --- | --- |
| 1 | FOXO1 | Q12778 | forkhead box O1 | DisGeNet/OMIM |
| 2 | KCNJ11 | Q14654 | ATP-sensitive inward rectifier potassium channel 11 | GeneCards/OMIM |
| 3 | NEUROD1 | Q13562 | Neuronal Differentiation 1 | GeneCards/OMIM |
| 4 | NOS2 | P35228 | nitric oxide synthase 2 | DisGeNet/CTD/OMIM |
| 5 | PAX4 | O43316 | Paired Box 4 | GeneCards/OMIM |
| 6 | PDX1 | P52945 | Pancreatic And Duodenal Homeobox 1 | GeneCards/OMIM |
| 7 | MARCH3 | Q86UD3 | membrane associated ring-CH-type finger 3 | DisGeNet |
| 8 | ABCB1 | P08183 | Multidrug resistance protein 1 | DrugBank |
| 9 | ABCB11 | O95342 | Bile salt export pump | DrugBank |
| 10 | ABCC2 | Q92887 | Canalicular multispecific organic anion transporter 1 | DrugBank |
| 11 | ABCC9 | O60706 | ATP-binding cassette sub-family C member 9 | OMIM |
| 12 | ABCG2 | Q9UNQ0 | ATP-binding cassette sub-family G member 2 | DrugBank |
| 13 | ABO | P16442 | Histo-blood group ABO system transferase | OMIM |
| 14 | ACACB | O00763 | acetyl-CoA carboxylase beta | DisGeNet |
| 15 | ACADM | P11310 | Medium-chain specific acyl-CoA dehydrogenase | OMIM |
| 16 | ACADS | P16219 | Short-chain specific acyl-CoA dehydrogenase | OMIM |
| 17 | ACE2 | Q9BYF1 | angiotensin I converting enzyme 2 | DisGeNet |
| 18 | ACMSD | Q8TDX5 | 2-amino-3-carboxymuconate-6-semialdehyde decarboxylase | OMIM |
| 19 | ACP5 | P13686 | Tartrate-resistant acid phosphatase type 5 | OMIM |
| 20 | ACSS1 | Q9NUB1 | Acetyl-coenzyme A synthetase 2-like | OMIM |
| 21 | ACSS2 | Q9NR19 | Acetyl-coenzyme A synthetase | OMIM |
| 22 | ACTA2 | P62736 | Actin, aortic smooth muscle | CTD |
| 23 | ACTG2 | P63267 | actin, gamma 2, smooth muscle, enteric | DisGeNet |
| 24 | ACVRL1 | P37023 | activin A receptor like type 1 | DisGeNet |
| 25 | ADA | P00813 | Adenosine deaminase | OMIM |
| 26 | ADAM10 | O14672 | ADAM metallopeptidase domain 10 | DisGeNet |
| 27 | ADAM17 | P78536 | ADAM metallopeptidase domain 17 | DisGeNet |
| 28 | ADAMTS13 | Q76LX8 | ADAM metallopeptidase with thrombospondin type 1 motif 13 | DisGeNet |
| 29 | ADCY3 | O60266 | Adenylate cyclase type 3 | OMIM |
| 30 | ADD1 | P35611 | adducin 1 | DisGeNet |
| 31 | ADD2 | P35612 | adducin 2 | DisGeNet |
| 32 | ADH1B | P00325 | alcohol dehydrogenase 1B (class I), beta polypeptide | DisGeNet |
| 33 | ADIPOR1 | Q96A54 | Adiponectin receptor protein 1 | OMIM |
| 34 | ADIPOR2 | Q86V24 | Adiponectin receptor protein 2 | OMIM |
| 35 | ADM | P35318 | adrenomedullin | DisGeNet |
| 36 | ADRA2A | P08913 | Alpha-2A adrenergic receptor | OMIM |
| 37 | ADRA2C | P18825 | Alpha-2C adrenergic receptor | TTD |
| 38 | ADRB3 | P13945 | Beta-3 adrenergic receptor | OMIM |
| 39 | AGTR1 | P30556 | angiotensin II receptor type 1 | DisGeNet/GeneCards/DrugBank |
| 40 | AGTR2 | P50052 | angiotensin II receptor type 2 | DisGeNet |
| 41 | AKR1A1 | P14550 | aldo-keto reductase family 1 member A1 | DisGeNet |
| 42 | AKR1B1 | P15121 | aldo-keto reductase family 1 member B | DisGeNet/OMIM |
| 43 | AKT1 | P31749 | AKT serine/threonine kinase 1 | DisGeNet/CTD/OMIM |
| 44 | ALB | P02768 | albumin | DisGeNet/GeneCards/CTD/DrugBank |
| 45 | ALDH2 | P05091 | aldehyde dehydrogenase 2 family member | DisGeNet |
| 46 | ALOX12 | P18054 | arachidonate 12-lipoxygenase, 12S type | DisGeNet/OMIM |
| 47 | ALPK1 | Q96QP1 | alpha kinase 1 | DisGeNet |
| 48 | ANG | P03950 | angiogenin | DisGeNet |
| 49 | ANGPT1 | Q15389 | angiopoietin 1 | DisGeNet |
| 50 | ANGPT2 | O15123 | angiopoietin 2 | DisGeNet/OMIM |
| 51 | ANKRD1 | Q15327 | ankyrin repeat domain 1 | DisGeNet |
| 52 | ANKRD23 | Q86SG2 | Ankyrin repeat domain-containing protein 23 | OMIM |
| 53 | AOC3 | Q16853 | amine oxidase, copper containing 3 | DisGeNet |
| 54 | APLNR | P35414 | apelin receptor | DisGeNet |
| 55 | APOA1 | P02647 | Apolipoprotein A-I | OMIM |
| 56 | APOA5 | Q6Q788 | apolipoprotein A5 | DisGeNet/OMIM |
| 57 | APOB | P04114 | apolipoprotein B | DisGeNet |
| 58 | APOC1 | P02654 | apolipoprotein C1 | DisGeNet |
| 59 | APOC3 | P02656 | apolipoprotein C3 | DisGeNet/OMIM |
| 60 | APOE | P02649 | apolipoprotein E | DisGeNet/OMIM |
| 61 | APOL1 | O14791 | apolipoprotein L1 | DisGeNet/OMIM |
| 62 | AQP1 | P29972 | aquaporin 1 (Colton blood group) | DisGeNet |
| 63 | AQP2 | P41181 | aquaporin 2 | DisGeNet/OMIM |
| 64 | AQP5 | P55064 | aquaporin 5 | DisGeNet |
| 65 | ARRB1 | P49407 | arrestin beta 1 | DisGeNet |
| 66 | ARRB2 | P32121 | arrestin beta 2 | DisGeNet/OMIM |
| 67 | ATF6 | P18850 | activating transcription factor 6 | DisGeNet/OMIM |
| 68 | ATM | Q13315 | ATM serine/threonine kinase | DisGeNet |
| 69 | ATP5F1B | P06576 | ATP synthase F1 subunit beta | DisGeNet |
| 70 | ATP5MD | Q96IX5 | ATP synthase membrane subunit DAPIT, mitochondrial | OMIM |
| 71 | ATP6AP2 | O75787 | ATPase H+ transporting accessory protein 2 | DisGeNet |
| 72 | AXIN2 | Q9Y2T1 | axin 2 | DisGeNet |
| 73 | AXL | P30530 | AXL receptor tyrosine kinase | DisGeNet |
| 74 | BASP1 | P80723 | brain abundant membrane attached signal protein 1 | DisGeNet |
| 75 | BAX | Q07812 | Apoptosis regulator BAX | CTD |
| 76 | BCHE | P06276 | Cholinesterase | DrugBank/OMIM |
| 77 | BCL2 | P10415 | BCL2, apoptosis regulator | DisGeNet/CTD |
| 78 | BCR | P11274 | BCR, RhoGEF and GTPase activating protein | DisGeNet |
| 79 | BDKRB1 | P46663 | B1 bradykinin receptor | DrugBank |
| 80 | BDKRB2 | P30411 | B2 bradykinin receptor (B2R) | OMIM |
| 81 | BEST1 | O76090 | bestrophin 1 | DisGeNet |
| 82 | BGN | P21810 | biglycan | DisGeNet |
| 83 | BMP2 | P12643 | bone morphogenetic protein 2 | DisGeNet |
| 84 | BMP4 | P12644 | bone morphogenetic protein 4 | DisGeNet |
| 85 | BMP7 | P18075 | bone morphogenetic protein 7 | DisGeNet |
| 86 | BRD2 | P25440 | bromodomain containing 2 | DisGeNet |
| 87 | CALCRL | Q16602 | calcitonin receptor like receptor | DisGeNet |
| 88 | CALD1 | Q05682 | caldesmon 1 | DisGeNet |
| 89 | CARS | P49589 | cysteinyl-tRNA synthetase | DisGeNet |
| 90 | CASP12 | Q6UXS9 | caspase 12 (gene/pseudogene) | DisGeNet |
| 91 | CASP9 | P55211 | Caspase-9 | CTD |
| 92 | CAT | P04040 | catalase | DisGeNet/CTD/OMIM |
| 93 | CAV1 | Q03135 | caveolin 1 | DisGeNet/OMIM |
| 94 | CCHCR1 | Q8TD31 | coiled-coil alpha-helical rod protein 1 | DisGeNet |
| 95 | CCL2 | P13500 | C-C motif chemokine ligand 2 | DisGeNet/CTD |
| 96 | CCL20 | P78556 | C-C motif chemokine ligand 20 | DisGeNet |
| 97 | CCL5 | P13501 | C-C motif chemokine ligand 5 | DisGeNet/OMIM |
| 98 | CCN2 | P29279 | Connective tissue growth factor | TTD |
| 99 | CCR1 | P32246 | C-C motif chemokine receptor 1 | DisGeNet |
| 100 | CCR2 | P41597 | C-C motif chemokine receptor 2 | DisGeNet/TTD |
| 101 | CCR5 | P51681 | C-C motif chemokine receptor 5 (gene/pseudogene) | DisGeNet/OMIM |
| 102 | CD2AP | Q9Y5K6 | CD2 associated protein | DisGeNet |
| 103 | CD36 | P16671 | CD36 molecule | DisGeNet/OMIM |
| 104 | CD44 | P16070 | CD44 molecule (Indian blood group) | DisGeNet/OMIM |
| 105 | CD59 | P13987 | CD59 molecule (CD59 blood group) | DisGeNet/OMIM |
| 106 | CD74 | P04233 | CD74 molecule | DisGeNet |
| 107 | CDC42 | P60953 | cell division cycle 42 | DisGeNet |
| 108 | CDH2 | P19022 | cadherin 2 | DisGeNet |
| 109 | CDKAL1 | Q5VV42 | CDK5 regulatory subunit associated protein 1 like 1 | DisGeNet/OMIM |
| 110 | CDKN1A | P38936 | cyclin dependent kinase inhibitor 1A | DisGeNet |
| 111 | CDKN2A | P42771;Q8N726 | cyclin dependent kinase inhibitor 2A | DisGeNet/OMIM |
| 112 | CES1 | P23141 | Liver carboxylesterase 1 | DrugBank |
| 113 | CETP | P11597 | cholesteryl ester transfer protein | DisGeNet |
| 114 | CFB | P00751 | Complement factor B | OMIM |
| 115 | CIC | Q96RK0 | capicua transcriptional repressor | DisGeNet |
| 116 | CNDP1 | Q96KN2 | carnosine dipeptidase 1 | DisGeNet |
| 117 | CNDP2 | Q96KP4 | carnosine dipeptidase 2 | DisGeNet |
| 118 | CNKSR3 | Q6P9H4 | CNKSR family member 3 | DisGeNet |
| 119 | CNR1 | P21554 | cannabinoid receptor 1 | DisGeNet |
| 120 | COL1A2 | P08123 | collagen type I alpha 2 chain | DisGeNet |
| 121 | COL4A1 | P02462 | collagen type IV alpha 1 chain | DisGeNet/OMIM |
| 122 | COL8A1 | P27658 | collagen type VIII alpha 1 chain | DisGeNet |
| 123 | COL8A2 | P25067 | collagen type VIII alpha 2 chain | DisGeNet |
| 124 | COX1 | P00395 | cytochrome c oxidase subunit I | DisGeNet |
| 125 | COX8A | P10176 | cytochrome c oxidase subunit 8A | DisGeNet |
| 126 | CREB1 | P16220 | cAMP responsive element binding protein 1 | DisGeNet/OMIM |
| 127 | CREM | Q03060 | cAMP responsive element modulator | DisGeNet |
| 128 | CRISP2 | P16562 | cysteine rich secretory protein 2 | DisGeNet |
| 129 | CRMP1 | Q14194 | collapsin response mediator protein 1 | DisGeNet |
| 130 | CRTC1 | Q6UUV9 | CREB regulated transcription coactivator 1 | DisGeNet |
| 131 | CST3 | P01034 | cystatin C | DisGeNet |
| 132 | CTGF | P29279 | connective tissue growth factor | DisGeNet/OMIM |
| 133 | CTNNB1 | P35222 | catenin beta 1 | DisGeNet |
| 134 | CTSB | P07858 | cathepsin B | DisGeNet |
| 135 | CUBN | O60494 | cubilin | DisGeNet |
| 136 | CUL5 | Q93034 | cullin 5 | DisGeNet |
| 137 | CXCL10 | P02778 | C-X-C motif chemokine ligand 10 | DisGeNet/OMIM |
| 138 | CXCL11 | O14625 | C-X-C motif chemokine ligand 11 | DisGeNet |
| 139 | CXCL8 | P10145 | C-X-C motif chemokine ligand 8 | DisGeNet |
| 140 | CXCL9 | Q07325 | C-X-C motif chemokine ligand 9 | DisGeNet |
| 141 | CXCR4 | P61073 | C-X-C motif chemokine receptor 4 | DisGeNet/OMIM |
| 142 | CYBA | P13498 | cytochrome b-245 alpha chain | DisGeNet/CTD |
| 143 | CYBB | P04839 | cytochrome b-245 beta chain | DisGeNet |
| 144 | CYP11B2 | P19099 | cytochrome P450 family 11 subfamily B member 2 | DisGeNet |
| 145 | CYP24A1 | Q07973 | cytochrome P450 family 24 subfamily A member 1 | DisGeNet |
| 146 | CYP27B1 | O15528 | cytochrome P450 family 27 subfamily B member 1 | DisGeNet |
| 147 | CYP2C19 | P33261 | Cytochrome P450 2C19 | DrugBank |
| 148 | CYP2C8 | P10632 | Cytochrome P450 2C8 | DrugBank |
| 149 | CYP2C9 | P11712 | Cytochrome P450 2C9 | DrugBank/OMIM |
| 150 | CYP2R1 | Q6VVX0 | cytochrome P450 family 2 subfamily R member 1 | DisGeNet |
| 151 | CYP3A4 | P08684 | Cytochrome P450 3A4 | DrugBank |
| 152 | CYP4B1 | P13584 | cytochrome P450 family 4 subfamily B member 1 | DisGeNet |
| 153 | DAPK2 | Q9UIK4 | death associated protein kinase 2 | DisGeNet |
| 154 | DCN | P07585 | decorin | DisGeNet |
| 155 | DCTN4 | Q9UJW0 | dynactin subunit 4 | DisGeNet |
| 156 | DDAH1 | O94760 | dimethylarginine dimethylaminohydrolase 1 | DisGeNet |
| 157 | DDAH2 | O95865 | dimethylarginine dimethylaminohydrolase 2 | DisGeNet |
| 158 | DDOST | P39656 | dolichyl-diphosphooligosaccharide--protein glycosyltransferase non-catalytic subunit | DisGeNet/OMIM |
| 159 | DECR1 | Q16698 | 2,4-dienoyl-CoA reductase 1 | DisGeNet |
| 160 | DEFA1 | P59665 | defensin alpha 1 | DisGeNet |
| 161 | DEFA3 | P59666 | defensin alpha 3 | DisGeNet |
| 162 | DENR | O43583 | density regulated re-initiation and release factor | DisGeNet |
| 163 | DGKH | Q86XP1 | diacylglycerol kinase eta | DisGeNet |
| 164 | DKK1 | O94907 | dickkopf WNT signaling pathway inhibitor 1 | DisGeNet |
| 165 | DNM1L | O00429 | dynamin 1 like | DisGeNet |
| 166 | DOT1L | Q8TEK3 | DOT1 like histone lysine methyltransferase | DisGeNet |
| 167 | DPP4 | P27487 | dipeptidyl peptidase 4 | DisGeNet/OMIM |
| 168 | DRD3 | P35462 | dopamine receptor D3 | DisGeNet |
| 169 | E2F1 | Q01094 | E2F transcription factor 1 | DisGeNet/OMIM |
| 170 | EBI3 | Q14213 | Epstein-Barr virus induced 3 | DisGeNet |
| 171 | ECE1 | P42892 | endothelin converting enzyme 1 | DisGeNet/OMIM |
| 172 | EDA | Q92838 | ectodysplasin A | DisGeNet |
| 173 | EDN1 | P05305 | endothelin 1 | DisGeNet/CTD |
| 174 | EDNRA | P25101 | Endothelin-1 receptor | TTD |
| 175 | EGF | P01133 | epidermal growth factor | DisGeNet |
| 176 | EGFR | P00533 | epidermal growth factor receptor | DisGeNet |
| 177 | EHMT1 | Q9H9B1 | euchromatic histone lysine methyltransferase 1 | DisGeNet |
| 178 | ELMO1 | Q92556 | engulfment and cell motility 1 | DisGeNet/OMIM |
| 179 | ENG | P17813 | endoglin | DisGeNet |
| 180 | ENPP1 | P22413 | ectonucleotide pyrophosphatase/phosphodiesterase 1 | DisGeNet/OMIM |
| 181 | ENTPD1 | P49961 | ectonucleoside triphosphate diphosphohydrolase 1 | DisGeNet |
| 182 | EPHX2 | P34913 | epoxide hydrolase 2 | DisGeNet/OMIM |
| 183 | EPO | P01588 | erythropoietin | DisGeNet/OMIM |
| 184 | ERBB4 | Q15303 | erb-b2 receptor tyrosine kinase 4 | DisGeNet |
| 185 | ERRFI1 | Q9UJM3 | ERBB receptor feedback inhibitor 1 | DisGeNet/OMIM |
| 186 | ESM1 | Q9NQ30 | endothelial cell specific molecule 1 | DisGeNet |
| 187 | ESR1 | P03372 | estrogen receptor 1 | DisGeNet/OMIM |
| 188 | ETS1 | P14921 | ETS proto-oncogene 1, transcription factor | DisGeNet |
| 189 | EZH2 | Q15910 | enhancer of zeste 2 polycomb repressive complex 2 subunit | DisGeNet/OMIM |
| 190 | F2 | P00734 | Prothrombin | OMIM |
| 191 | F5 | P12259 | coagulation factor V | DisGeNet/OMIM |
| 192 | FABP1 | P07148 | fatty acid binding protein 1 | DisGeNet |
| 193 | FABP4 | P15090 | fatty acid binding protein 4 | DisGeNet/OMIM |
| 194 | FASLG | P48023 | Fas ligand | DisGeNet |
| 195 | FCAR | P24071 | Immunoglobulin alpha Fc receptor | OMIM |
| 196 | FGF13 | Q92913 | fibroblast growth factor 13 | DisGeNet |
| 197 | FGF2 | P09038 | fibroblast growth factor 2 | DisGeNet |
| 198 | FGF21 | Q9NSA1 | fibroblast growth factor 21 | DisGeNet/OMIM |
| 199 | FHL2 | Q14192 | four and a half LIM domains 2 | DisGeNet |
| 200 | FLT1 | P17948 | fms related tyrosine kinase 1 | DisGeNet |
| 201 | FN1 | P02751 | fibronectin 1 | DisGeNet/CTD |
| 202 | FN3K | Q9H479 | fructosamine 3 kinase | DisGeNet |
| 203 | FNDC5 | Q8NAU1 | fibronectin type III domain containing 5 | DisGeNet |
| 204 | FOS | P01100 | Proto-oncogene c-Fos | CTD |
| 205 | FOXC1 | Q12948 | forkhead box C1 | DisGeNet |
| 206 | FOXP3 | Q9BZS1 | forkhead box P3 | DisGeNet/OMIM |
| 207 | FRMD3 | A2A2Y4 | FERM domain containing 3 | DisGeNet |
| 208 | G6PD | P11413 | glucose-6-phosphate dehydrogenase | DisGeNet |
| 209 | GABPA | Q06546 | GA binding protein transcription factor subunit alpha | DisGeNet |
| 210 | GAS6 | Q14393 | growth arrest specific 6 | DisGeNet |
| 211 | GCG | P01275 | glucagon | DisGeNet/OMIM |
| 212 | GCK | P35557 | glucokinase | DisGeNet/GeneCards/OMIM |
| 213 | GDF15 | Q99988 | growth differentiation factor 15 | DisGeNet |
| 214 | GFPT1 | Q06210 | glutamine--fructose-6-phosphate transaminase 1 | DisGeNet |
| 215 | GFPT2 | O94808 | glutamine-fructose-6-phosphate transaminase 2 | DisGeNet/OMIM |
| 216 | GH1 | P01241 | growth hormone 1 | DisGeNet |
| 217 | GHR | P10912 | growth hormone receptor | DisGeNet |
| 218 | GHRL | Q9UBU3 | ghrelin and obestatin prepropeptide | DisGeNet/OMIM |
| 219 | GIPR | P48546 | gastric inhibitory polypeptide receptor | DisGeNet/OMIM |
| 220 | GJA1 | P17302 | gap junction protein alpha 1 | DisGeNet |
| 221 | GLA | P06280 | Alpha-galactosidase A | OMIM |
| 222 | GLIPR2 | Q9H4G4 | GLI pathogenesis related 2 | DisGeNet |
| 223 | GLP1R | P43220 | glucagon like peptide 1 receptor | DisGeNet/OMIM |
| 224 | GORASP1 | Q9BQQ3 | golgi reassembly stacking protein 1 | DisGeNet |
| 225 | GPX1 | P07203 | glutathione peroxidase 1 | DisGeNet |
| 226 | GPX4 | P36969 | glutathione peroxidase 4 | DisGeNet |
| 227 | GRAP | Q13588 | GRB2 related adaptor protein | DisGeNet |
| 228 | GREM1 | O60565 | gremlin 1, DAN family BMP antagonist | DisGeNet |
| 229 | GSK3B | P49841 | glycogen synthase kinase 3 beta | DisGeNet/OMIM |
| 230 | GSR | P00390 | Glutathione reductase, mitochondrial | CTD |
| 231 | GSTM1 | P09488 | glutathione S-transferase mu 1 | DisGeNet |
| 232 | GTF2H1 | P32780 | general transcription factor IIH subunit 1 | DisGeNet |
| 233 | HAVCR1 | Q96D42 | hepatitis A virus cellular receptor 1 | DisGeNet |
| 234 | HBA1 | P69905 | hemoglobin subunit alpha 1 | DisGeNet/OMIM |
| 235 | HBB | P68871 | Hemoglobin subunit beta | OMIM |
| 236 | HCCS | P53701 | holocytochrome c synthase | DisGeNet |
| 237 | HCN1 | O60741 | hyperpolarization activated cyclic nucleotide gated potassium channel 1 | DisGeNet |
| 238 | HDAC4 | P56524 | histone deacetylase 4 | DisGeNet |
| 239 | HES1 | Q14469 | hes family bHLH transcription factor 1 | DisGeNet |
| 240 | HFE | Q30201 | homeostatic iron regulator | DisGeNet/OMIM |
| 241 | HGF | P14210 | hepatocyte growth factor | DisGeNet |
| 242 | HHEX | Q03014 | hematopoietically expressed homeobox | DisGeNet/OMIM |
| 243 | HIF1A | Q16665 | hypoxia inducible factor 1 subunit alpha | DisGeNet/OMIM |
| 244 | HLA-DQA1 | P01909 | HLA class II histocompatibility antigen, DQ alpha 1 chain | OMIM |
| 245 | HLA-DRB1 | P01911;P01912;P04229;P13760;P13761;P20039;Q29974;Q30134;Q30167;Q5Y7A7;Q95IE3;Q9GIY3;Q9TQE0 | major histocompatibility complex, class II, DR beta 1 | DisGeNet/OMIM |
| 246 | HMCN1 | Q96RW7 | hemicentin 1 | DisGeNet |
| 247 | HMGA2 | P52926 | high mobility group AT-hook 2 | DisGeNet/OMIM |
| 248 | HMGB1 | P09429 | high mobility group box 1 | DisGeNet |
| 249 | HNF1A | P20823 | HNF1 homeobox A | DisGeNet/GeneCards/OMIM |
| 250 | HNF1B | P35680 | HNF1 homeobox B | DisGeNet/GeneCards/OMIM |
| 251 | HNF4A | P41235 | hepatocyte nuclear factor 4 alpha | DisGeNet/GeneCards/OMIM |
| 252 | HP | P00738 | haptoglobin | DisGeNet/OMIM |
| 253 | HPGDS | O60760 | hematopoietic prostaglandin D synthase | DisGeNet |
| 254 | HPSE | Q9Y251 | heparanase | DisGeNet |
| 255 | HSD11B2 | P80365 | hydroxysteroid 11-beta dehydrogenase 2 | DisGeNet |
| 256 | HSPA1A | P0DMV8;P0DMV9 | heat shock protein family A (Hsp70) member 1A | DisGeNet |
| 257 | HSPA1B | P0DMV8;P0DMV9 | heat shock protein family A (Hsp70) member 1B | DisGeNet |
| 258 | HSPA4 | P34932 | heat shock protein family A (Hsp70) member 4 | DisGeNet |
| 259 | HSPA5 | P11021 | heat shock protein family A (Hsp70) member 5 | DisGeNet |
| 260 | HSPG2 | P98160 | heparan sulfate proteoglycan 2 | DisGeNet |
| 261 | HTR2A | P28223 | 5-hydroxytryptamine receptor 2A | DisGeNet |
| 262 | HYOU1 | Q9Y4L1 | hypoxia up-regulated 1 | DisGeNet/OMIM |
| 263 | IAPP | P10997 | islet amyloid polypeptide | DisGeNet/OMIM |
| 264 | ICAM1 | P05362 | intercellular adhesion molecule 1 | DisGeNet/CTD |
| 265 | ID2 | Q02363 | inhibitor of DNA binding 2 | DisGeNet |
| 266 | IDE | P14735 | insulin degrading enzyme | DisGeNet/OMIM |
| 267 | IDUA | P35475 | iduronidase, alpha-L- | DisGeNet |
| 268 | IFNG | P01579 | Interferon gamma | CTD |
| 269 | IGF1 | P05019 | insulin like growth factor 1 | DisGeNet/OMIM |
| 270 | IGF2 | P01344 | insulin like growth factor 2 | DisGeNet/OMIM |
| 271 | IGF2BP2 | Q9Y6M1 | insulin like growth factor 2 mRNA binding protein 2 | DisGeNet/OMIM |
| 272 | IGHA1 | P01876 | Immunoglobulin heavy constant alpha 1 | OMIM |
| 273 | IL10 | P22301 | interleukin 10 | DisGeNet/OMIM |
| 274 | IL15 | P40933 | interleukin 15 | DisGeNet |
| 275 | IL17A | Q16552 | interleukin 17A | DisGeNet/OMIM |
| 276 | IL17B | Q9UHF5 | interleukin 17B | DisGeNet |
| 277 | IL18 | Q14116 | interleukin 18 | DisGeNet/OMIM |
| 278 | IL18R1 | Q13478 | interleukin 18 receptor 1 | DisGeNet |
| 279 | IL1A | P01583 | interleukin 1 alpha | DisGeNet/OMIM |
| 280 | IL1B | P01584 | interleukin 1 beta | DisGeNet/CTD/OMIM |
| 281 | IL1RN | P18510 | interleukin 1 receptor antagonist | DisGeNet/OMIM |
| 282 | IL20 | Q9NYY1 | interleukin 20 | DisGeNet |
| 283 | IL6 | P05231 | interleukin 6 | DisGeNet/GeneCards/CTD/OMIM |
| 284 | IL6R | P08887 | interleukin 6 receptor | DisGeNet/OMIM |
| 285 | INPPL1 | O15357 | inositol polyphosphate phosphatase like 1 | DisGeNet/OMIM |
| 286 | INS | P01308 | insulin | DisGeNet/GeneCards/OMIM |
| 287 | INSR | P06213 | insulin receptor | DisGeNet/OMIM |
| 288 | IPCEF1 | Q8WWN9 | interaction protein for cytohesin exchange factors 1 | DisGeNet |
| 289 | IQGAP1 | P46940 | IQ motif containing GTPase activating protein 1 | DisGeNet |
| 290 | IRS2 | Q9Y4H2 | insulin receptor substrate 2 | DisGeNet/OMIM |
| 291 | ISYNA1 | Q9NPH2 | inositol-3-phosphate synthase 1 | DisGeNet |
| 292 | ITGA1 | P56199 | integrin subunit alpha 1 | DisGeNet |
| 293 | ITGB3 | P05106 | integrin subunit beta 3 | DisGeNet/OMIM |
| 294 | JAG1 | P78504 | jagged 1 | DisGeNet |
| 295 | JAK1 | P23458 | Janus kinase 1 | DisGeNet |
| 296 | JAK2 | O60674 | Janus kinase 2 | DisGeNet |
| 297 | JUN | P05412 | Jun proto-oncogene, AP-1 transcription factor subunit | DisGeNet/DrugBank |
| 298 | KCNQ1 | P51787 | potassium voltage-gated channel subfamily Q member 1 | DisGeNet |
| 299 | KDR | P35968 | kinase insert domain receptor | DisGeNet/OMIM |
| 300 | KHDRBS1 | Q07666 | KH RNA binding domain containing, signal transduction associated 1 | DisGeNet |
| 301 | KIRREL1 | Q96J84 | kirre like nephrin family adhesion molecule 1 | DisGeNet |
| 302 | KL | Q9UEF7 | klotho | DisGeNet |
| 303 | KLF2 | Q9Y5W3 | Kruppel like factor 2 | DisGeNet |
| 304 | KLF6 | Q99612 | Kruppel like factor 6 | DisGeNet |
| 305 | KLK1 | P06870 | kallikrein 1 | DisGeNet |
| 306 | KMT2A | Q03164 | lysine methyltransferase 2A | DisGeNet |
| 307 | KNG1 | P01042 | kininogen 1 | DisGeNet |
| 308 | KRT16 | P08779 | keratin 16 | DisGeNet |
| 309 | LAMB2 | P55268 | laminin subunit beta 2 | DisGeNet |
| 310 | LCN2 | P80188 | lipocalin 2 | DisGeNet |
| 311 | LEP | P41159 | leptin | DisGeNet/GeneCards/OMIM |
| 312 | LGALS3 | P17931 | galectin 3 | DisGeNet |
| 313 | LIMK2 | P53671 | LIM domain kinase 2 | DisGeNet |
| 314 | LIN28A | Q9H9Z2 | lin-28 homolog A | DisGeNet |
| 315 | LIPC | P11150 | lipase C, hepatic type | DisGeNet/OMIM |
| 316 | LMNA | P02545 | lamin A/C | DisGeNet/OMIM |
| 317 | LPA | P08519 | lipoprotein(a) | DisGeNet |
| 318 | LPL | P06858 | lipoprotein lipase | DisGeNet/OMIM |
| 319 | LRP5 | O75197 | LDL receptor related protein 5 | DisGeNet/OMIM |
| 320 | LRP6 | O75581 | LDL receptor related protein 6 | DisGeNet/OMIM |
| 321 | LRRC7 | Q96NW7 | leucine rich repeat containing 7 | DisGeNet |
| 322 | LTA | P01374 | lymphotoxin alpha | DisGeNet |
| 323 | LTA4H | P09960 | Leukotriene A-4 hydrolase | DrugBank |
| 324 | LTBP1 | Q14766 | latent transforming growth factor beta binding protein 1 | DisGeNet |
| 325 | LYZ | P61626 | lysozyme | DisGeNet/OMIM |
| 326 | MAP2K2 | P36507 | mitogen-activated protein kinase kinase 2 | DisGeNet |
| 327 | MAP3K5 | Q99683 | Mitogen-activated protein kinase kinase kinase 5 | TTD |
| 328 | MAPK1 | P28482 | mitogen-activated protein kinase 1 | DisGeNet/CTD |
| 329 | MAPK12 | P53778 | MAP kinase p38 | TTD |
| 330 | MAPK14 | Q16539 | mitogen-activated protein kinase 14 | DisGeNet/OMIM |
| 331 | MAPK3 | P27361 | mitogen-activated protein kinase 3 | DisGeNet/CTD/OMIM |
| 332 | MAPK8 | P45983 | mitogen-activated protein kinase 8 | DisGeNet/CTD/OMIM |
| 333 | MARK2 | Q7KZI7 | microtubule affinity regulating kinase 2 | DisGeNet |
| 334 | MBL2 | P11226 | mannose binding lectin 2 | DisGeNet/OMIM |
| 335 | MCAM | P43121 | melanoma cell adhesion molecule | DisGeNet |
| 336 | MDK | P21741 | midkine | DisGeNet |
| 337 | MEP1B | Q16820 | meprin A subunit beta | DisGeNet |
| 338 | MGP | P08493 | matrix Gla protein | DisGeNet |
| 339 | MIF | P14174 | macrophage migration inhibitory factor | DisGeNet |
| 340 | MKL1 | Q969V6 | megakaryoblastic leukemia (translocation) 1 | DisGeNet |
| 341 | MLXIPL | Q9NP71 | MLX interacting protein like | DisGeNet/OMIM |
| 342 | MLYCD | O95822 | malonyl-CoA decarboxylase | DisGeNet |
| 343 | MMP1 | P03956 | Interstitial collagenase | TTD |
| 344 | MMP10 | P09238 | matrix metallopeptidase 10 | DisGeNet |
| 345 | MMP12 | P39900 | matrix metallopeptidase 12 | DisGeNet/OMIM |
| 346 | MMP2 | P08253 | matrix metallopeptidase 2 | DisGeNet/CTD/DrugBank/OMIM |
| 347 | MMP24 | Q9Y5R2 | matrix metallopeptidase 24 | DisGeNet |
| 348 | MMP3 | P08254 | matrix metallopeptidase 3 | DisGeNet |
| 349 | MMP7 | P09237 | matrix metallopeptidase 7 | DisGeNet |
| 350 | MMP8 | P22894 | matrix metallopeptidase 8 | DisGeNet |
| 351 | MMP9 | P14780 | matrix metallopeptidase 9 | DisGeNet/CTD/DrugBank |
| 352 | MMRN1 | Q13201 | multimerin 1 | DisGeNet |
| 353 | MOK | Q9UQ07 | MOK protein kinase | DisGeNet |
| 354 | MPO | P05164 | myeloperoxidase | DisGeNet/CTD |
| 355 | MTG1 | Q9BT17 | mitochondrial ribosome associated GTPase 1 | DisGeNet |
| 356 | MTHFR | P42898 | methylenetetrahydrofolate reductase | DisGeNet/DrugBank |
| 357 | MUC1 | P15941 | mucin 1, cell surface associated | DisGeNet |
| 358 | MYH9 | P35579 | myosin heavy chain 9 | DisGeNet/OMIM |
| 359 | NAT2 | P11245 | N-acetyltransferase 2 | DisGeNet |
| 360 | NCALD | P61601 | neurocalcin delta | DisGeNet |
| 361 | NCF1 | P14598 | neutrophil cytosolic factor 1 | DisGeNet |
| 362 | NCK1 | P16333 | NCK adaptor protein 1 | DisGeNet |
| 363 | NEDD4L | Q96PU5 | E3 ubiquitin-protein ligase NEDD4-like | OMIM |
| 364 | NELFE | P18615 | negative elongation factor complex member E | DisGeNet |
| 365 | NFAT5 | O94916 | nuclear factor of activated T cells 5 | DisGeNet |
| 366 | NFE2L2 | Q16236 | nuclear factor, erythroid 2 like 2 | DisGeNet/CTD |
| 367 | NFKB1 | P19838 | nuclear factor kappa B subunit 1 | DisGeNet/OMIM |
| 368 | NFKBIA | P25963 | NF-kappa-B inhibitor alpha | CTD |
| 369 | NLRP3 | Q96P20 | NLR family pyrin domain containing 3 | DisGeNet/OMIM |
| 370 | NOD2 | Q9HC29 | nucleotide binding oligomerization domain containing 2 | DisGeNet/OMIM |
| 371 | NOS1 | P29475 | nitric oxide synthase 1 | DisGeNet |
| 372 | NOS3 | P29474 | nitric oxide synthase 3 | DisGeNet/CTD |
| 373 | NOTCH1 | P46531 | Neurogenic locus notch homolog protein 1 | OMIM |
| 374 | NOTCH3 | Q9UM47 | notch 3 | DisGeNet/OMIM |
| 375 | NOX1 | Q9Y5S8 | NADPH oxidase 1 | DisGeNet |
| 376 | NOX3 | Q9HBY0 | NADPH oxidase 3 | DisGeNet |
| 377 | NOX4 | Q9NPH5 | NADPH oxidase 4 | DisGeNet |
| 378 | NOX5 | Q96PH1 | NADPH oxidase 5 | DisGeNet |
| 379 | NPHP3 | Q7Z494 | Nephrocystin-3 | OMIM |
| 380 | NPHS1 | O60500 | NPHS1, nephrin | DisGeNet |
| 381 | NPHS2 | Q9NP85 | NPHS2, podocin | DisGeNet |
| 382 | NPPA | P01160 | natriuretic peptide A | DisGeNet/OMIM |
| 383 | NPPB | P16860 | natriuretic peptide B | DisGeNet |
| 384 | NPY | P01303 | neuropeptide Y | DisGeNet/OMIM |
| 385 | NR0B2 | Q15466 | nuclear receptor subfamily 0 group B member 2 | DisGeNet/OMIM |
| 386 | NR1H3 | Q13133 | nuclear receptor subfamily 1 group H member 3 | DisGeNet |
| 387 | NR1H4 | Q96RI1 | nuclear receptor subfamily 1 group H member 4 | DisGeNet/OMIM |
| 388 | NR3C2 | P08235 | nuclear receptor subfamily 3 group C member 2 | DisGeNet/TTD |
| 389 | NSA2 | O95478 | NSA2, ribosome biogenesis homolog | DisGeNet |
| 390 | NTRK1 | P04629 | neurotrophic receptor tyrosine kinase 1 | DisGeNet |
| 391 | NUAK1 | O60285 | NUAK family kinase 1 | DisGeNet |
| 392 | NUP62 | P37198 | nucleoporin 62 | DisGeNet |
| 393 | ORM1 | P02763 | Alpha-1-acid glycoprotein 1 | DrugBank |
| 394 | ORM2 | P19652 | Alpha-1-acid glycoprotein 2 | DrugBank |
| 395 | PACSIN2 | Q9UNF0 | protein kinase C and casein kinase substrate in neurons 2 | DisGeNet |
| 396 | PCSK1 | P29120 | proprotein convertase subtilisin/kexin type 1 | DisGeNet/OMIM |
| 397 | PDGFA | P04085 | platelet derived growth factor subunit A | DisGeNet |
| 398 | PEA15 | Q15121 | Astrocytic phosphoprotein PEA-15 | OMIM |
| 399 | PFKFB2 | O60825 | 6-phosphofructo-2-kinase/fructose-2,6-biphosphatase 2 | DisGeNet |
| 400 | PICK1 | Q9NRD5 | protein interacting with PRKCA 1 | DisGeNet |
| 401 | PIGR | P01833 | Polymeric immunoglobulin receptor | OMIM |
| 402 | PIK3CA | P42336 | phosphatidylinositol-4,5-bisphosphate 3-kinase catalytic subunit alpha | DisGeNet |
| 403 | PIK3CB | P42338 | phosphatidylinositol-4,5-bisphosphate 3-kinase catalytic subunit beta | DisGeNet/OMIM |
| 404 | PIK3CD | O00329 | phosphatidylinositol-4,5-bisphosphate 3-kinase catalytic subunit delta | DisGeNet |
| 405 | PIK3CG | P48736 | phosphatidylinositol-4,5-bisphosphate 3-kinase catalytic subunit gamma | DisGeNet |
| 406 | PIK3R1 | P27986 | phosphoinositide-3-kinase regulatory subunit 1 | DisGeNet/OMIM |
| 407 | PIK3R2 | O00459 | phosphoinositide-3-kinase regulatory subunit 2 | DisGeNet |
| 408 | PITX2 | Q99697 | paired like homeodomain 2 | DisGeNet |
| 409 | PKD1 | P98161 | polycystin 1, transient receptor potential channel interacting | DisGeNet |
| 410 | PLA2R1 | Q13018 | Secretory phospholipase A2 receptor | OMIM |
| 411 | PLAUR | Q03405 | Urokinase plasminogen activator surface receptor | OMIM |
| 412 | PLEKHH2 | Q8IVE3 | pleckstrin homology, MyTH4 and FERM domain containing H2 | DisGeNet/OMIM |
| 413 | PLEKHO1 | Q53GL0 | pleckstrin homology domain containing O1 | DisGeNet |
| 414 | PLG | P00747 | plasminogen | DisGeNet |
| 415 | PNO1 | Q9NRX1 | partner of NOB1 homolog | DisGeNet |
| 416 | PNPLA2 | Q96AD5 | patatin like phospholipase domain containing 2 | DisGeNet/OMIM |
| 417 | PON1 | P27169 | paraoxonase 1 | DisGeNet |
| 418 | PON2 | Q15165 | Serum paraoxonase/arylesterase 2 | OMIM |
| 419 | PPARA | Q07869 | peroxisome proliferator activated receptor alpha | DisGeNet |
| 420 | PPARD | Q03181 | peroxisome proliferator activated receptor delta | DisGeNet/OMIM |
| 421 | PPARG | P37231 | peroxisome proliferator activated receptor gamma | DisGeNet/GeneCards/CTD/DrugBank/OMIM |
| 422 | PPARGC1A | Q9UBK2 | PPARG coactivator 1 alpha | DisGeNet/CTD/OMIM |
| 423 | PPIG | Q13427 | peptidylprolyl isomerase G | DisGeNet |
| 424 | PPP1R8 | Q12972 | protein phosphatase 1 regulatory subunit 8 | DisGeNet |
| 425 | PPP3R1 | P63098 | Calcineurin subunit B type 1 | OMIM |
| 426 | PRH2 | P02810 | proline rich protein HaeIII subfamily 2 | DisGeNet |
| 427 | PRKCA | P17252 | protein kinase C alpha | DisGeNet |
| 428 | PRKCB | P05771 | protein kinase C beta | DisGeNet |
| 429 | PRKCE | Q02156 | protein kinase C epsilon | DisGeNet |
| 430 | PRKCSH | P14314 | protein kinase C substrate 80K-H | DisGeNet |
| 431 | PRMT1 | Q99873 | protein arginine methyltransferase 1 | DisGeNet |
| 432 | PROC | P04070 | protein C, inactivator of coagulation factors Va and VIIIa | DisGeNet/OMIM |
| 433 | PRSS1 | P07477 | serine protease 1 | DisGeNet |
| 434 | PRSS2 | P07478 | serine protease 2 | DisGeNet |
| 435 | PRSS55 | Q6UWB4 | serine protease 55 | DisGeNet |
| 436 | PSMD9 | O00233 | proteasome 26S subunit, non-ATPase 9 | DisGeNet |
| 437 | PTBP1 | P26599 | polypyrimidine tract binding protein 1 | DisGeNet |
| 438 | PTEN | P60484 | phosphatase and tensin homolog | DisGeNet/OMIM |
| 439 | PTGDS | P41222 | prostaglandin D2 synthase | DisGeNet/OMIM |
| 440 | PTGS1 | P23219 | prostaglandin-endoperoxide synthase 1 | DisGeNet/DrugBank |
| 441 | PTGS2 | P35354 | prostaglandin-endoperoxide synthase 2 | DisGeNet/CTD/OMIM |
| 442 | PTH | P01270 | parathyroid hormone | DisGeNet |
| 443 | PTHLH | P12272 | parathyroid hormone like hormone | DisGeNet |
| 444 | PTK2B | Q14289 | protein tyrosine kinase 2 beta | DisGeNet |
| 445 | PTPN1 | P18031 | protein tyrosine phosphatase, non-receptor type 1 | DisGeNet/OMIM |
| 446 | PTPN6 | P29350 | protein tyrosine phosphatase, non-receptor type 6 | DisGeNet/OMIM |
| 447 | RAB38 | P57729 | RAB38, member RAS oncogene family | DisGeNet |
| 448 | RAMP2 | O60895 | receptor activity modifying protein 2 | DisGeNet |
| 449 | RAPGEF5 | Q92565 | Rap guanine nucleotide exchange factor 5 | DisGeNet |
| 450 | RB1CC1 | Q8TDY2 | RB1 inducible coiled-coil 1 | DisGeNet |
| 451 | RBMS3 | Q6XE24 | RNA binding motif single stranded interacting protein 3 | DisGeNet |
| 452 | RBP4 | P02753 | retinol binding protein 4 | DisGeNet/OMIM |
| 453 | RELA | Q04206 | RELA proto-oncogene, NF-kB subunit | DisGeNet/CTD |
| 454 | REM1 | O75628 | RRAD and GEM like GTPase 1 | DisGeNet |
| 455 | REN | P00797 | renin | DisGeNet/GeneCards/DrugBank/OMIM |
| 456 | RENBP | P51606 | renin binding protein | DisGeNet |
| 457 | RIMS2 | Q9UQ26 | regulating synaptic membrane exocytosis 2 | DisGeNet |
| 458 | RMDN1 | Q96DB5 | regulator of microtubule dynamics 1 | DisGeNet |
| 459 | RMDN2 | Q96LZ7 | regulator of microtubule dynamics 2 | DisGeNet |
| 460 | RMDN3 | Q96TC7 | regulator of microtubule dynamics 3 | DisGeNet |
| 461 | ROCK1 | Q13464 | Rho associated coiled-coil containing protein kinase 1 | DisGeNet/OMIM/TTD |
| 462 | ROS1 | P08922 | ROS proto-oncogene 1, receptor tyrosine kinase | DisGeNet |
| 463 | RPL36A | P83881 | ribosomal protein L36a | DisGeNet |
| 464 | RPS19 | P39019 | ribosomal protein S19 | DisGeNet |
| 465 | RRAD | P55042 | GTP-binding protein RAD | OMIM |
| 466 | S100A8 | P05109 | S100 calcium binding protein A8 | DisGeNet |
| 467 | S100A9 | P06702 | S100 calcium binding protein A9 | DisGeNet |
| 468 | SAA1 | P0DJI8 | serum amyloid A1 | DisGeNet |
| 469 | SASH1 | O94885 | SAM and SH3 domain containing 1 | DisGeNet |
| 470 | SCAF4 | O95104 | SR-related CTD associated factor 4 | DisGeNet |
| 471 | SCAF8 | Q9UPN6 | SR-related CTD associated factor 8 | DisGeNet |
| 472 | SCD | O00767 | stearoyl-CoA desaturase | DisGeNet/OMIM |
| 473 | SCNN1G | P51170 | sodium channel epithelial 1 gamma subunit | DisGeNet/OMIM |
| 474 | SDC2 | P34741 | syndecan 2 | DisGeNet |
| 475 | SEC61A1 | P61619 | Protein transport protein Sec61 subunit alpha isoform 1 | OMIM |
| 476 | SELE | P16581 | E-selectin | OMIM |
| 477 | SELENBP1 | Q13228 | selenium binding protein 1 | DisGeNet |
| 478 | SELL | P14151 | selectin L | DisGeNet/OMIM |
| 479 | SELP | P16109 | selectin P | DisGeNet/OMIM |
| 480 | SEMA3A | Q14563 | semaphorin 3A | DisGeNet |
| 481 | SEMA6A | Q9H2E6 | semaphorin 6A | DisGeNet |
| 482 | SERPINB2 | P05120 | serpin family B member 2 | DisGeNet |
| 483 | SERPINB7 | O75635 | Serpin B7 (Megsin) (TP55) | OMIM |
| 484 | SERPINE1 | P05121 | serpin family E member 1 | DisGeNet/CTD/OMIM |
| 485 | SFI1 | A8K8P3 | SFI1 centrin binding protein | DisGeNet |
| 486 | SFRP4 | Q6FHJ7 | Secreted frizzled-related protein 4 | DrugBank |
| 487 | SGK1 | O00141 | serum/glucocorticoid regulated kinase 1 | DisGeNet/OMIM |
| 488 | SHBG | P04278 | sex hormone binding globulin | DisGeNet/OMIM |
| 489 | SIRT1 | Q96EB6 | sirtuin 1 | DisGeNet/OMIM |
| 490 | SLC12A3 | P55017 | solute carrier family 12 member 3 | DisGeNet |
| 491 | SLC15A1 | P46059 | Solute carrier family 15 member 1 | DrugBank |
| 492 | SLC15A2 | Q16348 | Solute carrier family 15 member 2 | DrugBank |
| 493 | SLC22A12 | Q96S37 | Solute carrier family 22 member 12 | DrugBank |
| 494 | SLC22A2 | O15244 | solute carrier family 22 member 2 | DisGeNet |
| 495 | SLC22A3 | O75751 | solute carrier family 22 member 3 | DisGeNet |
| 496 | SLC22A6 | Q4U2R8 | solute carrier family 22 member 6 | DisGeNet/DrugBank |
| 497 | SLC22A7 | Q9Y694 | Solute carrier family 22 member 7 | DrugBank |
| 498 | SLC22A8 | Q8TCC7 | solute carrier family 22 member 8 | DisGeNet/DrugBank |
| 499 | SLC2A1 | P11166 | solute carrier family 2 member 1 | DisGeNet/OMIM |
| 500 | SLC2A12 | Q8TD20 | solute carrier family 2 member 12 | DisGeNet/OMIM |
| 501 | SLC2A2 | P11168 | solute carrier family 2 member 2 | DisGeNet/OMIM |
| 502 | SLC2A9 | Q9NRM0 | Solute carrier family 2, facilitated glucose transporter member 9 | DrugBank/OMIM |
| 503 | SLC33A1 | O00400 | solute carrier family 33 member 1 | DisGeNet |
| 504 | SLC5A2 | P31639 | solute carrier family 5 member 2 | DisGeNet/DrugBank/OMIM |
| 505 | SLC5A4 | Q9NY91 | solute carrier family 5 member 4 | DisGeNet |
| 506 | SLC9C1 | Q4G0N8 | solute carrier family 9 member C1 | DisGeNet |
| 507 | SLCO1A2 | P46721 | Solute carrier organic anion transporter family member 1A2 | DrugBank |
| 508 | SLCO1B1 | Q9Y6L6 | Solute carrier organic anion transporter family member 1B1 | DrugBank |
| 509 | SLCO1B3 | Q9NPD5 | Solute carrier organic anion transporter family member 1B3 | DrugBank |
| 510 | SLPI | P03973 | secretory leukocyte peptidase inhibitor | DisGeNet |
| 511 | SMAD1 | Q15797 | SMAD family member 1 | DisGeNet |
| 512 | SMAD5 | Q99717 | SMAD family member 5 | DisGeNet |
| 513 | SMAD7 | O15105 | SMAD family member 7 | DisGeNet/OMIM |
| 514 | SMN1 | Q16637 | survival of motor neuron 1, telomeric | DisGeNet |
| 515 | SMN2 | Q16637 | survival of motor neuron 2, centromeric | DisGeNet |
| 516 | SMURF2 | Q9HAU4 | SMAD specific E3 ubiquitin protein ligase 2 | DisGeNet |
| 517 | SNAI1 | O95863 | snail family transcriptional repressor 1 | DisGeNet |
| 518 | SNRNP70 | P08621 | small nuclear ribonucleoprotein U1 subunit 70 | DisGeNet |
| 519 | SOD1 | P00441 | superoxide dismutase 1 | DisGeNet/CTD |
| 520 | SOD2 | P04179 | superoxide dismutase 2 | DisGeNet/CTD/OMIM |
| 521 | SORBS1 | Q9BX66 | sorbin and SH3 domain containing 1 | DisGeNet/OMIM |
| 522 | SORD | Q00796 | sorbitol dehydrogenase | DisGeNet/OMIM |
| 523 | SOS1 | Q07889 | SOS Ras/Rac guanine nucleotide exchange factor 1 | DisGeNet |
| 524 | SPARC | P09486 | secreted protein acidic and cysteine rich | DisGeNet |
| 525 | SPHK1 | Q9NYA1 | sphingosine kinase 1 | DisGeNet |
| 526 | SPINK1 | P00995 | serine peptidase inhibitor, Kazal type 1 | DisGeNet/OMIM |
| 527 | SPP1 | P10451 | secreted phosphoprotein 1 | DisGeNet |
| 528 | SPRY2 | O43597 | Protein sprouty homolog 2 | OMIM |
| 529 | SPZ1 | Q9BXG8 | spermatogenic leucine zipper 1 | DisGeNet |
| 530 | SQSTM1 | Q13501 | sequestosome 1 | DisGeNet/OMIM |
| 531 | SRRM2 | Q9UQ35 | serine/arginine repetitive matrix 2 | DisGeNet |
| 532 | ST3GAL4 | Q11206 | ST3 beta-galactoside alpha-2,3-sialyltransferase 4 | DisGeNet |
| 533 | STAT1 | P42224 | signal transducer and activator of transcription 1 | DisGeNet |
| 534 | STAT3 | P40763 | signal transducer and activator of transcription 3 | DisGeNet/OMIM |
| 535 | STAT5A | P42229 | signal transducer and activator of transcription 5A | DisGeNet/OMIM |
| 536 | STAT5B | P51692 | signal transducer and activator of transcription 5B | DisGeNet |
| 537 | SUMO4 | Q6EEV6 | small ubiquitin-like modifier 4 | DisGeNet/OMIM |
| 538 | SYBU | Q9NX95 | syntabulin | DisGeNet |
| 539 | SYK | P43405 | spleen associated tyrosine kinase | DisGeNet |
| 540 | SYT1 | P21579 | synaptotagmin 1 | DisGeNet |
| 541 | TALDO1 | P37837 | transaldolase 1 | DisGeNet |
| 542 | TBC1D31 | Q96DN5 | TBC1 domain family member 31 | DisGeNet |
| 543 | TCF7L2 | Q9NQB0 | transcription factor 7 like 2 | DisGeNet/OMIM |
| 544 | TFG | Q92734 | TRK-fused gene | DisGeNet |
| 545 | TFPI | P10646 | tissue factor pathway inhibitor | DisGeNet |
| 546 | TGFA | P01135 | transforming growth factor alpha | DisGeNet |
| 547 | TGFB1 | P01137 | transforming growth factor beta 1 | DisGeNet/CTD/OMIM |
| 548 | TGFB2 | P61812 | transforming growth factor beta 2 | DisGeNet |
| 549 | TGFB3 | P10600 | transforming growth factor beta 3 | DisGeNet |
| 550 | TGFBI | Q15582 | transforming growth factor beta induced | DisGeNet |
| 551 | TGFBR1 | P36897 | transforming growth factor beta receptor 1 | DisGeNet/TTD |
| 552 | TGFBR2 | P37173 | transforming growth factor beta receptor 2 | DisGeNet |
| 553 | THBD | P07204 | thrombomodulin | DisGeNet |
| 554 | THBS1 | P07996 | thrombospondin 1 | DisGeNet |
| 555 | THG1L | Q9NWX6 | tRNA-histidine guanylyltransferase 1 like | DisGeNet |
| 556 | TIMM44 | O43615 | translocase of inner mitochondrial membrane 44 | DisGeNet |
| 557 | TIMP1 | P01033 | TIMP metallopeptidase inhibitor 1 | DisGeNet |
| 558 | TIMP2 | P16035 | TIMP metallopeptidase inhibitor 2 | DisGeNet/OMIM |
| 559 | TIMP3 | P35625 | TIMP metallopeptidase inhibitor 3 | DisGeNet |
| 560 | TINAG | Q9UJW2 | tubulointerstitial nephritis antigen | DisGeNet |
| 561 | TKT | P29401 | transketolase | DisGeNet |
| 562 | TLR2 | O60603 | toll like receptor 2 | DisGeNet |
| 563 | TLR4 | O00206 | toll like receptor 4 | DisGeNet |
| 564 | TNF | P01375 | tumor necrosis factor | DisGeNet/CTD/OMIM |
| 565 | TNFAIP1 | Q13829 | TNF alpha induced protein 1 | DisGeNet |
| 566 | TNFAIP8 | O95379 | TNF alpha induced protein 8 | DisGeNet |
| 567 | TNFRSF11B | O00300 | TNF receptor superfamily member 11b | DisGeNet |
| 568 | TNFSF10 | P50591 | TNF superfamily member 10 | DisGeNet/OMIM |
| 569 | TP53INP2 | Q8IXH6 | Tumor protein p53-inducible nuclear protein 2 | OMIM |
| 570 | TPD52 | P55327 | tumor protein D52 | DisGeNet |
| 571 | TRDN | Q13061 | triadin | DisGeNet |
| 572 | TRPC1 | P48995 | transient receptor potential cation channel subfamily C member 1 | DisGeNet |
| 573 | TRPC6 | Q9Y210 | transient receptor potential cation channel subfamily C member 6 | DisGeNet |
| 574 | TSC22D1 | Q15714 | TSC22 domain family member 1 | DisGeNet |
| 575 | TSPYL2 | Q9H2G4 | TSPY like 2 | DisGeNet |
| 576 | TXN | P10599 | thioredoxin | DisGeNet |
| 577 | TXNIP | Q9H3M7 | thioredoxin interacting protein | DisGeNet |
| 578 | UCP1 | P25874 | uncoupling protein 1 | DisGeNet |
| 579 | UCP2 | P55851 | uncoupling protein 2 | DisGeNet/OMIM |
| 580 | UCP3 | P55916 | uncoupling protein 3 | DisGeNet/OMIM |
| 581 | UGT1A1 | P22309 | UDP-glucuronosyltransferase 1-1 | DrugBank/OMIM |
| 582 | UGT1A10 | Q9HAW8 | UDP-glucuronosyltransferase 1-10 | DrugBank |
| 583 | UGT1A3 | P35503 | UDP-glucuronosyltransferase 1-3 | DrugBank |
| 584 | UGT1A9 | O60656 | UDP-glucuronosyltransferase 1-9 | DrugBank |
| 585 | UGT2B17 | O75795 | UDP-glucuronosyltransferase 2B17 | DrugBank |
| 586 | UGT2B4 | P06133 | UDP-glucuronosyltransferase 2B4 | DrugBank |
| 587 | UGT2B7 | P16662 | UDP-glucuronosyltransferase 2B7 | DrugBank |
| 588 | UMOD | P07911 | uromodulin | DisGeNet/OMIM |
| 589 | UNC13B | O14795 | unc-13 homolog B | DisGeNet/OMIM |
| 590 | UTRN | P46939 | utrophin | DisGeNet |
| 591 | UTS2 | O95399 | urotensin 2 | DisGeNet |
| 592 | UTS2R | Q9UKP6 | urotensin 2 receptor | DisGeNet/TTD |
| 593 | VASH1 | Q7L8A9 | vasohibin 1 | DisGeNet |
| 594 | VCAM1 | P19320 | Vascular cell adhesion protein 1 | CTD |
| 595 | VCAN | P13611 | versican | DisGeNet |
| 596 | VDAC1 | P21796 | voltage dependent anion channel 1 | DisGeNet |
| 597 | VDAC2 | P45880 | voltage dependent anion channel 2 | DisGeNet |
| 598 | VDR | P11473 | vitamin D receptor | DisGeNet/OMIM |
| 599 | VEGFA | P15692 | vascular endothelial growth factor A | DisGeNet/GeneCards/CTD/OMIM |
| 600 | VPS51 | Q9UID3 | VPS51, GARP complex subunit | DisGeNet |
| 601 | VWF | P04275 | von Willebrand factor | DisGeNet |
| 602 | WFS1 | O76024 | wolframin ER transmembrane glycoprotein | DisGeNet/GeneCards/OMIM |
| 603 | WNK1 | Q9H4A3 | WNK lysine deficient protein kinase 1 | DisGeNet |
| 604 | WT1 | P19544 | Wilms tumor 1 | DisGeNet/OMIM |
| 605 | XBP1 | P17861 | X-box binding protein 1 | DisGeNet/OMIM |
| 606 | XYLT1 | Q86Y38 | xylosyltransferase 1 | DisGeNet |
| 607 | XYLT2 | Q9H1B5 | xylosyltransferase 2 | DisGeNet |
| 608 | ZEB1 | P37275 | Zinc finger E-box-binding homeobox 1 | OMIM |
| 609 | ZEB2 | O60315 | zinc finger E-box binding homeobox 2 | DisGeNet/OMIM |
| 610 | ZFP36 | P26651 | ZFP36 ring finger protein | DisGeNet |
| 611 | ZGLP1 | P0C6A0 | zinc finger, GATA-like protein 1 | DisGeNet |
| 612 | ZNF410 | Q86VK4 | zinc finger protein 410 | DisGeNet |
| 613 | AGRP | O00253 | Agouti-related protein | OMIM |
| 614 | AHSG | P02765 | Alpha-2-HS-glycoprotein | OMIM |
| 615 | AIFM1 | O95831 | Apoptosis-inducing factor 1, mitochondrial | OMIM |
| 616 | AIRE | O43918 | Autoimmune regulator | OMIM |
| 617 | AKT2 | P31751 | RAC-beta serine/threonine-protein kinase | OMIM |
| 618 | ALMS1 | Q8TCU4 | Alstrom syndrome protein 1 | OMIM |
| 619 | ALOX5 | P09917 | Arachidonate 5-lipoxygenase | OMIM |
| 620 | ANGPTL3 | Q9Y5C1 | Angiopoietin-related protein 3 | OMIM |
| 621 | ANGPTL4 | Q9BY76 | Angiopoietin-related protein 4 | OMIM |
| 622 | APCS | P02743 | Serum amyloid P-component | OMIM |
| 623 | APOC2 | P02655 | Apolipoprotein C-II | OMIM |
| 624 | APOO | Q9BUR5 | MICOS complex subunit MIC26 | OMIM |
| 625 | APP | P05067 | Amyloid-beta precursor protein | OMIM |
| 626 | APPL1 | Q9UKG1 | DCC-interacting protein 13-alpha | OMIM |
| 627 | AQP3 | Q92482 | Aquaporin-3 | OMIM |
| 628 | AQP4 | P55087 | Aquaporin-4 | OMIM |
| 629 | AQP7 | O14520 | Aquaporin-7 | OMIM |
| 630 | AQP9 | O43315 | Aquaporin-9 | OMIM |
| 631 | ARHGAP4 | P98171 | Rho GTPase-activating protein 4 | OMIM |
| 632 | ARNTL | O00327 | Aryl hydrocarbon receptor nuclear translocator-like protein 1 | OMIM |
| 633 | ART1 | P52961 | GPI-linked NA | OMIM |
| 634 | ARV1 | Q9H2C2 | Protein ARV1 | OMIM |
| 635 | ASIP | P42127 | Agouti-signaling protein | OMIM |
| 636 | ATP2A3 | Q93084 | Sarcoplasmic/endoplasmic reticulum calcium ATPase 3 | OMIM |
| 637 | AVP | P01185 | Vasopressin-neurophysin 2-copeptin | OMIM |
| 638 | AVPR1A | P37288 | Vasopressin V1a receptor | OMIM |
| 639 | AVPR1B | P47901 | Vasopressin V1b receptor | OMIM |
| 640 | AVPR2 | P30518 | Vasopressin V2 receptor | OMIM |
| 641 | B2M | P61769 | Beta-2-microglobulin | OMIM |
| 642 | BBS10 | Q8TAM1 | Bardet-Biedl syndrome 10 protein | OMIM |
| 643 | BBS4 | Q96RK4 | Bardet-Biedl syndrome 4 protein | OMIM |
| 644 | BGLAP | P02818 | Osteocalcin | OMIM |
| 645 | BICC1 | Q9H694 | Protein bicaudal C homolog 1 | OMIM |
| 646 | BLK | P51451 | Tyrosine-protein kinase Blk | OMIM |
| 647 | BSCL2 | Q96G97 | Seipin | OMIM |
| 648 | BTK | Q06187 | Tyrosine-protein kinase BTK | OMIM |
| 649 | C1QA | P02745 | Complement C1q subcomponent subunit A | OMIM |
| 650 | C1QL3 | Q5VWW1 | Complement C1q-like protein 3 | OMIM |
| 651 | CA1 | P00915 | Carbonic anhydrase 1 | OMIM |
| 652 | CAMK2A | Q9UQM7 | Calcium/calmodulin-dependent protein kinase type II subunit alpha | OMIM |
| 653 | CAPN10 | Q9HC96 | Calpain-10 | OMIM |
| 654 | CARTPT | Q16568 | Cocaine- and amphetamine-regulated transcript protein | OMIM |
| 655 | CASP10 | Q92851 | Caspase-10 | OMIM |
| 656 | CASP3 | P42574 | Caspase-3 | CTD/OMIM |
| 657 | CAV2 | P51636 | Caveolin-2 | OMIM |
| 658 | CAV3 | P56539 | Caveolin-3 | OMIM |
| 659 | CAVIN1 | Q6NZI2 | Caveolae-associated protein 1 | OMIM |
| 660 | CBLB | Q13191 | E3 ubiquitin-protein ligase CBL-B | OMIM |
| 661 | CCK | P06307 | Cholecystokinin | OMIM |
| 662 | CCKAR | P32238 | Cholecystokinin receptor type A | OMIM |
| 663 | CCL13 | Q99616 | C-C motif chemokine 13 | OMIM |
| 664 | CCL21 | O00585 | C-C motif chemokine 21 | OMIM |
| 665 | CCND1 | P24385 | G1/S-specific cyclin-D1 | OMIM |
| 666 | CCR7 | P32248 | C-C chemokine receptor type 7 | OMIM |
| 667 | CD14 | P08571 | Monocyte differentiation antigen CD14 | OMIM |
| 668 | CD151 | P48509 | CD151 antigen | OMIM |
| 669 | CD19 | P15391 | B-lymphocyte antigen CD19 | OMIM |
| 670 | CD1D | P15813 | Antigen-presenting glycoprotein CD1d | OMIM |
| 671 | CD34 | P28906 | Hematopoietic progenitor cell antigen CD34 | OMIM |
| 672 | CD3E | P07766 | T-cell surface glycoprotein CD3 epsilon chain | OMIM |
| 673 | CD40LG | P29965 | CD40 ligand | OMIM |
| 674 | CD8A | P01732 | T-cell surface glycoprotein CD8 alpha chain | OMIM |
| 675 | CDK19 | Q9BWU1 | Cyclin-dependent kinase 19 | OMIM |
| 676 | CDK4 | P11802 | Cyclin-dependent kinase 4 | OMIM |
| 677 | CDK5 | Q00535 | Cyclin-dependent-like kinase 5 | OMIM |
| 678 | CDKN1B | P46527 | Cyclin-dependent kinase inhibitor 1B | OMIM |
| 679 | CDKN2B | P42772 | Cyclin-dependent kinase 4 inhibitor B | OMIM |
| 680 | CEBPB | P17676 | CCAAT/enhancer-binding protein beta | OMIM |
| 681 | CEL | P19835 | Bile salt-activated lipase | OMIM |
| 682 | CELA2A | P08217 | Chymotrypsin-like elastase family member 2A | OMIM |
| 683 | CFLAR | O15519 | CASP8 and FADD-like apoptosis regulator | OMIM |
| 684 | CFTR | P13569 | Cystic fibrosis transmembrane conductance regulator | OMIM |
| 685 | CHGA | P10645 | Chromogranin-A | OMIM |
| 686 | CIDEA | O60543 | Cell death activator CIDE-A | OMIM |
| 687 | CIDEC | Q96AQ7 | Cell death activator CIDE-3 | OMIM |
| 688 | CISD2 | Q8N5K1 | CDGSH iron-sulfur domain-containing protein 2 | OMIM |
| 689 | CLCNKA | P51800 | Chloride channel protein ClC-Ka | OMIM |
| 690 | CLDN10 | P78369 | Claudin-10 | OMIM |
| 691 | CLEC16A | Q2KHT3 | Protein CLEC16A | OMIM |
| 692 | CLMP | Q9H6B4 | CXADR-like membrane protein | OMIM |
| 693 | CLOCK | O15516 | Circadian locomoter output cycles protein kaput | OMIM |
| 694 | CLTCL1 | P53675 | Clathrin heavy chain 2 | OMIM |
| 695 | CNTF | P26441 | Ciliary neurotrophic factor | OMIM |
| 696 | COL4A5 | P29400 | Collagen alpha-5(IV) chain | OMIM |
| 697 | COL5A1 | P20908 | Collagen alpha-1(V) chain | OMIM |
| 698 | COQ2 | Q96H96 | 4-hydroxybenzoate polyprenyltransferase, mitochondrial | OMIM |
| 699 | CP | P00450 | Ceruloplasmin | OMIM |
| 700 | CPE | P16870 | Carboxypeptidase E | OMIM |
| 701 | CPPED1 | Q9BRF8 | Serine/threonine-protein phosphatase CPPED1 | OMIM |
| 702 | CRP | P02741 | C-reactive protein | OMIM |
| 703 | CRTAM | O95727 | Cytotoxic and regulatory T-cell molecule | OMIM |
| 704 | CRTC2 | Q53ET0 | CREB-regulated transcription coactivator 2 | OMIM |
| 705 | CSF3 | P09919 | Granulocyte colony-stimulating factor | OMIM |
| 706 | CTLA4 | P16410 | Cytotoxic T-lymphocyte protein 4 | OMIM |
| 707 | CTSA | P10619 | Lysosomal protective protein | OMIM |
| 708 | CTSL | P07711 | Cathepsin L1 | OMIM |
| 709 | CX3CR1 | P49238 | CX3C chemokine receptor 1 | OMIM |
| 710 | CXCL12 | P48061 | Stromal cell-derived factor 1 | OMIM |
| 711 | CXCR3 | P49682 | C-X-C chemokine receptor type 3 | OMIM |
| 712 | CYB5R4 | Q7L1T6 | Cytochrome b5 reductase 4 | OMIM |
| 713 | CYP19A1 | P11511 | Aromatase | OMIM |
| 714 | CYP4A11 | Q02928 | Cytochrome P450 4A11 | OMIM |
| 715 | DCAF17 | Q5H9S7 | DDB1- and CUL4-associated factor 17 | OMIM |
| 716 | DCXR | Q7Z4W1 | L-xylulose reductase | OMIM |
| 717 | DDX42 | Q86XP3 | ATP-dependent RNA helicase DDX42 | OMIM |
| 718 | DGKD | Q16760 | Diacylglycerol kinase delta | OMIM |
| 719 | DLK1 | P80370 | Protein delta homolog 1 | OMIM |
| 720 | DLL1 | O00548 | Delta-like protein 1 | OMIM |
| 721 | DLL4 | Q9NR61 | Delta-like protein 4 | OMIM |
| 722 | DMXL2 | Q8TDJ6 | DmX-like protein 2 | OMIM |
| 723 | DNAJC3 | Q13217 | DnaJ homolog subfamily C member 3 | OMIM |
| 724 | DYRK1B | Q9Y463 | Dual specificity tyrosine-phosphorylation-regulated kinase 1B | OMIM |
| 725 | E2F2 | Q14209 | Transcription factor E2F2 | OMIM |
| 726 | ECE2 | P0DPD6 | Endothelin-converting enzyme 2 | OMIM |
| 727 | ECI2 | O75521 | Enoyl-CoA delta isomerase 2 | OMIM |
| 728 | EED | O75530 | Polycomb protein EED | OMIM |
| 729 | EIF2AK3 | Q9NZJ5 | Eukaryotic translation initiation factor 2-alpha kinase 3 | OMIM |
| 730 | EIF2S1 | P05198 | Eukaryotic translation initiation factor 2 subunit 1 | OMIM |
| 731 | ERBB3 | P21860 | Receptor tyrosine-protein kinase erbB-3 | OMIM |
| 732 | ERO1B | Q86YB8 | ERO1-like protein beta | OMIM |
| 733 | ETV6 | P41212 | Transcription factor ETV6 | OMIM |
| 734 | FABP2 | P12104 | Fatty acid-binding protein, intestinal | OMIM |
| 735 | FADS1 | O60427 | Acyl-CoA (8-3)-desaturase | OMIM |
| 736 | FAS | P25445 | Tumor necrosis factor receptor superfamily member 6 | OMIM |
| 737 | FASN | P49327 | Fatty acid synthase | OMIM |
| 738 | FAT1 | Q14517 | Protocadherin Fat 1 | OMIM |
| 739 | FBN1 | P35555 | Fibrillin-1 | OMIM |
| 740 | FBP1 | P09467 | Fructose-1,6-bisphosphatase 1 | OMIM |
| 741 | FCAMR | Q8WWV6 | High affinity immunoglobulin alpha and immunoglobulin mu Fc receptor | OMIM |
| 742 | FCN3 | O75636 | Ficolin-3 | OMIM |
| 743 | FFAR4 | Q5NUL3 | Free fatty acid receptor 4 | OMIM |
| 744 | FGF1 | P05230 | Fibroblast growth factor 1 | OMIM |
| 745 | FGF19 | O95750 | Fibroblast growth factor 19 | OMIM |
| 746 | FGF8 | P55075 | Fibroblast growth factor 8 | OMIM |
| 747 | FN3KRP | Q9HA64 | Ketosamine-3-kinase | OMIM |
| 748 | FOXA2 | Q9Y261 | Hepatocyte nuclear factor 3-beta | OMIM |
| 749 | FOXC2 | Q99958 | Forkhead box protein C2 | OMIM |
| 750 | FSTL1 | Q12841 | Follistatin-related protein 1 | OMIM |
| 751 | FTO | Q9C0B1 | Alpha-ketoglutarate-dependent dioxygenase FTO | OMIM |
| 752 | FUT7 | Q11130 | Alpha-(1,3)-fucosyltransferase 7 | OMIM |
| 753 | FXN | Q16595 | Frataxin, mitochondrial | OMIM |
| 754 | G6PC2 | Q9NQR9 | Glucose-6-phosphatase 2 | OMIM |
| 755 | GAD1 | Q99259 | Glutamate decarboxylase 1 | OMIM |
| 756 | GAD2 | Q05329 | Glutamate decarboxylase 2 | OMIM |
| 757 | GAL | P22466 | Galanin peptides | OMIM |
| 758 | GANC | Q8TET4 | Neutral alpha-glucosidase C | OMIM |
| 759 | GATA6 | Q92908 | Transcription factor GATA-6 | OMIM |
| 760 | GC | P02774 | Vitamin D-binding protein | OMIM |
| 761 | GCGR | P47871 | Glucagon receptor | OMIM |
| 762 | GCKR | Q14397 | Glucokinase regulatory protein | OMIM |
| 763 | GDNF | P39905 | Glial cell line-derived neurotrophic factor | OMIM |
| 764 | GEM | P55040 | GTP-binding protein GEM | OMIM |
| 765 | GIMAP5 | Q96F15 | GTPase IMAP family member 5 | OMIM |
| 766 | GIP | P09681 | Gastric inhibitory polypeptide | OMIM |
| 767 | GJA4 | P35212 | Gap junction alpha-4 protein | OMIM |
| 768 | GJD2 | Q9UKL4 | Gap junction delta-2 protein | OMIM |
| 769 | GK | P32189 | Glycerol kinase | OMIM |
| 770 | GLI2 | P10070 | Zinc finger protein GLI2 | OMIM |
| 771 | GLIS3 | Q8NEA6 | Zinc finger protein GLIS3 | OMIM |
| 772 | GLUD1 | P00367 | Glutamate dehydrogenase 1 | OMIM |
| 773 | GNA11 | P29992 | Guanine nucleotide-binding protein subunit alpha-11 | OMIM |
| 774 | GP1BA | P07359 | Platelet glycoprotein Ib alpha chain | OMIM |
| 775 | GP2 | P55259 | Pancreatic secretory granule membrane major glycoprotein GP2 | OMIM |
| 776 | GPCPD1 | Q9NPB8 | Glycerophosphocholine phosphodiesterase GPCPD1 | OMIM |
| 777 | GPD2 | P43304 | Glycerol-3-phosphate dehydrogenase | OMIM |
| 778 | GPR1 | P46091 | G-protein coupled receptor 1 | OMIM |
| 779 | GPR161 | Q8N6U8 | G-protein coupled receptor 161 | OMIM |
| 780 | GPR183 | P32249 | G-protein coupled receptor 183 | OMIM |
| 781 | GPR35 | Q9HC97 | G-protein coupled receptor 35 | OMIM |
| 782 | GSK3A | P49840 | Glycogen synthase kinase-3 alpha | OMIM |
| 783 | GSN | P06396 | Gelsolin | OMIM |
| 784 | GYS1 | P13807 | Glycogen [starch] synthase | OMIM |
| 785 | GZMA | P12544 | Granzyme A | OMIM |
| 786 | HADH | Q16836 | Hydroxyacyl-coenzyme A dehydrogenase | OMIM |
| 787 | HAMP | P81172 | Hepcidin (Liver-expressed antimicrobial peptide 1) | OMIM |
| 788 | HAVCR2 | Q8TDQ0 | Hepatitis A virus cellular receptor 2 | OMIM |
| 789 | HBA2 | P69905 | Hemoglobin subunit alpha | OMIM |
| 790 | HJV | Q6ZVN8 | Hemojuvelin | OMIM |
| 791 | HK2 | P52789 | Hexokinase-2 | OMIM |
| 792 | HKDC1 | Q2TB90 | Hexokinase HKDC1 | OMIM |
| 793 | HLA-A | P30450 | HLA class I histocompatibility antigen, A-26 alpha chain | OMIM |
| 794 | HLA-DQB1 | P01920 | HLA class II histocompatibility antigen, DQ beta 1 chain | OMIM |
| 795 | HLA-DRA | P01903 | HLA class II histocompatibility antigen, DR alpha chain | OMIM |
| 796 | HMGA1 | P17096 | High mobility group protein HMG-I/HMG-Y | OMIM |
| 797 | HMOX1 | P09601 | Heme oxygenase 1 | CTD/OMIM |
| 798 | HMSD | A8MTL9 | Serpin-like protein HMSD | OMIM |
| 799 | HOXB4 | P17483 | Homeobox protein Hox-B4 | OMIM |
| 800 | HSD11B1 | P28845 | Corticosteroid 11-beta-dehydrogenase isozyme 1 | OMIM |
| 801 | HTR1A | P08908 | 5-hydroxytryptamine receptor 1A | OMIM |
| 802 | ICA1 | Q05084 | Islet cell autoantigen 1 | OMIM |
| 803 | IER3IP1 | Q9Y5U9 | Immediate early response 3-interacting protein 1 | OMIM |
| 804 | IFIH1 | Q9BYX4 | Interferon-induced helicase C domain-containing protein 1 | OMIM |
| 805 | IGF1R | P08069 | Insulin-like growth factor 1 receptor | OMIM |
| 806 | IKBKB | O14920 | Inhibitor of nuclear factor kappa-B kinase subunit beta | OMIM |
| 807 | IL12B | P29460 | Interleukin-12 subunit beta | OMIM |
| 808 | IL13 | P35225 | Interleukin-13 | OMIM |
| 809 | IL2 | P60568 | Interleukin-2 | OMIM |
| 810 | IL22 | Q9GZX6 | Interleukin-22 | OMIM |
| 811 | IL2RA | P01589 | Interleukin-2 receptor subunit alpha | OMIM |
| 812 | IL33 | O95760 | Interleukin-33 | OMIM |
| 813 | ILDR2 | Q71H61 | Immunoglobulin-like domain-containing receptor 2 | OMIM |
| 814 | INSIG1 | O15503 | Insulin-induced gene 1 protein | OMIM |
| 815 | INSIG2 | Q9Y5U4 | Insulin-induced gene 2 protein | OMIM |
| 816 | IP6K1 | Q92551 | Inositol hexakisphosphate kinase 1 | OMIM |
| 817 | IRF7 | Q92985 | Interferon regulatory factor 7 | OMIM |
| 818 | IRGM | A1A4Y4 | Immunity-related GTPase family M protein | OMIM |
| 819 | ISL1 | P61371 | Insulin gene enhancer protein ISL-1 | OMIM |
| 820 | ITGB7 | P26010 | Integrin beta-7 | OMIM |
| 821 | ITPR2 | Q14571 | Inositol 1,4,5-trisphosphate receptor type 2 | OMIM |
| 822 | ITPR3 | Q14573 | Inositol 1,4,5-trisphosphate receptor type 3 | OMIM |
| 823 | KAT2A | Q92830 | Histone acetyltransferase KAT2A | OMIM |
| 824 | KCNE2 | Q9Y6J6 | Potassium voltage-gated channel subfamily E member 2 | OMIM |
| 825 | KCNH2 | Q12809 | Potassium voltage-gated channel subfamily H member 2 | OMIM |
| 826 | KCNJ15 | Q99712 | ATP-sensitive inward rectifier potassium channel 15 | OMIM |
| 827 | KCNJ6 | P48051 | G protein-activated inward rectifier potassium channel 2 | OMIM |
| 828 | KHK | P50053 | Ketohexokinase | OMIM |
| 829 | KIRREL2 | Q6UWL6 | Kin of IRRE-like protein 2 | OMIM |
| 830 | KLF11 | O14901 | Krueppel-like factor 11 | OMIM |
| 831 | LARS2 | Q15031 | Probable leucine--tRNA ligase | OMIM |
| 832 | LCK | P06239 | Tyrosine-protein kinase Lck | OMIM |
| 833 | LDHB | P07195 | L-lactate dehydrogenase B chain | OMIM |
| 834 | LDLR | P01130 | Low-density lipoprotein receptor | OMIM |
| 835 | LEPR | P48357 | Leptin receptor | OMIM |
| 836 | LEPROT | O15243 | Leptin receptor gene-related protein | OMIM |
| 837 | LEPROTL1 | O95214 | Leptin receptor overlapping transcript-like 1 | OMIM |
| 838 | LGALSL | Q3ZCW2 | Galectin-related protein | OMIM |
| 839 | LIG4 | P49917 | DNA ligase 4 | OMIM |
| 840 | LIPE | Q05469 | Hormone-sensitive lipase | OMIM |
| 841 | LMNB2 | Q03252 | Lamin-B2 | OMIM |
| 842 | LMX1B | O60663 | LIM homeobox transcription factor 1-beta | OMIM |
| 843 | LPIN1 | Q14693 | Phosphatidate phosphatase LPIN1 | OMIM |
| 844 | LTBR | P36941 | Tumor necrosis factor receptor superfamily member 3 | OMIM |
| 845 | MAFA | Q8NHW3 | Transcription factor MafA | OMIM |
| 846 | MAP2K1 | Q02750 | Dual specificity mitogen-activated protein kinase kinase 1 | OMIM |
| 847 | MAPK8IP1 | Q9UQF2 | C-Jun-amino-terminal kinase-interacting protein 1 | OMIM |
| 848 | MAPK8IP2 | Q13387 | C-Jun-amino-terminal kinase-interacting protein 2 | OMIM |
| 849 | MAPK9 | P45984 | Mitogen-activated protein kinase 9 | OMIM |
| 850 | MC2R | Q01718 | Adrenocorticotropic hormone receptor | OMIM |
| 851 | MC3R | P41968 | Melanocortin receptor 3 | OMIM |
| 852 | MC4R | P32245 | Melanocortin receptor 4 | OMIM |
| 853 | MCU | Q8NE86 | Calcium uniporter protein | OMIM |
| 854 | MGAT4A | Q9UM21 | Alpha-1,3-mannosyl-glycoprotein 4-beta-N-acetylglucosaminyltransferase A | OMIM |
| 855 | MGST3 | O14880 | Microsomal glutathione S-transferase 3 | OMIM |
| 856 | MICA | Q29983 | MHC class I polypeptide-related sequence A | OMIM |
| 857 | MIOX | Q9UGB7 | Inositol oxygenase | OMIM |
| 858 | MKKS | Q9NPJ1 | McKusick-Kaufman/Bardet-Biedl syndromes putative chaperonin | OMIM |
| 859 | MMP14 | P50281 | Matrix metalloproteinase-14 | OMIM |
| 860 | MOG | Q16653 | Myelin-oligodendrocyte glycoprotein | OMIM |
| 861 | MPV17 | P39210 | Protein Mpv17 | OMIM |
| 862 | MRPS31 | Q92665 | 28S ribosomal protein S31 | OMIM |
| 863 | MSTN | O14793 | Growth/differentiation factor 8 | OMIM |
| 864 | MT-ATP8 | P03928 | ATP synthase protein 8 | OMIM |
| 865 | MT-ND3 | P03897 | NADH-ubiquinone oxidoreductase chain 3 | OMIM |
| 866 | MTNR1B | P49286 | Melatonin receptor type 1B | OMIM |
| 867 | MTTP | P55157 | Microsomal triglyceride transfer protein large subunit | OMIM |
| 868 | MUC20 | Q8N307 | Mucin-20 | OMIM |
| 869 | MYD88 | Q99836 | Myeloid differentiation primary response protein MyD88 | OMIM |
| 870 | NAT8 | Q9UHE5 | N-acetyltransferase 8 | OMIM |
| 871 | NDUFB6 | O95139 | NADH dehydrogenase [ubiquinone] 1 beta subcomplex subunit 6 | OMIM |
| 872 | NEFL | P07196 | Neurofilament light polypeptide | OMIM |
| 873 | NEUROD4 | Q9HD90 | Neurogenic differentiation factor 4 | OMIM |
| 874 | NEUROG3 | Q9Y4Z2 | Neurogenin-3 | OMIM |
| 875 | NFATC1 | O95644 | Nuclear factor of activated T-cells, cytoplasmic 1 | OMIM |
| 876 | NIT2 | Q9NQR4 | Omega-amidase NIT2 | OMIM |
| 877 | NKX2-2 | O95096 | Homeobox protein Nkx-2.2 | OMIM |
| 878 | NNMT | P40261 | Nicotinamide N-methyltransferase | OMIM |
| 879 | NOD1 | Q9Y239 | Nucleotide-binding oligomerization domain-containing protein 1 | OMIM |
| 880 | NR3C1 | P04150 | Glucocorticoid receptor | OMIM |
| 881 | NR4A1 | P22736 | Nuclear receptor subfamily 4 group A member 1 | OMIM |
| 882 | NR4A2 | P43354 | Nuclear receptor subfamily 4 group A member 2 | OMIM |
| 883 | NR4A3 | Q92570 | Nuclear receptor subfamily 4 group A member 3 | OMIM |
| 884 | NTN1 | O95631 | Netrin-1 | OMIM |
| 885 | OAS1 | P00973 | 2'-5'-oligoadenylate synthase 1 | OMIM |
| 886 | OGA | O60502 | Protein O-GlcNAcase | OMIM |
| 887 | OGT | O15294 | UDP-N-acetylglucosamine--peptide N-acetylglucosaminyltransferase 110 kDa subunit | OMIM |
| 888 | OIT3 | Q8WWZ8 | Oncoprotein-induced transcript 3 protein | OMIM |
| 889 | ONECUT1 | Q9UBC0 | Hepatocyte nuclear factor 6 | OMIM |
| 890 | OXT | P01178 | Oxytocin-neurophysin 1 | OMIM |
| 891 | P2RX7 | Q99572 | P2X purinoceptor 7 | OMIM |
| 892 | P2RY12 | Q9H244 | P2Y purinoceptor 12 | OMIM |
| 893 | PARP1 | P09874 | Poly [ADP-ribose] polymerase 1 | OMIM |
| 894 | PAX6 | P26367 | Paired box protein Pax-6 | OMIM |
| 895 | PBX1 | P40424 | Pre-B-cell leukemia transcription factor 1 | OMIM |
| 896 | PCBD1 | P61457 | Pterin-4-alpha-carbinolamine dehydratase | OMIM |
| 897 | PCK1 | P35558 | Phosphoenolpyruvate carboxykinase, cytosolic [GTP] | OMIM |
| 898 | PCNT | O95613 | Pericentrin | OMIM |
| 899 | PDE3B | Q13370 | cGMP-inhibited 3',5'-cyclic phosphodiesterase B | OMIM |
| 900 | PDE4D | Q08499 | cAMP-specific 3',5'-cyclic phosphodiesterase 4D | OMIM |
| 901 | PDGFB | P01127 | Platelet-derived growth factor subunit B | OMIM |
| 902 | PDGFC | Q9NRA1 | Platelet-derived growth factor C | OMIM |
| 903 | PDPK1 | O15530 | 3-phosphoinositide-dependent protein kinase 1 | OMIM |
| 904 | PDSS2 | Q86YH6 | Decaprenyl-diphosphate synthase subunit 2 | OMIM |
| 905 | PGR | P06401 | Progesterone receptor | OMIM |
| 906 | PHKG2 | P15735 | Phosphorylase b kinase gamma catalytic chain, liver/testis isoform | OMIM |
| 907 | PKHD1 | P08F94 | Fibrocystin | OMIM |
| 908 | PLA2G7 | Q13093 | Platelet-activating factor acetylhydrolase | OMIM |
| 909 | PLAGL1 | Q9UM63 | Zinc finger protein PLAGL1 | OMIM |
| 910 | PLIN1 | O60240 | Perilipin-1 | OMIM |
| 911 | POC5 | Q8NA72 | Centrosomal protein POC5 | OMIM |
| 912 | PODN | Q7Z5L7 | Podocan | OMIM |
| 913 | POLD1 | P28340 | DNA polymerase delta catalytic subunit | OMIM |
| 914 | POMC | P01189 | Pro-opiomelanocortin | OMIM |
| 915 | PPARGC1B | Q86YN6 | Peroxisome proliferator-activated receptor gamma coactivator 1-beta | OMIM |
| 916 | PPP1R15B | Q5SWA1 | Protein phosphatase 1 regulatory subunit 15B | OMIM |
| 917 | PPP1R2 | P41236 | Protein phosphatase inhibitor 2 | OMIM |
| 918 | PPP1R3A | Q16821 | Protein phosphatase 1 regulatory subunit 3A | OMIM |
| 919 | PPP1R3B | Q86XI6 | Protein phosphatase 1 regulatory subunit 3B | OMIM |
| 920 | PPP3CA | Q08209 | Serine/threonine-protein phosphatase 2B catalytic subunit alpha isoform | OMIM |
| 921 | PRDM16 | Q9HAZ2 | Histone-lysine N-methyltransferase PRDM16 | OMIM |
| 922 | PRKCD | Q05655 | Protein kinase C delta type | OMIM |
| 923 | PRKD1 | Q15139 | Serine/threonine-protein kinase D1 | OMIM |
| 924 | PRLHR | P49683 | Prolactin-releasing peptide receptor | OMIM |
| 925 | PROK2 | Q9HC23 | Prokineticin-2 | OMIM |
| 926 | PRPH | P41219 | Peripherin | OMIM |
| 927 | PRSS16 | Q9NQE7 | Thymus-specific serine protease | OMIM |
| 928 | PSMB8 | P28062 | Proteasome subunit beta type-8 | OMIM |
| 929 | PSMB9 | P28065 | Proteasome subunit beta type-9 | OMIM |
| 930 | PTF1A | Q7RTS3 | Pancreas transcription factor 1 subunit alpha | OMIM |
| 931 | PTGES2 | Q9H7Z7 | Prostaglandin E synthase 2 | OMIM |
| 932 | PTPN2 | P17706 | Tyrosine-protein phosphatase non-receptor type 2 | OMIM |
| 933 | PTPN22 | Q9Y2R2 | Tyrosine-protein phosphatase non-receptor type 22 | OMIM |
| 934 | PTPRC | P08575 | Receptor-type tyrosine-protein phosphatase C | OMIM |
| 935 | PTPRN | Q16849 | Receptor-type tyrosine-protein phosphatase-like N | OMIM |
| 936 | PTPRN2 | Q92932 | Receptor-type tyrosine-protein phosphatase N2 | OMIM |
| 937 | PTTG1 | O95997 | Securin | OMIM |
| 938 | RBM17 | Q96I25 | Splicing factor 45 | OMIM |
| 939 | RC3H1 | Q5TC82 | Roquin-1 | OMIM |
| 940 | REG1A | P05451 | Lithostathine-1-alpha | OMIM |
| 941 | RESP18 | Q5W5W9 | Regulated endocrine-specific protein 18 | OMIM |
| 942 | RETN | Q9HD89 | Resistin | OMIM |
| 943 | RFX6 | Q8HWS3 | DNA-binding protein RFX6 | OMIM |
| 944 | RHOA | P61586 | Transforming protein RhoA | OMIM |
| 945 | RNPEPL1 | Q9HAU8 | Aminopeptidase RNPEPL1 | OMIM |
| 946 | RPS6KA3 | P51812 | Ribosomal protein S6 kinase alpha-3 | OMIM |
| 947 | RPS6KB1 | P23443 | Ribosomal protein S6 kinase beta-1 | OMIM |
| 948 | RRM2B | Q7LG56 | Ribonucleoside-diphosphate reductase subunit M2 B | OMIM |
| 949 | SCARB1 | Q8WTV0 | Scavenger receptor class B member 1 | OMIM |
| 950 | SCARB2 | Q14108 | Lysosome membrane protein 2 | OMIM |
| 951 | SCGB1A1 | P11684 | Uteroglobin | OMIM |
| 952 | SEL1L | Q9UBV2 | Protein sel-1 homolog 1 | OMIM |
| 953 | SELENOS | Q9BQE4 | Selenoprotein S | OMIM |
| 954 | SELPLG | Q14242 | P-selectin glycoprotein ligand 1 | OMIM |
| 955 | SERPINA12 | Q8IW75 | Serpin A12 | OMIM |
| 956 | SFRP1 | Q8N474 | Secreted frizzled-related protein 1 | OMIM |
| 957 | SFRP5 | Q5T4F7 | Secreted frizzled-related protein 5 | OMIM |
| 958 | SGIP1 | Q9BQI5 | SH3-containing GRB2-like protein 3-interacting protein 1 | OMIM |
| 959 | SH2B3 | Q9UQQ2 | SH2B adapter protein 3 | OMIM |
| 960 | SHC1 | P29353 | SHC-transforming protein 1 | OMIM |
| 961 | SHH | Q15465 | Sonic hedgehog protein | OMIM |
| 962 | SIAE | Q9HAT2 | Sialate O-acetylesterase | OMIM |
| 963 | SIGLEC5 | O15389 | Sialic acid-binding Ig-like lectin 5 | OMIM |
| 964 | SIK2 | Q9H0K1 | Serine/threonine-protein kinase SIK2 | OMIM |
| 965 | SIRPA | P78324 | Tyrosine-protein phosphatase non-receptor type substrate 1 | OMIM |
| 966 | SIX3 | O95343 | Homeobox protein SIX3 | OMIM |
| 967 | SLC11A1 | P49279 | Natural resistance-associated macrophage protein 1 | OMIM |
| 968 | SLC12A1 | Q13621 | Solute carrier family 12 member 1 | OMIM |
| 969 | SLC14A1 | Q13336 | Urea transporter 1 | OMIM |
| 970 | SLC16A1 | P53985 | Monocarboxylate transporter 1 | OMIM |
| 971 | SLC16A11 | Q8NCK7 | Monocarboxylate transporter 11 | OMIM |
| 972 | SLC16A9 | Q7RTY1 | Monocarboxylate transporter 9 | OMIM |
| 973 | SLC19A2 | O60779 | Thiamine transporter 1 | OMIM |
| 974 | SLC22A1 | O15245 | Solute carrier family 22 member 1 | OMIM |
| 975 | SLC29A3 | Q9BZD2 | Equilibrative nucleoside transporter 3 | OMIM |
| 976 | SLC2A10 | O95528 | Solute carrier family 2, facilitated glucose transporter member 10 | OMIM |
| 977 | SLC2A4 | P14672 | Solute carrier family 2, facilitated glucose transporter member 4 | OMIM |
| 978 | SLC30A8 | Q8IWU4 | Zinc transporter 8 | OMIM |
| 979 | SLC37A4 | O43826 | Glucose-6-phosphate exchanger SLC37A4 | OMIM |
| 980 | SLC38A2 | Q96QD8 | Sodium-coupled neutral amino acid transporter 2 | OMIM |
| 981 | SLC3A2 | P08195 | 4F2 cell-surface antigen heavy chain | OMIM |
| 982 | SLC5A1 | P13866 | Sodium/glucose cotransporter 1 | OMIM |
| 983 | SMARCB1 | Q12824 | SWI/SNF-related matrix-associated actin-dependent regulator of chromatin subfamily B member 1 | OMIM |
| 984 | SMPDL3B | Q92485 | Acid sphingomyelinase-like phosphodiesterase 3b | OMIM |
| 985 | SOCS2 | O14508 | Suppressor of cytokine signaling 2 | OMIM |
| 986 | SORCS1 | Q8WY21 | VPS10 domain-containing receptor SorCS1 | OMIM |
| 987 | SOX13 | Q9UN79 | Transcription factor SOX-13 | OMIM |
| 988 | SPINT3 | P49223 | Kunitz-type protease inhibitor 3 | OMIM |
| 989 | SREBF1 | P36956 | Sterol regulatory element-binding protein 1 | OMIM |
| 990 | ST3GAL5 | Q9UNP4 | Lactosylceramide alpha-2,3-sialyltransferase | OMIM |
| 991 | STAB1 | Q9NY15 | Stabilin-1 | OMIM |
| 992 | STAB2 | Q8WWQ8 | Stabilin-2 | OMIM |
| 993 | STARD10 | Q9Y365 | START domain-containing protein 10 | OMIM |
| 994 | STAT6 | P42226 | Signal transducer and activator of transcription 6 | OMIM |
| 995 | STK11 | Q15831 | Serine/threonine-protein kinase STK11 | OMIM |
| 996 | STK17B | O94768 | Serine/threonine-protein kinase 17B | OMIM |
| 997 | SULT4A1 | Q9BR01 | Sulfotransferase 4A1 | OMIM |
| 998 | TAC1 | P20366 | Protachykinin-1 | OMIM |
| 999 | TAGAP | Q8N103 | T-cell activation Rho GTPase-activating protein | OMIM |
| 1000 | TAP1 | Q03518 | Antigen peptide transporter 1 | OMIM |
| 1001 | TBC1D4 | O60343 | TBC1 domain family member 4 | OMIM |
| 1002 | TET2 | Q6N021 | Methylcytosine dioxygenase TET2 | OMIM |
| 1003 | TFAM | Q00059 | Transcription factor A, mitochondrial | OMIM |
| 1004 | TFAP2B | Q92481 | Transcription factor AP-2-beta | OMIM |
| 1005 | TFE3 | P19532 | Transcription factor E3 | OMIM |
| 1006 | TGIF1 | Q15583 | Homeobox protein TGIF1 | OMIM |
| 1007 | TH | P07101 | Tyrosine 3-monooxygenase | OMIM |
| 1008 | THADA | Q6YHU6 | Thyroid adenoma-associated protein | OMIM |
| 1009 | THRSP | Q92748 | Thyroid hormone-inducible hepatic protein | OMIM |
| 1010 | THSD7A | Q9UPZ6 | Thrombospondin type-1 domain-containing protein 7A | OMIM |
| 1011 | TMEM131L | A2VDJ0 | Transmembrane protein 131-like | OMIM |
| 1012 | TNFRSF8 | P28908 | Tumor necrosis factor receptor superfamily member 8 | OMIM |
| 1013 | TNFSF14 | O43557 | Tumor necrosis factor ligand superfamily member 14 | OMIM |
| 1014 | TNNI2 | P48788 | Troponin I, fast skeletal muscle | OMIM |
| 1015 | TOR1A | O14656 | Torsin-1A | OMIM |
| 1016 | TP53 | P04637 | Cellular tumor antigen p53 | OMIM |
| 1017 | TP63 | Q9H3D4 | Tumor protein 63 | OMIM |
| 1018 | TP73 | O15350 | Tumor protein p73 | OMIM |
| 1019 | TRAPPC2 | P0DI81 | Trafficking protein particle complex subunit 2 | OMIM |
| 1020 | TREX1 | Q9NSU2 | Three-prime repair exonuclease 1 | OMIM |
| 1021 | TRIB3 | Q96RU7 | Tribbles homolog 3 | OMIM |
| 1022 | TRMT10A | Q8TBZ6 | tRNA methyltransferase 10 homolog A | OMIM |
| 1023 | TRPV1 | Q8NER1 | Transient receptor potential cation channel subfamily V member 1 | OMIM |
| 1024 | TSPAN8 | P19075 | Tetraspanin-8 | OMIM |
| 1025 | TTR | P02766 | Transthyretin | OMIM |
| 1026 | TWNK | Q96RR1 | Twinkle protein, mitochondrial | OMIM |
| 1027 | UBB | P0CG47 | Polyubiquitin-B | OMIM |
| 1028 | UCN | P55089 | Urocortin | OMIM |
| 1029 | USF1 | P22415 | Upstream stimulatory factor 1 | OMIM |
| 1030 | VEGFB | P49765 | Vascular endothelial growth factor B | OMIM |
| 1031 | VIP | P01282 | VIP peptides [Cleaved into: Intestinal peptide PHV-42 | OMIM |
| 1032 | VPS13B | Q7Z7G8 | Vacuolar protein sorting-associated protein 13B | OMIM |
| 1033 | WDR62 | O43379 | WD repeat-containing protein 62 | OMIM |
| 1034 | WNT5B | Q9H1J7 | Protein Wnt-5b | OMIM |
| 1035 | WRN | Q14191 | Werner syndrome ATP-dependent helicase | OMIM |
| 1036 | XPNPEP3 | Q9NQH7 | Xaa-Pro aminopeptidase 3 | OMIM |
| 1037 | XRCC4 | Q13426 | DNA repair protein XRCC4 | OMIM |
| 1038 | YBX1 | P67809 | Nuclease-sensitive element-binding protein 1 | OMIM |
| 1039 | ZFP57 | Q9NU63 | Zinc finger protein 57 homolog | OMIM |
| 1040 | ZFP69 | Q49AA0 | Zinc finger protein 69 homolog | OMIM |
| 1041 | ZNF543 | Q08ER8 | Zinc finger protein 543 | OMIM |
