# Supplementary material for: Protective effect of Cordyceps sinensis against diabetic kidney disease through promoting proliferation and inhibiting apoptosis of renal proximal tubular cells
Source: BMC Complement Med Ther. 2023 Apr 6;23:109. doi: 10.1186/s12906-023-03901-4 (PMC10077712; doi:10.1186/s12906-023-03901-4)
Supplement: Supplementary file 3 — Additional file 3: Table S3. Information for overlapped targets after PPI analysis. [file 12906_2023_3901_MOESM3_ESM.docx]

**Table S3** Information for overlapped targets after PPI analysis.

| **Number** | **Gene Name** | **Protein name** | **Degree** | **Betweenness Centrality** | **Closeness Centrality** |
| --- | --- | --- | --- | --- | --- |
| 1 | MAPK9 | Mitogen-activated protein kinase 9 | 26 | 0.00108313 | 0.51655629 |
| 2 | JUN | Transcription factor Jun | 64 | 0.02826364 | 0.61904762 |
| 3 | AKT1 | RAC-alpha serine/threonine-protein kinase | 95 | 0.08509661 | 0.71232877 |
| 4 | GSK3B | Glycogen synthase kinase-3 beta | 31 | 0.00446852 | 0.53424658 |
| 5 | CDK4 | Cyclin-dependent kinase 4 | 27 | 0.00220246 | 0.51655629 |
| 6 | CCND1 | G1/S-specific cyclin-D1 | 56 | 0.01899349 | 0.6 |
| 7 | MAPK8 | Mitogen-activated protein kinase 8 | 66 | 0.02400469 | 0.624 |
| 8 | VEGFA | Vascular endothelial growth factor A | 88 | 0.06604321 | 0.69026549 |
| 9 | KDR | Vascular endothelial growth factor receptor 2 | 46 | 0.00966449 | 0.57352941 |
| 10 | IKBKB | Inhibitor of nuclear factor kappa-B kinase subunit beta | 23 | 0.00187797 | 0.4875 |
| 11 | TNF | Tumor necrosis factor | 76 | 0.05258046 | 0.65271967 |
| 12 | NOS3 | Nitric oxide synthase | 51 | 0.0150557 | 0.57564576 |
| 13 | PTPN1 | Tyrosine-protein phosphatase non-receptor type 1 | 22 | 0.00028 | 0.50160772 |
| 14 | IGF1R | Insulin-like growth factor 1 receptor | 41 | 0.0128187 | 0.5631769 |
| 15 | PIK3CA | Phosphatidylinositol 4,5-bisphosphate 3-kinase catalytic subunit alpha isoform | 40 | 0.01122657 | 0.54355401 |
| 16 | RELA | Transcription factor p65 | 53 | 0.01848643 | 0.58867925 |
| 17 | TGFBR2 | TGF-beta receptor type-2 | 11 | 0.000347 | 0.45882353 |
| 18 | TGFB1 | Transforming growth factor beta-1 proprotein | 40 | 0.00393768 | 0.54929577 |
| 19 | HIF1A | Hypoxia-inducible factor 1-alpha | 44 | 0.00621774 | 0.55913978 |
| 20 | PTEN | Phosphatidylinositol 3,4,5-trisphosphate 3-phosphatase and dual-specificity protein phosphatase PTEN | 56 | 0.01771373 | 0.6023166 |
| 21 | TGFBR1 | TGF-beta receptor type-1 | 16 | 0.000384 | 0.46706587 |
| 22 | MAPK1 | Mitogen-activated protein kinase 1 | 67 | 0.03073384 | 0.61660079 |
| 23 | MAPK3 | Mitogen-activated protein kinase 3 | 74 | 0.03371878 | 0.6446281 |
| 24 | MAPK14 | Mitogen-activated protein kinase 14 | 52 | 0.00826915 | 0.57992565 |
| 25 | CASP9 | Caspase-9 | 34 | 0.00247618 | 0.53242321 |
| 26 | JAK2 | Tyrosine-protein kinase JAK2 | 42 | 0.00610923 | 0.54736842 |
| 27 | EGF | Pro-epidermal growth factor | 69 | 0.02405419 | 0.62903226 |
| 28 | ESR1 | Estrogen receptor | 50 | 0.01249599 | 0.58646617 |
| 29 | PPARG | Peroxisome proliferator-activated receptor gamma | 59 | 0.04287652 | 0.60465116 |
| 30 | FABP4 | Fatty acid-binding protein | 19 | 0.0035101 | 0.49056604 |
| 31 | MMP9 | Matrix metalloproteinase-9 | 65 | 0.03160033 | 0.609375 |
| 32 | IL1B | Interleukin-1 beta | 64 | 0.02494851 | 0.60700389 |
| 33 | CASP3 | Caspase-3 | 69 | 0.02129165 | 0.63414634 |
| 34 | NLRP3 | NACHT | 24 | 0.00200101 | 0.51147541 |
| 35 | ACACB | Acetyl-CoA carboxylase 2 | 6 | 0.0000575 | 0.40519481 |
| 36 | FASN | Fatty acid synthase S-acetyltransferase ; S-malonyltransferase ; 3-oxoacyl- synthase ; 3-oxoacyl- reductase ; 3-hydroxyacyl- dehydratase ; Enoyl- reductase ; Acyl- hydrolase ] | 21 | 0.00393167 | 0.49681529 |
| 37 | PPARA | Peroxisome proliferator-activated receptor alpha | 30 | 0.01182696 | 0.53608247 |
| 38 | FABP1 | Fatty acid-binding protein | 20 | 0.00534621 | 0.4875 |
| 39 | PRKCA | Protein kinase C alpha type | 21 | 0.00233013 | 0.50980392 |
| 40 | PRKCE | Protein kinase C epsilon type | 16 | 0.00223149 | 0.4469914 |
| 41 | PIK3CB | Phosphatidylinositol 4,5-bisphosphate 3-kinase catalytic subunit beta isoform | 20 | 0.00132665 | 0.47272727 |
| 42 | NR1H4 | Bile acid receptor | 23 | 0.01469497 | 0.51485149 |
| 43 | NR0B2 | Nuclear receptor subfamily 0 group B member 2 | 22 | 0.00778015 | 0.52 |
| 44 | NR3C1 | Glucocorticoid receptor | 39 | 0.0197348 | 0.55319149 |
| 45 | NOS2 | Nitric oxide synthase | 28 | 0.01428119 | 0.52348993 |
| 46 | APP | Amyloid-beta precursor protein | 50 | 0.03537557 | 0.57142857 |
| 47 | ROCK1 | Rho-associated protein kinase 1 | 12 | 0.0000795 | 0.46428571 |
| 48 | PTPN6 | Tyrosine-protein phosphatase non-receptor type 6 | 18 | 0.00210557 | 0.48 |
| 49 | LIPE | Hormone-sensitive lipase | 16 | 0.00408355 | 0.49056604 |
| 50 | PIK3CG | Phosphatidylinositol 4,5-bisphosphate 3-kinase catalytic subunit gamma isoform | 13 | 0.000241 | 0.46017699 |
| 51 | ADAM17 | Disintegrin and metalloproteinase domain-containing protein 17 | 25 | 0.000758 | 0.50649351 |
| 52 | MMP2 | 72 kDa type IV collagenase | 48 | 0.00551691 | 0.56115108 |
| 53 | HMOX1 | Heme oxygenase 1 | 35 | 0.00213401 | 0.53979239 |
| 54 | CYP2C19 | Cytochrome P450 2C19 | 19 | 0.00757821 | 0.45217391 |
| 55 | CYP2C9 | Cytochrome P450 2C9 | 20 | 0.00822164 | 0.4875 |
| 56 | E2F1 | Transcription factor E2F1 | 16 | 0.01407573 | 0.47416413 |
| 57 | GYS1 | Glycogen synthase | 7 | 0.000979 | 0.44956772 |
| 58 | PRKCD | Protein kinase C delta type | 25 | 0.00358672 | 0.50160772 |
| 59 | PRKCB | Protein kinase C beta type | 17 | 0.000756 | 0.47560976 |
| 60 | ALOX5 | Polyunsaturated fatty acid 5-lipoxygenase | 22 | 0.00273485 | 0.51147541 |
| 61 | PTGS2 | Prostaglandin G/H synthase 2 | 65 | 0.02736101 | 0.624 |
| 62 | SOD2 | Superoxide dismutase | 33 | 0.00738296 | 0.53793103 |
| 63 | PTGS1 | Prostaglandin G/H synthase 1 | 15 | 0.00150371 | 0.48148148 |
| 64 | CYP3A4 | Cytochrome P450 3A4 | 29 | 0.02163949 | 0.52702703 |
| 65 | UGT2B7 | UDP-glucuronosyltransferase 2B7 | 12 | 0.00179289 | 0.4116095 |
| 66 | CYP11B2 | Cytochrome P450 11B2 | 8 | 0.000658 | 0.42391304 |
| 67 | HSD11B2 | 11-beta-hydroxysteroid dehydrogenase type 2 | 4 | 0.0000103 | 0.36619718 |
| 68 | PIK3CD | Phosphatidylinositol 4,5-bisphosphate 3-kinase catalytic subunit delta isoform | 16 | 0.00499554 | 0.46706587 |
| 69 | MMP14 | Matrix metalloproteinase-14 | 20 | 0.000303 | 0.4952381 |
| 70 | BCL2 | Apoptosis regulator Bcl-2 | 16 | 0.000554 | 0.46428571 |
| 71 | BAX | Apoptosis regulator BAX | 16 | 0.000601 | 0.47416413 |
| 72 | PTGES2 | Prostaglandin E synthase 2 | 7 | 0.0000989 | 0.42162162 |
| 73 | PGR | Progesterone receptor | 32 | 0.00488456 | 0.53061224 |
| 74 | PTPN2 | Tyrosine-protein phosphatase non-receptor type 2 | 13 | 0.000278 | 0.47129909 |
| 75 | MMP8 | Neutrophil collagenase | 16 | 0.00201512 | 0.48297214 |
| 76 | CYP19A1 | Aromatase | 24 | 0.01009452 | 0.52 |
| 77 | LCK | Tyrosine-protein kinase Lck | 31 | 0.00290511 | 0.52 |
| 78 | ADRA2C | Alpha-2C adrenergic receptor | 17 | 0.00286472 | 0.46846847 |
| 79 | CNR1 | Cannabinoid receptor 1 | 22 | 0.00353481 | 0.50649351 |
| 80 | MMP1 | Interstitial collagenase | 31 | 0.00272702 | 0.52348993 |
| 81 | NTRK1 | High affinity nerve growth factor receptor | 17 | 0.000889 | 0.48598131 |
| 82 | ICAM1 | Intercellular adhesion molecule 1 | 45 | 0.0065162 | 0.55516014 |
| 83 | MMP3 | Stromelysin-1 | 28 | 0.00189912 | 0.50649351 |
| 84 | KAT2A | Histone acetyltransferase KAT2A | 8 | 0.0000567 | 0.44444444 |
| 85 | G6PD | Glucose-6-phosphate 1-dehydrogenase | 19 | 0.00808663 | 0.49681529 |
| 86 | GCK | Hexokinase-4 | 16 | 0.00472812 | 0.48297214 |
| 87 | VEGFB | Vascular endothelial growth factor B | 12 | 0.00111643 | 0.47272727 |
| 88 | CES1 | Metallothionein-2 | 12 | 0.00176751 | 0.41935484 |
| 89 | ALOX12 | Polyunsaturated fatty acid lipoxygenase ALOX12 | 8 | 0.000317 | 0.45348837 |
| 90 | HK2 | Hexokinase-2 | 15 | 0.00195511 | 0.47852761 |
| 91 | BRD2 | Bromodomain-containing protein 2 | 5 | 0.0001 | 0.4 |
| 92 | HPGDS | Hematopoietic prostaglandin D synthase | 36 | 0.01503815 | 0.54929577 |
| 93 | EZH2 | Histone-lysine N-methyltransferase EZH2 | 27 | 0.00294291 | 0.50485437 |
| 94 | CCR5 | C-C chemokine receptor type 5 | 22 | 0.00219833 | 0.5 |
| 95 | CCR2 | C-C chemokine receptor type 2 | 25 | 0.00287339 | 0.51147541 |
| 96 | NOD2 | Nucleotide-binding oligomerization domain-containing protein 2 | 16 | 0.000683 | 0.46428571 |
| 97 | NOD1 | Nucleotide-binding oligomerization domain-containing protein 1 | 10 | 0.0000527 | 0.4548105 |
| 98 | NR1H3 | Oxysterols receptor LXR-alpha | 11 | 0.00105016 | 0.4469914 |
| 99 | COL1A2 | Collagen alpha-2 chain | 14 | 0.000475 | 0.47852761 |
| 100 | HSD11B1 | 11-beta-hydroxysteroid dehydrogenase 1 | 10 | 0.00136351 | 0.45217391 |
| 101 | B2M | Beta-2-microglobulin | 16 | 0.00153798 | 0.48148148 |
| 102 | ENSG00000196689 | #N/A | 21 | 0.00487092 | 0.50160772 |
| 103 | F2 | Prothrombin | 33 | 0.01706854 | 0.53793103 |
| 104 | MPO | Myeloperoxidase | 36 | 0.00890475 | 0.54545455 |
| 105 | NR3C2 | Mineralocorticoid receptor | 10 | 0.00148212 | 0.45747801 |
| 106 | AOC3 | Membrane primary amine oxidase | 5 | 0.000524 | 0.42276423 |
| 107 | ALDH2 | Aldehyde dehydrogenase | 5 | 0.000677 | 0.3880597 |
| 108 | AGTR1 | Type-1 angiotensin II receptor | 30 | 0.00847131 | 0.52348993 |
| 109 | AVPR2 | Vasopressin V2 receptor | 5 | 0.000175 | 0.42162162 |
| 110 | CTSB | Cathepsin B | 21 | 0.00540401 | 0.4952381 |
| 111 | TP73 | Tumor protein p73 | 11 | 0.000334 | 0.43943662 |
| 112 | RBP4 | Retinol-binding protein 4 | 10 | 0.0015047 | 0.45614035 |
| 113 | EDNRA | Endothelin-1 receptor | 16 | 0.00136754 | 0.4875 |
| 114 | ABCB1 | ATP-dependent translocase ABCB1 | 23 | 0.02435449 | 0.51147541 |
| 115 | CCR1 | C-C chemokine receptor type 1 | 18 | 0.00128807 | 0.48447205 |
| 116 | HTR1A | 5-hydroxytryptamine receptor 1A | 14 | 0.00295588 | 0.44571429 |
| 117 | HBB | Hemoglobin subunit beta | 5 | 0.000124 | 0.4073107 |
| 118 | CXCR3 | C-X-C chemokine receptor type 3 | 19 | 0.00190437 | 0.48598131 |
| 119 | FABP2 | Fatty acid-binding protein | 7 | 0.000775 | 0.4116095 |
| 120 | AVPR1A | Vasopressin V1a receptor | 9 | 0.000256 | 0.41823056 |
| 121 | HTR2A | 5-hydroxytryptamine receptor 2A | 15 | 0.0028403 | 0.48447205 |
| 122 | SHH | Sonic hedgehog protein | 24 | 0.00107041 | 0.49056604 |
| 123 | GCGR | Glucagon receptor | 12 | 0.00124933 | 0.43697479 |
| 124 | VDR | Vitamin D3 receptor | 19 | 0.00777022 | 0.51147541 |
| 125 | CYP24A1 | 1,25-dihydroxyvitamin D 24-hydroxylase | 4 | 0.01296555 | 0.4073107 |
| 126 | FFAR4 | Free fatty acid receptor 4 | 11 | 0.00143277 | 0.44444444 |
| 127 | PRSS1 | Serine protease 1 | 6 | 0.0000671 | 0.40519481 |
| 128 | PON1 | Serum paraoxonase/arylesterase 1 | 16 | 0.00425262 | 0.47852761 |
| 129 | CETP | Cholesteryl ester transfer protein | 6 | 0.000708 | 0.41269841 |
| 130 | SCD | Stearoyl-CoA desaturase | 14 | 0.00145379 | 0.47272727 |
| 131 | MTNR1B | Melatonin receptor type 1B | 10 | 0.000545 | 0.43697479 |
| 132 | IDE | Insulin-degrading enzyme | 7 | 0.000334 | 0.46290801 |
| 133 | GC | Vitamin D-binding protein | 7 | 0.00171898 | 0.39897698 |
| 134 | CTSL | Procathepsin L | 8 | 0.0000613 | 0.44318182 |
| 135 | SELP | P-selectin | 21 | 0.0013294 | 0.5 |
| 136 | PLA2G7 | Platelet-activating factor acetylhydrolase | 5 | 0.000236 | 0.41823056 |
| 137 | HNF4A | Hepatocyte nuclear factor 4-alpha | 34 | 0.01521597 | 0.54355401 |
| 138 | CA1 | Carbonic anhydrase 1 | 1 | 0 | 0.28996283 |
| 139 | UCP2 | Mitochondrial uncoupling protein 2 | 20 | 0.00315061 | 0.49367089 |
| 140 | SLCO1B1 | Solute carrier organic anion transporter family member 1B1 | 10 | 0.01394621 | 0.38902743 |
| 141 | SHBG | Sex hormone-binding globulin | 4 | 0.0000108 | 0.38902743 |
| 142 | MUC1 | Mucin-1 | 20 | 0.000306 | 0.49840256 |
| 143 | TRPC6 | Short transient receptor potential channel 6 | 2 | 0.00000517 | 0.39 |
| 144 | ZEB2 | Zinc finger E-box-binding homeobox 2 | 13 | 0.000336 | 0.46290801 |
| 145 | BCHE | Cholinesterase | 8 | 0.000616 | 0.43333333 |
| 146 | SLCO1B3 | Solute carrier organic anion transporter family member 1B3 | 7 | 0.000706 | 0.37772397 |
| 147 | GAD2 | Glutamate decarboxylase 2 | 6 | 0.000766 | 0.41935484 |
| 148 | SLC22A6 | Solute carrier family 22 member 6 | 3 | 0.0000236 | 0.34361233 |
| 149 | DPP4 | Dipeptidyl peptidase 4 | 17 | 0.00447819 | 0.49840256 |
| 150 | SLC5A1 | Sodium/glucose cotransporter 1 | 5 | 0.000333 | 0.3929471 |
| 151 | SLC16A1 | Monocarboxylate transporter 1 | 5 | 0.0000506 | 0.43575419 |
| 152 | KCNH2 | Potassium voltage-gated channel subfamily H member 2 | 4 | 0.000253 | 0.36533958 |
| 153 | PDE4D | cAMP-specific 3',5'-cyclic phosphodiesterase 4D | 2 | 0.00000644 | 0.38902743 |
| 154 | RRM2B | Ribonucleoside-diphosphate reductase subunit M2 B | 1 | 0 | 0.32231405 |
| 155 | PPARD | Peroxisome proliferator-activated receptor delta | 7 | 0.000298 | 0.42739726 |
| 156 | SLC22A12 | Solute carrier family 22 member 12 | 1 | 0 | 0.28057554 |
| 157 | CUL5 | Cullin-5 | 1 | 0 | 0.34437086 |
